# The first Q-Tube based high-pressure synthesis of anti-cancer active thiazolo[4,5-*c*]pyridazines via the [4 + 2] cyclocondensation of 3-oxo-2-arylhydrazonopropanals with 4-thiazolidinones

**DOI:** 10.1038/s41598-020-63453-2

**Published:** 2020-04-16

**Authors:** Hamada Mohamed Ibrahim, Haider Behbehani

**Affiliations:** 1 Chemistry Department, Faculty of Science, Kuwait University, P.O. Box 5969, Safat, 13060 Kuwait; 2 Chemistry Department, Faculty of Science, Fayoum University, P.O. Box, 63514 Fayoum, Egypt

**Keywords:** Cancer, Chemical biology

## Abstract

A novel and efficient protocol for the synthesis of thiazolo[4,5-*c*]pyridazine derivatives was developed. The approach utilizes a high pressure Q-Tube reactor to promote cyclocondensation reactions between 3-oxo-2-arylhydrazonopropanals and 4-thiazolidinones. The process has a significantly high atom economy and a broad substrate scope, as well as being applicable to gram scale syntheses. The *in vitro* cytotoxic activities of the synthesized thiazolo[4,5-*c*]pyridazine derivatives were examined utilizing a MTT colorimetric assay with doxorubicin as a reference anti-cancer drug and three human cancer cell lines including HCT-116 (colon), MCF-7 (breast) and A549 (lung). The results show that thiazolopyridazines 7c, h, k and p have high cytotoxic activity against the MCF-7 cell line with respective IC_50_ values of 14.34, 10.39, 15.43 and 13.60 μM. Moreover, the thiazolopyridazine derivative 7s also show promising cytotoxic activity against the HCT-116 cell line with IC_50_ = 6.90 μM . Observations made in this effort serve as a basis for further investigations into the design and preparation of new anti-cancer drugs.

## Introduction

**Scheme 1:**
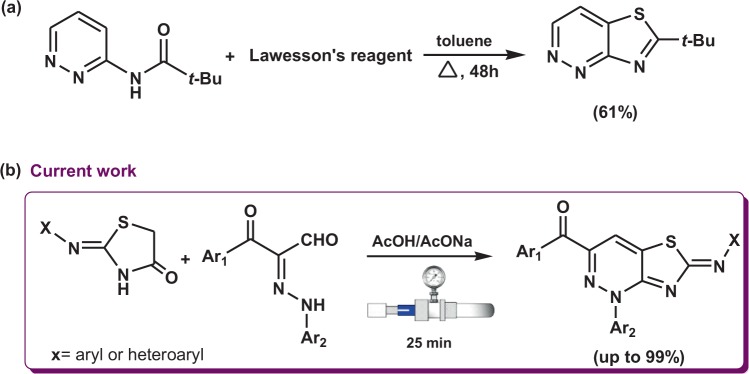
(**a**) Previous and (**b**) new route for synthesis of thiazolo[4,5-*c*]pyridazine derivatives.

Thiazolopyridazine derivatives comprise a broad range of structurally interesting substances that display a variety of medicinally interesting properties including activities against cancers^1,2^, microbes^3,4^, viruses^5^ and bacteria^6^, as well as antioxidant^7^, analgesic and pesticidal activities^8,9^. As a result of these important properties, thiazolopyridazines remain the focus of our continuing investigations aimed at developing new and green routes for the synthesis of novel fused nitrogen containing heterocycles^10–18^. Until now, several methods have been developed for the synthesis of fused thiazolopyridazines, most of which are targeted at the synthesis of thiazolo[4,5-*d*]pyridazines^1–3,5^, thiazolo[3,2-*b*]pyridazine^19^ and thiazolo[5,4-*c*]pyridazine^6^. In contrast, current methods to prepare thiazolo[4,5-*c*]pyridazines are much less well-developed^20^, and they suffer from some major deficiencies. Specifically, the only method devised for this purpose involves reaction of pyridazine-3-carboxamide derivatives with Lawesson’s reagent under reflux for 48 h, which forms mono-substituted thiazolo[4,5-*c*]pyridazines in only moderate yields (Scheme 1a)^20^.

In the current study, we developed a novel and efficient green method for the synthesis members of a series o thiazolo[4,5-*c*]pyridazine derivatives. The new protocol, which involves cyclocondensation reactions between 4-thiazolidinones and 3-oxo-2-arylhydrazonopropanals utilizing a high pressure Q-Tube reactor, is simple and environmentally benign (Scheme 1b). In contrast to those utilizing microwave irradiation and conventional heating, processes carried out using the Q-Tube reactor employ a high pressures to promote organic reactions. Use of the Q-tube technique has many advantageous features^21–28^ including higher yields, clean products, lower energy costs, lower reaction times and higher reproducibility than those associated with conventional open flask and microwave approaches, and safer conditions than those utilized in conventional sealed pressure tube processes.

## Results and Discussion

The initial phase of the current investigation, which was aimed at developing a new method for synthesis of thiazolo[4,5-*c*]pyridazine derivatives utilizing cyclocondensation reactions between 4-thiazolidinones and 3-oxo-2-arylhydrazonopropanals, concentrated on the preparation of 4-thiazolidinones. We observed that by using a slight modification of a two-step procedure described previously^29^, aromatic or heteroaromatic amines **1a-c** react with chloroacetyl chloride **2** to form the corresponding chloroacetyl derivatives **3a-c**, which can be transformed to the respective 4-thiazolidinones **4a–c** by reaction with ammonium thiocyanate in ethanol or dioxane under pressurized conditions utilizing a Q-Tube reactor.

**Figure 1:**
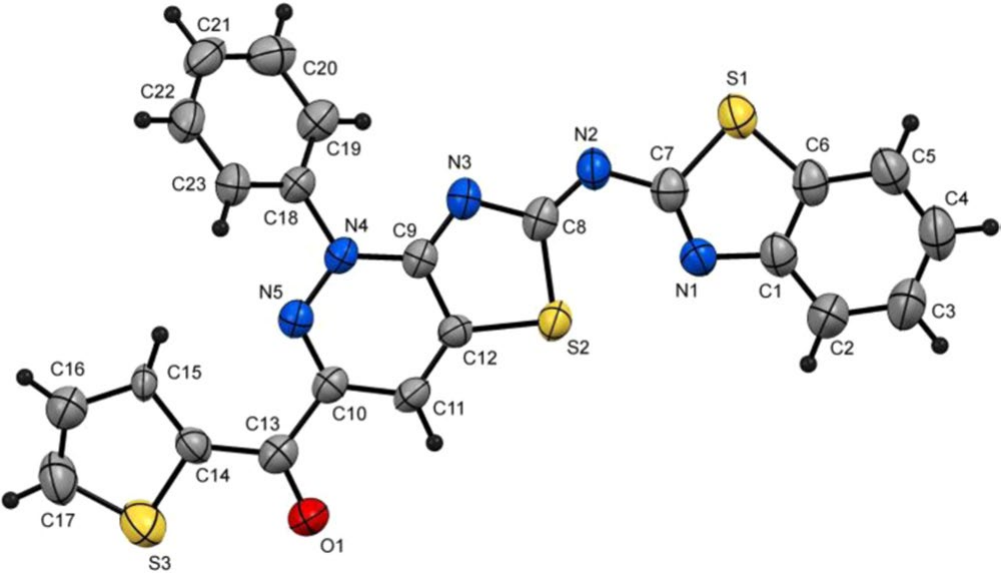
Plot of X-ray single crystallographic data collected for **7m**. Mercury (version 4.3.1) (https://www.ccdc.cam.ac.uk/solutions/csd-system/components/mercury/) was used to create this figure.

Next, reaction of the 2-(benzothiazol-2-ylimino)thiazolidin-4-one (**4a**) and 3-oxo-3-phenyl-2-(2-phenylhydrazono)propanal (**5a**) was used as a model to explore procedures and conditions for preparing the target thiazolo[4,5-*c*]pyridzines. We observed that stirring solutions of **4a** (5 mmol) and **5a** (5 mmol) in ethanol, dioxane, acetonitrile or DMF (15 mL) containing ammonium acetate or anhydrous sodium acetate (10 mmol) at atmospheric pressure and reflux for 12 h does not lead to formation of any products (Table 1, entries 1–4). In contrast, stirring a mixture of **4a** (5 mmol), **5a** (5 mmol) and ammonium acetate (10 mmol) in acetic acid (10 mL) at reflux for 4 h leads to production of thiazolo[4,5-*c*]pyridazine **7a** in 43% yield (Table 1, entry 5). The structure of the product was assigned as being **7a** rather than that of the thiazolo[4,5-*b*]pyridine **6** (Scheme 3) using spectrometric analysis. For example, the mass spectrum of the product showed that it has an exact mass of m/z 465.0717 that corresponds to the atomic composition of C_25_H_15_N_5_OS_2_. The ^1^H NMR spectrum of **7a** in DMSO-*d*_6_ contains a set of resonances in the 7.21–8.00 ppm region associated with the pyridazine H-4 and fourteen aromatic protons and is devoid of a NH signal. Moreover, the ^13^C{^1^H} NMR, spectra of **7a** contains 20 signals in addition to a characteristic carbonyl resonance at 191.88 ppm. Finally, X-ray crystallographic analysis of a related substance **7m** (see Fig. 1 below) enabled unambiguous assignment of the structure of **7a**.

**Table 1:** Optimization of reaction of 4-thiazolidinone **4a** and arylhydrazonal **5a**.

Entry	Solvent	Additive	T (°C)	Time	Product (% Yield)
**1**	EtOH	AcONH_4_ or AcONa	reflux	12 h	**—**
**2**	1,4-dioxane	AcONH_4_ or AcONa	reflux	12 h	**—**
**3**	CH_3_CN	AcONH_4_ or AcONa	reflux	12 h	**—**
**4**	DMF	AcONH_4_ or AcONa	reflux	12 h	**—**
**5**	AcOH	AcONH_4_	reflux	4 h	43^a^
**6**	AcOH	AcONH_4_	MW(125 °C, 250 W)	25 min	65^b^
**7**	AcOH	AcONH_4_	Q-tube(150 °C)	30 min	81^a^
**8**	AcOH	AcONa	MW(130 °C, 250 W)	30 min	73
**9**	AcOH	AcONa	Q-tube(150 °C)	30 min	86
**10**	AcOH	AcONa	Q-tube(160 °C)	30 min	92
**11**	AcOH	AcONa	Q-tube(170 °C)^c^	25 min	98

Reaction conditions: ^a^4-thiazolidinone **4a** (5 mmol), arylhydrazonal **5a** (5 mmol) and additive (10 mmol) in solvent (10 mL), ^b^4-thiazolidinone **4a** (2 mmol), arylhydrazonal **5a** (2 mmol) and additive (4 mmol) in solvent (5 mL). ^c^Temperature of the oil bath.

The mechanism of this cycloaddition process illustrated in Scheme 4 involves two successive condensation reactions. In the route, the enol formed from the 4-thiazolidinone **4** by acetic acid stimulated enolization adds to the aldehyde carbonyl carbon to generate adduct **A** which undergoes dehydration process to produce the corresponding alkylidene intermediate **B**. Next, a nucleophilic addition of the hydrazine NH moiety to the 4-thiazolidinone carbonyl carbon generates adduct **C** that undergoes dehydration to produce the thiazolo[4,5-*c*]pyridazine **7**.

The observations made above encouraged a more detailed study of the cyclocondensation reaction in order to uncover ideal conditions for the efficient synthesis of thiazolo[4,5-*c*]pyridazine **7a**, especially those that utilize a Q-tube pressure reactor. As mentioned above, Q-tube reactors are economic alternatives to expensive microwave (MW) systems in that they can be employed to promote high pressure chemical reactions safely. For comparison purposes, we first carried out reaction of 4-thiazolidinone **4a** (2 mmol), arylhydrazonal **5a** (2 mmol) and ammonium acetate (4 mmol) in acetic acid (4 mL) in a 10 mL MW tube which was irradiated at 125 °C (250 watt, 25 min). This process produces **7a** in 65% yield (Table 1, entry 6). On the other hand, a larger scale reaction of [**4a** (5 mmol), **5a** (5 mmol) and ammonium acetate (10 mmol) in acetic acid (10 mL)] conducted in a 35 mL borosilicate glass tube in a Q-tube reactor at 150 °C for 30 min forms **7a** in a much higher 81% yield (Table 1, entry 7). The noticeable enhancement in the yield of this reaction when a Q-tube reactor is employed may in part be a consequence of the fact that a temperature higher than the boiling points of the solvent and reagents is used and this leads to increase the reaction rate^30,31^. In addition, the increased pressure achievable using a Q-tube reactor reduces the volume of the reaction, thus increasing the effective concentrations of the reactants and their collision frequency, so the competing degradations of the reactants is minimized, allowing more clean reaction pattern. After clearly demonstrating the effect of using the Q-tube reactor on the efficiency of the thiazolo[4,5-*c*]pyridazine forming reaction (Table 1, entry 7), we assessed the effects of additives and temperature on the process. The results showed that the use of anhydrous sodium acetate rather than ammonium acetate and acetic acid as solvent is optimal (Table 1, entry 9). Also, we observed that temperature plays a role in determining the efficiency of this reaction. Accordingly, reactions performed at 150, 160 and 170 °C lead to formation of **7a** in 86, 92 and 98% respective yields, (Table 1, entries 9–11).

**Scheme 2:**
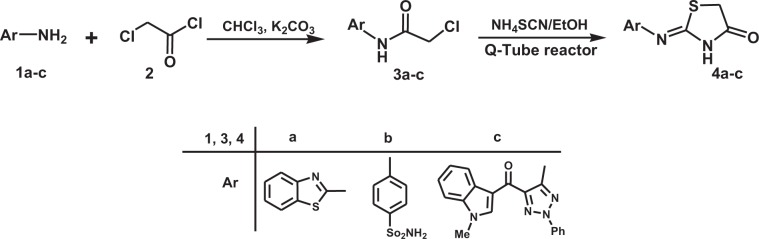
Synthesis of the 4-thiazolidinones **4a–c**.

The substrate scope of the novel cyclocondensation reaction, conducted using the conditions described in entry 11 of Table 1, was explored next. An assortment of 4-thiazolidinones **4a-c**, prepared by using the same sequence illustrated in Scheme 2, and several 3-oxo-2-arylhydrazonopropanals **5**, containing electron-withdrawing or -donating groups on both aryl substituents were used for this purpose. The results depicted in Table 2 show that reactions between **4a−c** and **5** produce the corresponding thiazolo[4,5-*c*]pyridazine derivatives **7a−u** in high yields and, consequently, that the existence of either electron-donating or electron-withdrawing substituents on both aryl moieties in the arylhydrazonal substrate has no effect on the efficiency of the new cyclocondensation process. The thiazolo[4,5-*c*]pyridazine **7 m** (Fig. 1), generated in this exploratory effort was used to carry out X-ray crystallographic analysis to prove the assigned structures to the products and show that only the (*Z*)-isomer of 6-(benzothiazol-2-ylimino)-1,6-dihydrothiazolo[4,5-*c*]pyridazine **7** is formed in the reactions.

**Scheme 3:**
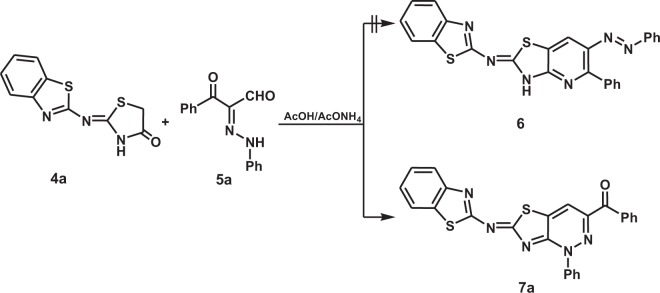
Reaction of 4-thiazolidinone **4a** and arylhydrazonal **5a**.

**Scheme 4:**
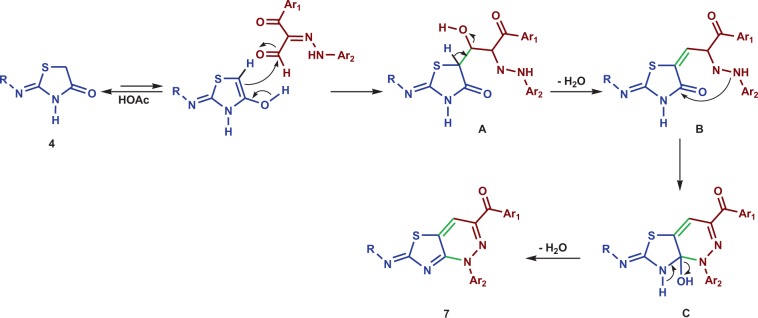
Plausible mechanistic pathway for the formation of thiazolo[4,5-*c*]pyridazine **7**.

**Table 2:** Q-tube [4 + 2] cyclocondensation reactions of 4-thiazolidinone **4a-c** and arylhydrazonal **5**.


**Entry**	**Reactants**	**Ar**_**1**_	**Ar**_**2**_	**X**	**Product**	**Yield (%)**^a^
**1**	**4a + 5a**	C_6_H_4_	C_6_H_5_			**98**
**2**	**4a + 5b**	C_6_H_4_	4-Cl-C_6_H_4_			**99**
**3**	**4a + 5c**	C_6_H_5_	4-NO_2_-C_6_H_4_			**94**
**4**	**4a+5d**	C_6_H_5_	4-MeO-C_6_H_4_			**95**
**5**	**4a + 5e**	4-Cl-C_6_H_4_	4-Cl-C_6_H_4_			**96**
**6**	**4a** + **5 f**	4-Cl-C_6_H_4_	2-Cl-5-NO_2_-Ph			**98**
**7**	**4a** + **5 g**	4-Br-C_6_H_4_	2-Cl-5-NO_2_-Ph			**97**
**8**	**4a** + **5 h**	4-Br-C_6_H_4_	2,4-diF-C_6_H_3_			**94**
**9**	**4a** + **5i**	4-F-C_6_H_4_	4-Me-C_6_H_4_			**92**
**10**	**4a** + **5j**	C_10_H_7_	4-Cl-C_6_H_4_			**96**
**11**	**4a** + **5k**	C_10_H_7_	4-NO_2_-C_6_H_4_			**95**
**12**	**4a** + **5 l**	C_10_H_7_	4-NO_2_-C_6_H_4_			**93**
**13**	**4a** + **5 m**	C_4_H_3_S	C_6_H_5_			**92**
**14**	**4a** + **5n**	C_4_H_3_S	4-Cl-C_6_H_4_			**93**
**15**	**4a + 5o**	C_4_H_3_O	2-Cl-5-NO_2_-Ph			**90**
**16**	**4a + 5p**	*N*-Me-Ind	4-NO_2_-C_6_H_4_			**91**
**17**	**4b + 5a**	C_6_H_5_	C_6_H_5_			**91**
**18**	**4b + 5e**	4-Cl-C_6_H_4_	4-Cl-C_6_H_4_			**94**
**18**	**4b + 5c**	C_6_H_5_	4-NO_2_-C_6_H_4_			**92**
**19**	**4c + 5a**	C_6_H_5_	C_6_H_5_			**88**
**20**	**4c + 5b**	C_6_H_5_	4-Cl-C_6_H_4_			**89**

^a^Reaction conditions: a mixture 4-thiazolidinone **4a-c** (5 mmol), arylhydrazonal **5** (5 mmol), anhydrous sodium acetate (10 mmol) and glacial acetic acid (10 mL) was charged in the glass tube of the Q-tube reactor and heated in an oil bath at 170 °C for 25 min.

### *In vitro* anti-cancer screening

**Figure 2:**
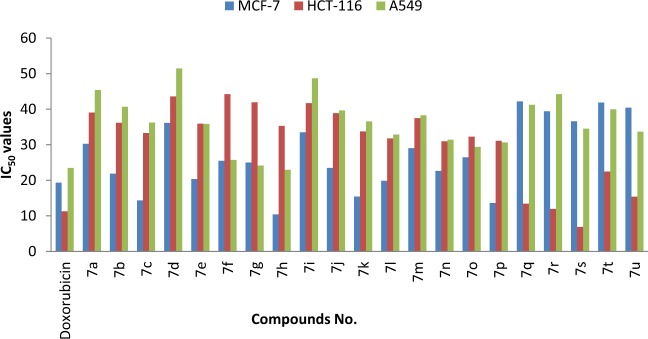
IC_50_ (μM) of **7a-u** towards human cancer cell lines.

Owing to the general propensity of thiazolopyridazine derivatives to display interesting properties, we have embrked on a program to assess potential biological activities of the substances synthesized using the process described above. In the preliminary phase of this effort, we evaluated the cytotoxic activities of **7a-u** towards three human cancer cell lines including HCT-116 (colon cancer), MF-7 (breast cancer) and A549 (lung cancer), in addition to the normal human cell line MCF10A (breast cell line). This assessment was performed using the standard MTT [3-(4,5-dimethylthiazol-2-yl)-2,5-diphenyltetrazolium bromide] colorimetric assay^32,33^, and utilizes doxorubicin as a reference anti-cancer agent^34–38^. For this purpose, three independent determinations were made using treatments with three concentrations (12.5, 25 and 50 μM) of **7a-u** for a 48 h incubation period. Analysis of the results of the MTT assays gave thew IC_50_ values for **7a-u** that are listed in Table 3 and Fig. 2. Inspection of the data shows that in comparison with doxorubicin the thiazolopyridazine derivatives have good to excellent cytotoxic activities against the tested cancer cell lines with IC_50_ in the range of 6.90–51.46 μM (vs. 11.26–23.47 μM for doxorubicin). While all of the tested substances display IC_50_ values in the ten micromolar range, several trends in their activities are worthy of brief mention. Firstly with respect to the MCF-7 cell line, **7c**, **7 h, 7k** and **7p** show the highest cytotoxic activities with IC_50_ values 14.34, 10.39, 15.43 and 13.60 μM, respectively (vs. doxorubicin 19.35 μM), while compounds **7b**, **7e**, **7j**, **7 l** and **7n** exhibits comparable IC_50_ to the reference drug. In addition, the results show that **7a-p**, which contain benzothiazole moieties, have higher cytotoxicities than do **7q-s** and **7t,u**, which contain benzenesulfonamide and [5-(1-methyl-1*H*-indole-3-carbonyl)-2-phenyl-2*H*-[1,2,3]triazol-4-ylimino] moieties, respectively. Substituents on the 1-*N*-aryl group play a significant rule in governing cytotoxic activity, as highlighted by the fact that the presence of electron-withdrawing substituents enhance activity (F > NO_2_ > Cl) while electron-donating substituents on the *N*-aryl moiety lower activity (H > Me > OMe). Moreover, **7s** which possesses a benzenesulfonamide moiety and NO_2_ (electron-withdrawing) substituent on the 1-*N*-aryl group displays higher cytotoxic activity (IC_50_ = 6.90 μM) than doxorubicin (IC_50_ = 11.26 μM), against the HCT-116 cell line. Also, **7q**, **7r** and **7u** are potent against the HCT-116 cell line. Furthermore, the respective IC_50_ values of **7f-h** against the A549 lung cancer cell line are 25.73, 24.13 and 22.96 μM respectively, which reflects the combined importance of the benzothiazole moiety and two electron-withdrawing substituents on the *N*-aryl moiety, along with the two fluorine substituents which show better cytotoxicity. In order to decide whether the cytotoxic characteristics of the synthesized compounds in this investigation, has no fatal cytotoxic effect on the normal human cells, and mostly selective to the cancer cells, the compounds showed the highest cytotoxic profile against the used cancer cell line like **7c**, **7 h**, **7k**, **7p** and **7s**, are screened and evaluated utilizing the same procedure against the MCF-10A cell line (normal breast cell line). The obtained IC_50_ values for this non-malicious cell (MCF-10A), gave promising data since their values [**7c** (32.98 ± 1.11 μM), **7h** (38.69 ± 1.85 μM), **7k** (32.15 ± 1.47 μM), **7p** (35.64 ± 0.87 μM), **7s** (26.88 ± 1.59 μM)] were comparable or higher than IC_50_ (28.74 ± 1.38) of doxorubicin when it was applied to the same normal cell line.

**Table 3:** IC_50_ values (μM) for **7a-u** towards human cancer cell lines.

Compound	MCF-7	HCT-116	A549
**7a**	30.24 ± 1.50	39.05 ± 0.87	45.36 ± 2.13
**7b**	21.87 ± 1.08	36.18 ± 2.32	40.68 ± 1.58
**7c**	14.34 ± 1.51	33.28 ± 1.41	36.24 ± 1.95
**7d**	36.13 ± 0.89	43.57 ± 1.57	51.46 ± 1.66
**7e**	20.35 ± 0.97	35.94 ± 1.18	35.85 ± 1.42
**7f**	25.47 ± 1.23	44.23 ± 1.59	25.73 ± 1.40
**7g**	24.98 ± 2.13	41.94 ± 2.05	24.13 ± 1.64
**7h**	10.39 ± 1.22	35.28 ± 1.53	22.96 ± 1.08
**7i**	33.47 ± 1.78	41.66 ± 1.63	48.66 ± 1.37
**7j**	23.45 ± 1.92	38.87 ± 2.14	39.65 ± 1.82
**7k**	15.43 ± 1.75	33.75 ± 1.57	36.54 ± 0.95
**7l**	19.86 ± 1.36	31.77 ± 1.32	32.86 ± 1.42
**7m**	29.05 ± 0.87	37.48 ± 1.87	38.27 ± 2.24
**7n**	22.63 ± 1.45	30.96 ± 2.36	31.44 ± 1.79
**7o**	26.44 ± 1.48	32.27 ± 1.49	29.38 ± 1.48
**7p**	13.60 ± 2.02	31.08 ± 1.18	30.62 ± 1.32
**7q**	42.18 ± 1.67	13.42 ± 1.32	41.23 ± 1.81
**7r**	39.40 ± 1.89	11.94 ± 1.46	44.19 ± 1.62
**7s**	36.58 ± 0.78	6.90 ± 1.14	34.51 ± 1.08
**7t**	41.85 ± 2.23	22.47 ± 1.70	39.98 ± 1.24
**7u**	40.42 ± 1.81	15.39 ± 0.84	33.67 ± 1.22
**Doxorubicin**	19.35 ± 1.18	11.26 ± 0.98	23.47 ± 1.24

## Conclusion

In summary, the study described above led to the development of an efficient Q-tube reactor based high-pressure protocol for synthesizing unprecedented series of thiazolo[4,5-*c*]pyridazines *via* [4 + 2] cyclocondensation reactions between 3-oxo-2-arylhydrazonopropanals and 4-thiazolidinones. The process has a high functional group tolerance and atom economy, and it is performed using simple, safe and environmentally compatible conditions. The synthesized thiazolopyridazines were shown to possess a potent cytotoxicities against MCF-7 (breast), HCT-116 (colon), and  A549 (lung) cancer cell lines. The next target of this study in the future, after obtaining these promising primary anticancer activity results, is to conduct more comprehensive studies to determine how the newly prepared thiazolopyridazine derivatives work to promote cell death (the mode of action) and to optimize biological activities.

## Experimental

### General

Melting points were recorded on a Griffin melting point apparatus and are uncorrected. IR spectra were recorded using KBr disks and a Jasco FT-IR-6300 spectrophotometer. ^1^H NMR (400 MHz) or (600 MHz) and ^13^C{^1^H} NMR (100 MHz) or (150 MHz) spectra were recorded at 25 °C using DMSO-*d*_6_ or (TFA-*d*) as solvents with TMS as an internal standard on a Bruker DPX 400 or 600 super-conducting NMR spectrometer. Chemical shifts (*δ*) are reported in ppm. Low-resolution electron impact mass spectra [MS (EI)] and high-resolution electron impact mass spectra [HRMS (EI)] were performed using a high resolution GC-MS (DFS) thermo spectrometer at 70.1 eV and a magnetic sector mass analyzer. Following the courses of reactions and checking homogeneity of products were performed using thin layer chromatography (TLC). The Q-Tube assisted reactions were performed in a Q-tube pressure monitor safe reactor from Q Labtech (distributed by Sigma-Aldrich), equipped with stainless steel adapter attached with pressure gauge (300 psi), high pressure adapter (180 psi), a needle adapter, a borosilicate glass pressure tube (35 mL), Teflon sleeve, a PTFE faced silicon septa and a catch bottle. The X-ray crystallographic data were collected by using a Bruker X8 Prospector at room temperature by using Cu- Kα radiation. The structures were solved by using direct methods and expanded using Fourier techniques. The non-hydrogen atoms were refined anisotropically. The structures were solved and refined using the Bruker SHELXTL Software Package (Structure solution program- SHELXS-97 and Refinement program- SHELXL-97)^39^. Data were corrected for the absorption effects using the multi-scan method (SADABS). The crystal image was created by the software Mercury (version 4.3.1)^40^. Compounds **3a-c** and **4a-c** were prepared according to literature procedures with slight modification in case of** 4a-c**^29^. The three human cancer cell lines including HCT-116 (colon cancer), MCF-7 (breast cancer) and A549 (lung cancer) were obtained from the American Type Culture Collection (ATCC).

### General procedure for the preparation of 2-Chloro-*N*-(heteroaryl)acetamides 3a–c

Solutions of amines **1a–c** (10 mmol) and chloroacetyl chloride **2** (1.12 g, 10 mmol) in chloroform (for **3a,b**) or dioxane (for **3c**) (50 mL) containing K_2_CO_3_ (15 mmol) were stirred at reflux for 10 h. The solutions were concentrated *in vacuo* giving residues that were diluted with water (100 mL) and filtered. The solid product is then washed with 5% NaHCO_3_ and subsequently with water, dried and crystallized from appropriate solvent to furnish pure **3a–c**.

#### *N*-Benzothiazol-2-yl-2-chloroacetamide (3a)^29^

Recrystallized from EtOH as white crystals, yield: 96%, m.p. 145–146 °C; IR (KBr): 𝑣/cm^−1^ 3348 (NH), 1665 (CO); ^1^H-NMR (600 MHz, DMSO-*d*_6_): *δ* = 4.47 (s, 2 H, CH_2_), 7.32 (t, *J* = 7.6 Hz, 1 H, Ar-H), 7.45 (t, *J* = 7.6 Hz, 1 H, Ar-H), 7.77 (d, *J* = 7.6 Hz, 1 H, Ar-H), 7.99 (d, *J* = 7.6 Hz, 1 H, Ar-H), 12.74 (s, 1 H, NH); ^13^C{^1^H} NMR (150 MHz, DMSO-*d*_6_): *δ* = 43.0 (*C*H_2_), 121.2, 122.3, 124.3, 126.7, 131.9, 148.9, 158.1, 166.4; MS (EI): *m/z* (%) 227 (M^+^ + 1, 5.60), 226 (M^+^, 26.40). HRMS (EI): *m/z* calcd. for C_9_H_7_ClN_2_OS (M^+^) 225.9962, found 225.9963.

#### 2-Chloro-*N*-(4-sulfamoylphenyl)acetamide (3b)^41^

Recrystallized from EtOH as white crystals, yield: 95%, m.p. 216–217 °C; IR (KBr): 𝑣/cm^−1^ 3328, 3211, 3132 (NH_2_, NH), 1689 (CO); ^1^H-NMR (600 MHz, DMSO-*d*_6_): *δ* = 4.31 (s, 2 H, CH_2_), 7.29 (s, 2 H, NH_2_), 7.75–7.81 (m, 4 H, Ar-H), 10.68 (s, 1 H, NH); ^13^C{^1^H} NMR (150 MHz, DMSO-*d*_6_): *δ* = 43.6 (*C*H_2_), 119.0, 126.8, 138.9, 141.4, 165.2; MS (EI): *m/z* (%) 249 (M^+^ + 1, 30.95), 248 (M^+^, 81.25). HRMS (EI): *m/z* calcd. for C_8_H_9_ClN_2_O_3_S (M^+^) 248.0017, found 248.0016.

#### 2-Chloro-*N*-[5-(1-methyl-1*H*-indole-3-carbonyl)-2-phenyl-2*H*-[1,2,3]triazol-4-yl]acetamide (3c)

Recrystallized from dioxane as pale yellow crystal, yield: 93%, m.p. 218–219 °C; IR (KBr): 𝑣/cm^−1^ 3232 (NH), 1691, 1615 (2CO); ^1^H-NMR (600 MHz, DMSO-*d*_6_): *δ* = 3.96 (s, 3 H, CH_3_), 4.53 (s, 2 H, CH_2_), 7.31–7.34 (m, 2 H, Ar-H), 7.50 (t, *J* = 7.2 Hz, 1 H, Ar-H), 7.58 (d, *J* = 7.2 Hz, 1 H, Ar-H), 7.63 (t, *J* = 7.6 Hz, 2 H, Ar-H), 8.17 (d, *J* = 7.6 Hz, 2 H, Ar-H), 8.37 (d, *J* = 7.2 Hz, 1 H, Ar-H), 8.81 (s, 1 H, indole C-*H*), 10.85 (s, 1 H, NH); ^13^C{^1^H} NMR (150 MHz, DMSO-*d*_6_): *δ* = 33.4 (*C*H_3_), 66.3 (*C*H_2_), 110.8, 113.1, 118.9, 121.6, 122.7, 123.3, 126.6, 128.3, 129.7, 136.4, 137.1, 138.7, 140.2, 145.1, 164.1, 179.1; MS (EI): *m/z* (%) 394 (M^+^ + 1, 22.87), 393 (M^+^, 64.08). HRMS (EI): *m/z* calcd. for C_20_H_16_ClN_5_O_2_ (M^+^) 393.0987, found 393.0986.

### General Procedure for the Synthesis of 2-(Arylimino)thiazolidin-4-ones 4a–c

Borosilicate glass pressure tubes (35 mL) of the Labtech Q-tube were charged with 2-chloro-*N*-(heteroaryl or aryl)acetamides **3a–c** (10 mmol) and ammonium thiocyanate (15 mmol) in absolute ethanol (15 mL) (for **4a,b**) or dioxane (15 mL) (for **4c**). A PTFE faced silicon septa was placed on the top of the tubes and the appropriate cap andpressure adapter were used. The mixtures were heated in an oil bath at 130 °C for 30 min. The course of each reaction was monitored by using TLC and GC/MS. The reaction mixtures were cooled to room temperature, and the formed solids were separated by filtration, washed with water and then recrystallized from the appropriate solvent.

#### (*Z*)-2-(Benzothiazol-2-ylimino)thiazolidin-4-one (4a)^29^

Recrystallized from an EtOH/dioxane (1:1) mixture pale yellow crystals, yield: 87%, m.p. 201–202 °C; IR (KBr): 𝑣/cm^−1^ 3145 (NH), 1734 (CO); ^1^H-NMR (600 MHz, DMSO-*d*_6_): *δ* = 4.12 (s, 2 H, CH_2_), 7.39 (t, *J* = 7.6 Hz, 1 H, Ar-H), 7.51 (t, *J* = 7.6 Hz, 1 H, Ar-H), 7.85 (d, *J* = 7.6 Hz, 1 H, Ar-H), 8.01 (d, *J* = 7.6 Hz, 1 H, Ar-H), 12.35 (s, 1 H, NH); ^13^C{^1^H} NMR (150 MHz, DMSO-*d*_6_): *δ* =35.7 (*C*H_2_), 121.8, 122.4, 124.7, 126.8, 133.5, 151.3, 166.7, 169.3, 174.8; MS (EI): *m/z* (%) 250 (M^+^ + 1, 14.60), 249 (M^+^, 100). HRMS (EI): *m/z* calcd. for C_10_H_7_N_3_OS_2_ (M^+^) 249.0025, found 249.0025.

#### (Z)-4-(4-oxothiazolidin-2-ylideneamino)benzenesulfonamide (4b)^41^

Recrystallized from dioxane as beige crystals, yield: 94%, m.p. 245–246 °C; IR (KBr): 𝑣/cm^−1^ 3358, 3271, 3199 (NH_2_, NH), 1676 (CO); ^1^H-NMR (600 MHz, DMSO-*d*_6_): *δ* = 4.04 (s, 2 H, CH_2_), 7.10–7.11 (m, 1 H, Ar-H), 7.33 (s, 2 H, NH_2_), 7.80–7.82 (m, 3 H, Ar-H), 11.94 (s, 1 H, NH); ^13^C{^1^H} NMR (150 MHz, DMSO-*d*_6_): *δ* =34.4 (*C*H_2_), 119.9, 121.7, 127.1, 139.6, 162.3, 174.2; MS (EI): *m/z* (%) 272 (M^+^ + 1, 24.92), 271 (M^+^, 100). HRMS (EI): *m/z* calcd. for C_9_H_9_N_3_O_3_S_2_ (M^+^) 271.0080, found 271.0079.

#### (Z)-2-[5-(1-methyl-1*H*-indole-3-carbonyl)-2-phenyl-2*H*-1,2,3-triazol-4-ylimino]thiazolidin-4-one (4c)

Recrystallized from dioxane/DMF mixture (3:1), as pale orange crystal, yield: 91%, m.p. 223–224 °C; IR (KBr): 𝑣/cm^−1^ 3322 (NH), 1691 (CO); ^1^H-NMR (600 MHz, DMSO-*d*_6_): *δ* = 3.99 (s, 3 H, CH_3_), 4.34 (s, 2 H, CH_2_), 7.30–7.38 (m, 2 H, Ar-H), 7.52 (t, *J* = 7.2 Hz, 1 H, Ar-H), 7.61–7.67 (m, 3 H, Ar-H), 8.18 (d, *J* = 7.6 Hz, 2 H, Ar-H), 8.37 (d, *J* = 7.2 Hz, 1 H, Ar-H), 8.81 (s, 1 H, indole C-*H*), 11.86 (s, 1 H, NH); ^13^C{^1^H} NMR (150 MHz, DMSO-*d*_6_): *δ* = 33.4 (*C*H_3_), 36.8 (*C*H_2_), 111.0, 112.7, 113.3, 119.0, 121.5, 122.7, 123.4, 126.6, 128.5, 129.8, 137.2, 138.7, 140.4, 144.6, 164.4, 178.9; MS (EI): *m/z* (%) 417 (M^+^ + 1, 9.28), 416 (M^+^, 33.25). HRMS (EI): *m/z* calcd. for C_21_H_16_N_6_O_2_S (M^+^) 416.1050, found 416.1050.

### General procedure for the preparation of thiazolo[4,5-*c*]pyridazine derivatives 7a-u

Borosilicate glass pressure tubes (35 mL) of the Labtech Q-tube were charged with 4-thiazolidinone **4a-c** (5 mmol), arylhydrazonal **5** (5 mmol), anhydrous sodium acetate (10 mmol) and glacial acetic acid (10 mL). A PTFE faced silicon septa was placed on the top of each tube and the appropriate cap and pressure adapter were used. The mixtures were heated in an oil bath at 170 °C for 25 min. The progress of each reaction was monitored by using TLC and GC/MS. The mixtures were cooled to room temperature, and the formed solids were separated by filtration, washed with ethanol and recrystallized from the appropriate solvent (see below) to give the thiazolo[4,5-*c*]pyridazine derivatives **7a-u** as pure products.

#### (*Z*)-{6-(Benzothiazol-2-ylimino)-1-phenyl-1,6-dihydrothiazolo[4,5-*c*]pyridazin-3-yl}phenymethanone (7a)

Recrystallized from dioxane, deep orange crystal, yield: 2.25 g (98%), m.p. 242–243 °C; IR (KBr): 𝑣/cm^−1^ 1700 (CO); ^1^H-NMR (600 MHz, DMSO-*d*_6_): *δ* = 7.22 (t, *J* = 7.2 Hz, 1 H, Ar-H), 7.35–7.38 (m, 2 H, Ar-H), 7.45 (t, *J* = 7.8 Hz, 2 H, Ar-H), 7.52 (t, *J* = 7.2 Hz, 1 H, Ar-H), 7.59 (t, *J* = 7.8 Hz, 2 H, Ar-H), 7.68–7.73 (m, 5 H, Ar-H), 7.78 (d, *J* = 7.2 Hz, 1 H, Ar-H), 8.00 (d, *J* = 7.2 Hz, 1 H, Ar-H); ^13^C{^1^H} NMR (150 MHz, DMSO-*d*_6_): *δ* = 115.9, 120.0, 121.2, 122.1, 124.3, 124.9, 126.4, 128.6, 128.8, 129.5, 130.2, 131.1, 132.7, 133.6, 137.0, 141.9, 151.0, 161.8, 166.9, 168.6, 191.9; MS (EI): *m/z* (%) 466 (M^+^ + 1, 15.12), 465 (M^+^, 45.25). HRMS (EI): *m/z* calcd. for C_25_H_15_N_5_OS_2_ (M^+^) 465.0713, found 465.0717.

#### (*Z*)-{6-(Benzothiazol-2-ylimino)-1-(4-chlorophenyl)-1,6-dihydrothiazolo[4,5-*c*]pyridazin-3-yl}phenylmethanone (7b)

Recrystallized from dioxane/DMF mixture (1:2), as reddish orange crystal, yield: 2.45 g (99%), m.p. 260–261 °C; IR (KBr): 𝑣/cm^−1^ 1700 (CO); ^1^H-NMR (600 MHz, TFA-*d*): *δ* = 7.57 (d, *J* = 7.8 Hz, 2 H, Ar-H), 7.61 (t, *J* = 7.8 Hz, 2 H, Ar-H), 7.70–7.76 (m, 4 H, Ar-H), 7.77–7.85 (m, 3 H, Ar-H), 7.94 (d, *J* = 8.4 Hz, 1 H, Ar-H), 8.06 (d, *J* = 8.4 Hz, 1 H, Ar-H), 8.21 (s, 1 H, pyridine- *H4*); ^13^C{^1^H} NMR (150 MHz, TFA-*d*): *δ* = 115.4, 118.9, 121.6, 125.2, 129.5, 130.85, 130.88, 131.6, 131.7, 132.6, 133.0, 136.6, 137.1, 137.96, 137.98, 138.1, 141.7, 170.2, 1 71.7, 174.5, 197.6; MS (EI): *m/z* (%) 501 (M^+^ + 2, 36.57), 500 (M^+^ + 1, 24.89), 499 (M^+^, 87.25). H RMS (EI): *m/z* calcd. for C_25_H_14_ClN_5_OS_2_ (M^+^) 499.0323, found 499.0323.

#### (*Z*)-{6-(Benzothiazol-2-ylimino)-1-(4-nitrophenyl)-1,6-dihydrothiazolo[4,5-*c*]pyridazin-3-yl}phenylmethanone (7c)

Recrystallized from EtOH/DMF mixture (1:3), as reddish brown crystal, yield: 2.40 g (94%), m.p. 257–258 °C; IR (KBr): 𝑣/cm^−1^ 1704 (CO); ^1^H-NMR (600 MHz, TFA-*d*): *δ* = 7.63 (t, *J* = 7.8 Hz, 2 H, Ar-H), 7.76–7.80 (m, 4 H, Ar-H  ), 7.83 (t, *J* = 8.4 Hz, 1 H, Ar-H), 7.95–7.99 (m, 3 H, Ar-H), 8.13 (d, *J* = 8.4 Hz, 1 H, Ar-H), 8.18 (s, 1 H, pyridine- *H4*), 8.51 (d, *J* = 7.8 Hz, 2 H, Ar-H); ^13^C{^1^H} NMR (150 MHz, TFA-*d*): *δ* = 118.7, 119.7, 125.5, 128.5, 129.7, 131.20, 131.24, 131.9, 133.2, 134.6, 136.1, 137.3, 137.9, 138.2, 147.5, 149.3, 170.1, 171.9, 173.9, 197.5; MS (EI): *m/z* (%) 511 (M^+^ + 1, 17.89), 510 (M^+^, 57.78). HRMS (EI): *m/z* calcd. for C_25_H_14_N_6_O_3_S_2_ (M^+^) 510.0563, found 510.0563.

#### (*Z*)-{6-(Benzothiazol-2-ylimino)-1-(4-methoxyphenyl)-1,6-dihydrothiazolo[4,5-*c*]pyridazin-3-yl}phenylmethanone (7d)

Recrystallized from dioxane/DMF mixture (3:1), as reddish brown crystal, yield: 2.35 g (95%), m.p. 250–251 °C; IR (KBr): 𝑣/cm^−1^ 1707 (CO); ^1^H-NMR (600 MHz, TFA-*d*): *δ* = 4.04 (s, 3 H, O-*CH*_3_), 7.24 (d, *J* = 9.0 Hz, 2 H, Ar-H), 7.59 (t, *J* = 7.8 Hz, 2 H, Ar-H), 7.67 (d, *J* = 7.8 Hz, 2 H, Ar-H), 7.70–7.74 (m, 2 H, Ar-H), 7.81 (t, *J* = 7.8 Hz, 1 H, Ar-H), 7.87 (d, *J* = 9.0 Hz, 2 H, Ar-H), 7.90 (d, *J* = 7.8 Hz, 1 H, Ar-H), 8.07 (d, *J* = 7.8 Hz, 1 H, Ar-H), 8.21 (s, 1 H, pyridine- *H4*); ^13^C{^1^H} NMR (150 MHz, TFA-*d*): *δ* = 58.1 (O*C*H_3_), 113.7, 118.3, 118.8, 122.6, 125.3, 129.5, 130.7, 130.8, 130.9, 131.7, 133.0, 136.4, 137.4, 138.00, 138.2, 139.0, 162.2, 170.4, 171.7, 174.5, 197.8; MS (EI): *m/z* (%) 496 (M^+^ + 1, 22.45), 495 (M^+^, 67.28). HRMS (EI): *m/z* calcd. for C_26_H_17_N_5_O_2_S_2_ (M^+^) 495.0818, found 495.0818.

#### (*Z*)-{6-(Benzothiazol-2-ylimino)-1-(4-chlorophenyl)-1,6-dihydrothiazolo[4,5-*c*]pyridazin-3-yl}(4-chlorophenyl)methanone (7e)

Recrystallized from dioxane/DMF mixture (1:1), as reddish orange crystal, yield: 2.55 g (96%), m.p. above 300 °C; IR (KBr): 𝑣/cm^−1^ 1705 (CO); ^1^H-NMR (600 MHz, TFA-*d*): *δ* = 7.41–7.44 (m, 4 H, Ar-H), 7.52 (d, *J* = 8.4 Hz, 2 H, Ar-H), 7.61–7.66 (m, 3 H, Ar-H), 7.70 (t, *J* = 8.4 Hz, 1 H, Ar-H), 7.80 (d, *J* = 8.4 Hz, 1 H, Ar-H), 7.92 (d, *J* = 8.4 Hz, 1 H, Ar-H), 8.02 (s, 1 H, pyridine- *H4*); ^13^C{^1^H} NMR (150 MHz, TFA-*d*): *δ* = 115.8, 119.0, 121.8, 125.3, 129.6, 131.0, 131.5, 132.2, 132.5, 132.7, 133.2, 136.6, 137.3, 137.4, 138.1, 141.8, 143.9, 170.3, 171.8, 174.5, 196.0; MS (EI): *m/z* (%) 535 (M^+^ + 2, 53.18), 534 (M^+^ + 1, 20.85), 533 (M^+^, 74.09). HRMS (EI): *m/z* calcd. for C_25_H_13_Cl_2_N_5_OS_2_ (M^+^) 532.9933, found 532.9934.

#### (*Z*)-{6-(Benzothiazol-2-ylimino)-1-(2-chloro-5-nitrophenyl)-1,6-dihydrothiazolo[4,5-*c*]pyridazin-3-yl}(4-chlorophenyl)methanone (7 f)

Recrystallized from DMF, as reddish orange crystal, yield: 2.80 g (98%), m.p. above 300 °C; IR (KBr): 𝑣/cm^−1^ 1711 (CO); ^1^H-NMR (600 MHz, TFA-*d*): *δ* = 7.51 (d, *J* = 8.4 Hz, 2 H, Ar-H), 7.63–7.66 (m, 3 H, Ar-H), 7.69–7.74 (m, 2 H, Ar-H), 7.82 (d, *J* = 8.4 Hz, 1 H, Ar-H), 8.07–8.09 (m, 2 H, Ar-H), 8.17 (d, *J* = 8.4 Hz, 1 H, Ar-H), 8.73 (d, *J* = 2.4 Hz, 1 H, Ar-H); ^13^C{^1^H} NMR (150 MHz, TFA-*d*): *δ* = 109.8, 115.2, 115.4, 120.3, 123.1, 126.8, 127.4, 128.7, 129.0, 129.2, 129.5, 130.6, 131.5, 132.1, 133.0, 134.8, 137.4, 140.9, 146.9, 166.6, 168.4, 168,8, 192.6; MS (EI): *m/z* (%) 580 (M^+^ + 2, 12.68), 579 (M^+^ + 1, 5.88), 578 (M^+^, 15.12). HRMS (EI): *m/z* calcd. for C_25_H_12_Cl_2_N_6_O_3_S_2_ (M^+^) 577.9784, found 577.9784.

#### (*Z*)-{6-(Benzothiazol-2-ylimino)-1-(2-chloro-5-nitrophenyl)-1,6-dihydrothiazolo[4,5-*c*]pyridazin-3-yl}(4-bromophenyl)methanone (7 g)

Recrystallized from DMF, as reddish orange crystal, yield: 3.00 g (97%), m.p. above 264–265 °C; IR (KBr): 𝑣/cm^−1^ 1715 (CO); ^1^H-NMR (600 MHz, TFA-*d*): *δ* = 7.64 (d, *J* = 8.4 Hz, 2 H, Ar-H), 7.74–7.83 (m, 5 H, Ar-H), 7.91 (d, *J* = 8.4 Hz, 1 H, Ar-H), 8.16–8.20 (m, 2 H, Ar-H), 8.26 (d, *J* = 8.4 Hz, 1 H, Ar-H), 8.88 (d, *J* = 2.4 Hz, 1 H, Ar-H); ^13^C{^1^H} NMR (150 MHz, TFA-*d*): *δ* = 112.9, 118.2, 118.4, 123.3, 126.0, 129.8, 130.4, 132.1, 132.2, 132.5, 133.6, 134.4, 134.8, 135.2, 136.4, 137.8, 140.5, 149.9, 167.0, 169.7, 171.6, 171.9, 196.0; MS (EI): *m/z* (%) 624 (M^+^ + 2, 100), 623 (M^+^ + 1, 31.89), 622 (M^+^, 69.75). HRMS (EI): *m/z* calcd. for C_25_H_12_BrClN_6_O_3_S_2_ (M^+^) 621.9279, found 621.9278.

#### (*Z*)-{6-(Benzothiazol-2-ylimino)-1-(2,4-difluorophenyl)-1,6-dihydrothiazolo[4,5-*c*]pyridazin-3-yl}(4-bromophenyl)methanone (7 h)

Recrystallized from dioxane/DMF mixture (1:1), as orange crystal, yield: 2.70 g (94%), m.p. above 261–262 °C; IR (KBr): 𝑣/cm^−1^ 1716 (CO); ^1^H-NMR (600 MHz, TFA-*d*): *δ* = 7.00–7.08 (m, 2 H, Ar-H), 7.50 (d, *J* = 8.4 Hz, 2 H, Ar-H), 7.65–7.68 (m, 3 H, Ar-H), 7.75 (t, *J* = 8.4 Hz, 1 H, Ar-H), 7.85 (d, *J* = 8.4 Hz, 1 H, Ar-H), 7.96 (d, *J* = 8.4 Hz, 1 H, Ar-H), 8.08–8.11 (m, 2 H, Ar-H); ^13^C{^1^H} NMR (150 MHz, TFA-*d*): *δ* = 107.7 (t, ^2^*J*_CF_ = 24 Hz), 115.0 (dd, ^2^ *J*_CF_ = 3.0, 24.0 Hz), 116.5, 119.0, 121.0 (d, ^3^*J*_CF_ = 10.5 Hz), 125.3, 128.4 (dd, ^3^*J*_CF_ = 4.5, 10.5 Hz), 129.6, 131.0, 132.0, 132.4, 132.5, 133.2, 135.2, 137.0 (d, ^3^*J*_CF_ = 9.0 Hz), 138.1, 156.0 (dd, *J*_CF_ = 12.0, 252.0 Hz), 163.9, 164.9 (dd, ^1^*J*_CF_ = 12.0, 252.0 Hz), 170.2, 171.8, 174.3, 196.3; MS (EI): *m/z* (%) 581 (M^+^ + 2, 100), 580 (M^+^ + 1, 24.73), 579 (M^+^, 85.89). HRMS (EI): *m/z* calcd. for C_25_H_12_Br F_2_N_5_OS_2_ (M^+^) 578.9629, found 578.9628.

#### (*Z*)-{6-(Benzothiazol-2-ylimino)-1-*p*-tolyl-1,6-dihydrothiazolo[4,5-*c*]pyridazin-3-yl}(4-fluorophenyl)methanone (7i)

Recrystallized from EtOH/DMF mixture (1:4), as red crystal, yield: 2.30 g (92%), m.p. 251–252 °C; IR (KBr): 𝑣/cm^−1^ 1708 (CO); ^1^H-NMR (600 MHz, TFA-*d*): *δ* = 2.31 (s, 3 H, *CH*_3_), 7.08 (t, *J* = 7.8 Hz, 2 H, Ar-H), 7.24 (d, *J* = 7.8 Hz, 2 H, Ar-H), 7.55–7.60 (m, 5 H, Ar-H), 7.67 (d, *J* = 7.8 Hz, 1 H, Ar-H), 7.76 (d, *J* = 7.8 Hz, 1 H, Ar-H), 7.83 (d, *J* = 7.8 Hz, 1 H, Ar-H), 7.98 (s, 1 H, pyridine- *H4*); ^13^C{^1^H} NMR (150 MHz, TFA-*d*): *δ* = 22.5 (*CH*_3_), 119.06, 119.11, 119.2, 120.8, 125.2, 129.7, 130.7, 130.9, 133.6, 134.1 (d, ^3^*J*_CF_ = 9 Hz), 134.7, 138.3 (d, ^2^*J*_CF_ = 24 Hz), 140.8, 143.5, 169.2 (d, ^3^*J*_CF_ = 10 Hz), 170.4, 171.7, 174.7, 195.8; MS (EI): *m/z* (%) 498 (M^+^ + 1, 27.12), 497 (M^+^, 100.00). HRMS (EI): *m/z* calcd. for C_26_H_16_FN_5_OS_2_ (M^+^) 497.0775, found 497.0775.

#### (*Z*)-{6-(Benzothiazol-2-ylimino)-1-(4-chlorophenyl)-1,6-dihydrothiazolo[4,5-*c*]pyridazin-3-yl}(naphthalen-1-yl)methanone (7j)

Recrystallized from dioxane/DMF mixture (1:1), as red crystal, yield: 2.60 g (96%), m.p. 254–255 °C; IR (KBr): 𝑣/cm^−1^ 1708 (CO); ^1^H-NMR (600 MHz, TFA-*d*): *δ* = 7.49–7.52 (m, 3 H, Ar-H), 7.59 (t, *J* = 7.2 Hz, 1 H, Ar-H), 7.63 (d, *J* = 7.8 Hz, 1 H, Ar-H), 7.69–7.72 (m, 3 H, Ar-H), 7.77–7.93 (m, 5 H, Ar-H), 7.98 (d, *J* = 7.2 Hz, 1 H, Ar-H), 8.16–8.21 (m, 2 H, Ar-H); ^13^C{^1^H} NMR (150 MHz, TFA-*d*): *δ* = 115.4, 118.8, 121.5, 125.0, 125.7, 129.4, 130.20, 130.22, 130.8, 131.5, 131.7, 131.9, 132.0, 132.5, 132.9, 133.2, 134.8, 135.1, 136.9, 137.7, 137.9, 138.4, 141.6, 170.0, 171.4, 174.3, 196.9; MS (EI): *m/z* (%) 551 (M^+^ + 2, 45.28), 550 (M^+^ + 1, 33.08), 549 (M^+^, 100.00). HRMS (EI): *m/z*  calcd. for C_29_H_16_ClN_5_OS_2_ (M^+^) 549.0479, found 549.0478.

#### (*Z*)-{6-(Benzothiazol-2-ylimino)-1-(4-nitrophenyl)-1,6-dihydrothiazolo[4,5-*c*]pyridazin-3-yl}(naphthalen-1-yl)methanone (7k)

Recrystallized from dioxane/DMF mixture (1:1), as red crystal, yield: 2.65 g (95%), m.p. 263–264 °C; IR (KBr): 𝑣/cm^−1^ 1708 (CO); ^1^H-NMR (600 MHz, TFA-*d*): *δ* = 7.61–7.64 (m, 3 H, Ar-H), 7.70 (d, *J* = 7.2 Hz, 1 H, Ar-H), 7.80 (t, *J* = 7.8 Hz, 1 H, Ar-H), 7.84–7.87 (m, 2 H, Ar-H), 7.93–8.03 (m, 5 H, Ar-H), 8.15 (t, *J* = 7.2 Hz, 2 H, Ar-H), 8.53 (d, *J* = 9.0 Hz, 2 H, Ar-H); ^13^C{^1^H} NMR (150 MHz, TFA-*d*): *δ* = 119.0, 120.0, 121.7, 123.8, 125.3, 125.4, 126.9, 128.4, 128.5, 129.2, 129.7, 130.2, 131.1, 132.4, 133.1, 135.2, 136.3, 138.0, 140.4, 147.6, 150.6, 154.1, 169.7, 171.6, 173.0, 173.7, 199.5; MS (EI): *m/z* (%) 561 (M^+^ + 1, 30.17), 560 (M^+^, 100.00). HRMS (EI): *m/z* calcd. for C_29_H_16_N_6_O_3_S_2_ (M^+^) 560.0720, found 560.0720.

#### (*Z*)-{ 6-(Benzothiazol-2-ylimino)-1-(4-nitrophenyl)-1,6-dihydrothiazolo[4,5-*c*]pyridazin-3-yl}(naphthalen-2-yl)methanone (7 l)

Recrystallized from dioxane/DMF mixture (1:1), as red crystal, yield: 2.60 g (93%), m.p. 259–260 °C; IR (KBr): 𝑣/cm^−1^ 1704 (CO); ^1^H-NMR (600 MHz, TFA-*d*): *δ* = 7.56 (t, *J* = 7.8 Hz, 1 H, Ar-H), 7.63 (t, *J* = 7.8 Hz, 1 H, Ar-H), 7.72 (d, *J* = 8.4 Hz, 1 H, Ar-H), 7.75 (t, *J* = 7.8 Hz, 1 H, Ar-H), 7.81 (t, *J* = 8.4 Hz, 1 H, Ar-H), 7.87 (d, *J* = 7.8 Hz, 1 H, Ar-H), 7.90–7.94 (m, 4 H, Ar-H), 7.98 (d, *J* = 8.4 Hz, 1 H, Ar-H), 7.10 (d, *J* = 8.4 Hz, 1 H, Ar-H), 8.22 (s, 1 H, Ar-H), 8.27 (s, 1 H, Ar-H), 8.45 (d, *J* = 9.0 Hz, 2 H, Ar-H); ^13^C{^1^H} NMR (150 MHz, TFA-*d*): *δ* = 118.6, 119.0, 125.3, 125.7, 128.4, 129.6, 130.3, 131.1, 131.7, 132.1, 132.4, 133.1, 133.8, 134.7, 134.9, 135.9, 138.1, 138.7, 147.3, 149.2, 169.9, 171.6, 173.7, 196.8; MS (EI): *m/z* (%) 561 (M^+^ + 1, 30.78), 560 (M^+^, 100.00). HRMS (EI): *m/z* calcd. for C_29_H_16_N_6_O_3_S_2_ (M^+^) 560.0720, found 560.0720.

#### (*Z*)-{6-(Benzothiazol-2-ylimino)-1-phenyl-1,6-dihydrothiazolo[4,5-*c*]pyridazin-3-yl}(thiophen-2-yl)methanone (7 m)

Recrystallized from EtOH/DMF mixture (1:2), as reddish brown crystal, yield: 2.15 g (92%), m.p. above 300 °C; IR (KBr): 𝑣/cm^−1^ 1713 (CO); ^1^H-NMR (600 MHz, TFA-*d*): *δ* = 7.33 (t, *J* = 4.8 Hz, 1 H, Ar-H), 7.62 (t, *J* = 7.8 Hz, 1 H, Ar-H), 7.73 (t, *J* = 7.8 Hz, 1 H, Ar-H), 7.82 (t, *J* = 8.4 Hz, 2 H, Ar-H), 7.88–7.90 (, 3 H, Ar-H), 8.07 (d, *J* = 4.8 Hz, 1 H, Ar-H), 8.11 (d, *J* = 8.4 Hz, 2 H, Ar-H), 8.53 (d, *J* = 4.8 Hz, 1 H, Ar-H), 9.29 (s, 1 H, pyridine-*H4*); ^13^C{^1^H} NMR (150 MHz, TFA-*d*): *δ* = 117.5, 124.7, 127.4, 128.5, 129.4, 129.7, 131.5, 132.0, 132.2, 134.6, 137.2, 139.3, 142.6, 143.7, 143.8, 147.5, 151.1, 162.4, 170.2, 180.4, 182.0; MS (EI): *m/z* (%) 472 (M^+^ + 1, 28.88), 471 (M^+^, 100.00). HRMS (EI): *m/z* calcd. for C_23_H_13_N_5_OS_3_ (M^+^) 471.0277, found 471.0276. Crystal Data, moiety formula: C_23_H_13_N_5_OS_3_, M = 471.56, monoclinic, a = 13.8134(7) Å, b = 9.7580(5) Å, c = 16.2524(7) Å, V = 2089.17(18) Å^3^, α = γ = 90°, β = 107.510(3)°, space group: P 21/n, Z = 4, D_calc_ = 1.499 g·cm^−3^, No. of reflection measured 3515, θ _max_ = 66.76°, R1 = 0.0756 (CCDC 1969668)^42^.

#### (*Z*)-{6-(Benzothiazol-2-ylimino)-1-(4-chlorophenyl)-1,6-dihydrothiazolo[4,5-*c*]pyridazin-3-yl}(thiophen-2-yl)methanone (7n)

Recrystallized from DMF, as reddish brown crystal, yield: 2.35 g (93%), m.p. above 300 °C; IR (KBr): 𝑣/cm^−1^ 1714 (CO); ^1^H-NMR (600 MHz, TFA-*d*): *δ* = 7.23 (t, *J* = 4.8 Hz, 1 H, Ar-H), 7.53 (t, *J* = 7.8 Hz, 1 H, Ar-H), 7.63 (t, *J* = 7.8 Hz, 1 H, Ar-H), 7.71 (d, *J* = 7.8 Hz, 1 H, Ar-H), 7.75–7.78 (m, 3 H, Ar-H), 7.97 (d, *J* = 4.8 Hz, 1 H, Ar-H), 8.00 (d, *J* = 9.0 Hz, 2 H, Ar-H), 8.40 (d, *J* = 4.8 Hz, 1 H, Ar-H), 9.14 (s, 1 H, pyridine-*H4*); ^13^C{^1^H} NMR (150 MHz, TFA-*d*): *δ* = 117.7, 124.9, 128.4, 128.9, 129.5, 129.9, 131.6, 132.1, 132.5, 137.3, 139.3, 141.6, 142.2, 142.6, 143.7, 147.9, 151.1, 162.5, 170.4, 180.9, 182.1; MS (EI): *m/z* (%) 507 (M^+^ + 2, 42.08), 506 (M^+^ + 1, 23.95), 505 (M^+^, 100.00). HRMS (EI): *m/z* calcd. for C_23_H_12_ClN_5_OS_3_ (M^+^) 504.9887, found 504.9889.

#### (*Z*)-{6-(Benzothiazol-2-ylimino)-1-(2-chloro-5-nitrophenyl)-1,6-dihydrothiazolo[4,5-*c*]pyridazin-3-yl}(furan-2-yl)methanone (7o)

Recrystallized from EtOH/DMF mixture (1:2), as reddish brown crystal, yield: 2.40 g (90%), m.p. 273–274 °C; IR (KBr): 𝑣/cm^−1^ 1716 (CO); ^1^H-NMR (600 MHz, TFA-*d*): *δ* = 6.82 (t, *J* = 4.8 Hz, 1 H, Ar-H), 7.71–7.74 (m, 3 H, Ar-H), 7.81 (t, *J* = 8.4 Hz, 1 H, Ar-H), 7.91–7.92 (m, 2 H, Ar-H), 8.11 (d, *J* = 9.0 Hz, 1 H, Ar-H), 8.22 (d, *J* = 8.4 Hz, 1 H, Ar-H), 8.69 (d, *J* = 2.4 Hz, 1 H, Ar-H), 8.78 (s, 1 H, pyridine-*H4*); ^13^C{^1^H} NMR (150 MHz, TFA-*d*): *δ* = 112.9, 116.6, 118.7, 119.3, 123.5, 126.5, 127.8, 130.2, 130.8, 132.3, 133.0, 134.0, 134.1, 135.0, 138.2, 140.8, 150.3, 152.9, 153.2, 170.0, 171.8, 172.2, 179.8; MS (EI): *m/z* (%) 536 (M^+^ + 2, 14.22), 535 (M^+^ + 1, 9.89), 534 (M^+^, 28.92). HRMS (EI): *m/z* calcd. for C_23_H_11_ClN_6_O_4_S_2_ (M^+^) 533.9966, found 533.9965.

#### (*Z*)-{6-(Benzothiazol-2-ylimino)-1-(4-nitrophenyl)-1,6-dihydrothiazolo[4,5-*c*]pyridazin-3-yl}(1-methyl-1*H*-indol-2-yl)methanone (7p)

Recrystallized from DMF, as deep orange crystal, yield: 2.55 g (91%), m.p. above 300 °C; IR (KBr): 𝑣/cm^−1^ 1700 (CO); ^1^H-NMR (600 MHz, TFA-*d*): *δ* = 4.40 (s, 3 H, C*H*_3_), 7.98–8.98 (m, 14 H, Ar-H); ^13^C{^1^H} NMR (150 MHz, TFA-*d*): *δ* = 35.7 (*C*H_3_), 113.6, 116.9, 118.9, 119.1, 119.7, 124.1, 125.5, 127.6, 128.3, 128.5, 129.7, 131.3, 133.2, 135.7, 138.0, 138.2, 141.3, 143.57, 146.3, 150.0 , 170.0, 171.8, 173.8, 187.0, 188.1; MS (EI): *m/z* (%) 564 (M^+^ + 1, 2.24), 563 (M^+^, 7.85). HRMS  (EI): *m/z* calcd. for C_28_H_17_N_7_O_3_S_2_ (M^+^) 563.0829, found 563.0829.

#### (*Z*)-4-{3-Benzoyl-1-phenylthiazolo[4,5-*c*]pyridazin-6(1*H*)-ylideneamino}benzenesulfonamide (7q)

Recrystallized from  dioxane/DMF mixture (3:1), as orange crystal, yield: 2.20 g (91%), m.p. 251–252 °C; IR (KBr): 𝑣/cm^−1^ 1699 (CO); ^1^H-NMR (600 MHz, DMSO-*d*_6_): *δ* = 7.00–7.98 (m, 17 H, 15Ar-H and SO_2_*NH*_2_); ^13^C{^1^H} NMR (150 MHz, DMSO-*d*_6_): *δ* = 115.6, 118.7, 120.3, 121.8, 123.2, 124.4, 127.0, 128.6, 128.8, 129.4, 130.1, 130.9, 132.8, 136.9, 140.2, 141.8, 172.0, 191.9; MS (EI): *m/z* (%) 488 (M^+^ + 1, 28.75), 487 (M^+^, 100.00). HRMS (EI): *m/z* calcd. for C_24_H_17_N_5_O_3_S_2_ (M^+^) 487.0757, found 487.0769.

#### *(Z*)-4-{3-(4-Chlorobenzoyl)-1-(4-chlorophenyl)thiazolo[4,5-*c*]pyridazin-6(1*H*)-ylideneamino}benzenesulfonamide (7r)

Recrystallized from dioxane/DMF mixture (1:1), as orange crystal, yield: 2.60 g (94%), m.p. 244–245 °C; IR (KBr): 𝑣/cm^−1^ 1699 (CO); ^1^H-NMR (600 MHz, TFA-*d*): *δ* = 7.39–7.43 (m, 4 H, 2Ar-H and SO_2_*NH*_2_), 7.60–7.65 (m, 6 H, Ar-H), 7.88 (d, *J* = 8.4 Hz, 2 H, Ar-H), 8.22 (s, 1 H, pyridine-*H4*), 8.35 (d, *J* = 8.4 Hz, 2 H, Ar-H); ^13^C{^1^H} NMR (150 MHz, TFA-*d*): *δ* = 111.8, 121.2, 128.2, 130.8, 131.4, 132.0, 132.1, 132.4, 136.1, 137.2, 139.0, 140.3, 141.0, 143.8, 145.6, 168.7, 177.2, 195.9; MS (EI): *m/z* (%) 557 (M^+^ + 1, 71.89), 556 (M^+^ + 1, 28.63), 555 (M^+^, 100.00). HRMS (EI): *m/z* calcd. for C_24_H_15_Cl_2_N_5_O_3_S_2_ (M^+^) 554.9988, found 554.9986.

#### (*Z*)-4-{3-Benzoyl-1-(4-nitrophenyl)-1*H*-thiazolo[4,5-*c*]pyridazin-6-ylideneamino}benzenesulfonamide (7 s)

Recrystallized from dioxane/DMF mixture (2:1), as pale orange crystal, yield: 2.45 g (92%), m.p. 249–250 °C; IR (KBr): 𝑣/cm^−1^ 1700 (CO); ^1^H-NMR (600 MHz, TFA-*d*): *δ* = 7.39–7.43 (m, 4 H, 2Ar-H and SO_2_*NH*_2_), 7.62 (d, *J* = 7.2 Hz, 2 H, Ar-H), 7.68 (d, *J* = 7.2 Hz, 2 H, Ar-H), 7.75 (t, *J* = 7.2 Hz, 2 H, Ar-H), 7.88 (d, *J* = 8.4 Hz, 2 H, Ar-H), 8.25–8.27 (m, 2 H, Ar-H and pyridine-*H4*), 8.34 (d, *J* = 8.4 Hz, 2 H, Ar-H); ^13^C{^1^H} NMR (150 MHz, TFA-*d*): *δ* = 111.4, 121.0, 128.0, 130.4, 130.9, 131.2, 131.3, 132.2, 136.3, 136.9, 137.6, 139.4, 140.1, 140.8, 145.4, 168.5, 177.0, 197.4; MS (EI): *m/z* (%) 533 (M^+^ + 1, 27.82), 532 (M^+^, 100.00). HRMS (EI): *m/z* calcd. for C_24_H_16_N_6_O_5_S_2_ (M^+^) 532.0618, found 532.0617.

#### (*Z*)-{6-[5-(1-Methyl-1*H*-indole-3-carbonyl)-2-phenyl-2*H*-[1,2,3]triazol-4-ylimino]-1-phenyl-1,6-dihydrothiazolo[4,5-*c*]pyridazin-3-yl}phenylmethanone (7t)

Recrystallized from EtOH/DMF mixture (1:2), as yellow crystal, yield: 2.75 g (88%), m.p. 292–293 °C; IR (KBr): 𝑣/cm^−1^ 1698, 1665 (2CO); ^1^H-NMR (600 MHz, TFA-*d*): *δ* = 4.01 (s, 3 H, C*H*_3_), 7.17 (t, *J* = 7.8 Hz, 1 H, Ar-H), 7.23 (d, *J* = 7.2 Hz, 2 H, Ar-H), 7.29 (t, *J* = 7.8 Hz, 2 H, Ar-H), 7.34–7.40 (m, 2 H, Ar-H), 7.49–7.51 (m, 3 H, Ar-H), 7.55–7.57 (m, 3 H, Ar-H), 7.68 (t, *J* = 7.2 Hz, 1 H, Ar-H), 7.85 (d, *J* = 7.2 Hz, 1 H, Ar-H), 8.13 (d, *J* = 7.8 Hz, 2 H, Ar-H), 8.19 (d, *J* = 7.8 Hz, 2 H, Ar-H), 8.95 (s, 1 H, in dole C-*H*), 9.19 (s, 1 H, pyridine-*H4*); ^13^C{^1^H} NMR (150 MHz, TFA-*d*): *δ* = 36.5 (*C*H_3_), 114.6, 115.21, 122.8, 124.9, 127.4, 128.2, 128.6, 129.3, 131.1, 131.7, 132.3, 132.8, 133.4, 133.6,  134.1, 135.5, 136.0, 137.9, 140.9, 141.4, 142.1, 142.4, 146.4, 149.8, 151.1, 157.7, 162.6, 162.9, 194.1; MS (EI): *m/z* (%) 633 (M^+^ + 1, 3.25), 632 (M^+^, 10.54). HRMS (EI): *m/z* calcd. for C_36_H_24_N_8_O_2_S (M^+^) 632.1737, found 632.1737.

#### (*Z*)-{1-(4-Chlorophenyl)-6-[5-(1-methyl-1*H*-indole-3-carbonyl)-2-phenyl-2*H*-[1,2,3]triazol-4-ylimino]-1,6-dihydrothiazolo[4,5-*c*]pyridazin-3-yl}phenylmethanone (7 u)

Recrystallized from EtOH/DMF mixture (1:3), as yellow crystal, yield: 2.95 g (89%), m.p. 295–296 °C; IR (KBr): 𝑣/cm^−1^ 1699, 1662 (2CO); ^1^H-NMR (600 MHz, TFA-*d*): *δ* = 4.08 (s, 3 H, C*H*_3_), 7.21–7.26 (m, 3 H, Ar-H), 7.29 (d, *J* = 8.4 Hz, 2 H, Ar-H), 7.45 (d, *J* = 7.8 Hz, 1 H, Ar-H), 7.55–7.58 (m, 3 H, Ar-H), 7.61–7.64 (m, 3 H, Ar-H), 7.75 (t, *J* = 7.8 Hz, 1 H, Ar-H), 7.89 (d, *J* = 7.8 Hz, 1 H, Ar-H), 8.18 (d, *J* = 8.4 Hz, 2 H, Ar-H), 8.25–8.27 (m, 2 H, Ar-H), 8.99 (s, 1 H, pyridine-*H4*), 9.27 (s, 1 H, indole C-*H*); ^13^C{^1^H} NMR (150 MHz, TFA-*d*): *δ* = 36.3 (*C*H_3_), 114.5, 115.0, 122.7, 124.7, 128.0, 128.4, 128.6, 129.1, 131.0, 131.7, 132.2, 133.4, 134.0, 135.4, 135.8, 137.8, 139.2, 140.66, 140.74, 141.2, 142.0, 146.3, 149.7, 151.1, 157.6, 162.1, 162.6, 171.0, 193.8; MS (EI): *m/z* (%) 668 (M^+^ + 2, 3.45), 667 (M^+^ + 1, 4.67), 666 (M^+^, 7.41). HRMS (EI): *m/z* calcd. for C_36_H_23_ClN_8_O_2_S (M^+^) 666.1348, found 666.1344.

### MTT *in vitro* cytotoxic assay

The *in vitro* cytotoxic activities of **7a-u** were determined toward three human cancer cell lines including HCT-116 (colon cancer), MCF-7 (breast cancer) and A549 (lung cancer), and one normal cell MCF10A (breast cell), by employing the MTT colorimetric assay. The cell lines were grown in a MEM (Minimum Essential Media) medium boosted with 10% fetal bovine serum (FBS), penicillin, and streptomycin at 37 °C in 5% CO_2_ humidified atmosphere. The tested compounds were dissolved in DMSO and the resulting solutions were diluted by adding culture medium to the required concentrations. Each cell line was distributed into 96 well sterilized plates in a medium boosted with 10% FBS then treated with the three concentrations of the tested compounds and doxorubicin. In all cases, the incubation time was 48 h in 5% CO_2_ incubator at 37 °C. Then the medium was removed and a solution of MTT was added and the mixture was incubated again for 4 h. After incubation, the MTT solution was removed and the obtained purple formazan dye was dissolved in DMSO. Finally the absorbance at 490 nm was measured utilizing a microplate spectrophotometer and triplicate experiments were carried out. The IC_50_ values (Table 3) were calculated using the GraphPad Prism Scientific graphing explore software version 7.0.

### Supporting Information

- ^1^H NMR, ^13^C{^1^H} NMR, MS, and HRMS spectra for the reported compounds (PDF).
- Crystal data for compound **7 m** (CIF).

## Supplementary information

Supplementary Information.

Supplementary Information 2.
