# Author Correction: The first Q-Tube based high-pressure synthesis of anti-cancer active thiazolo[4,5-c]pyridazines via the [4  +  2] cyclocondensation of 3-oxo-2-arylhydrazonopropanals with 4-thiazolidinones

**DOI:** 10.1038/s41598-020-66549-x

**Published:** 2020-06-17

**Authors:** Hamada Mohamed Ibrahim, Haider Behbehani

**Affiliations:** 1 Chemistry Department, Faculty of Science, Kuwait University, P.O. Box 5969, Safat, 13060 Kuwait; 2 Chemistry Department, Faculty of Science, Fayoum University, P.O. Box, 63514, Fayoum, Egypt

Correction to: *Scientific Reports* 10.1038/s41598-020-63453-2, published online 16 April 2020

This Article contains errors in Table 2. The chemical structure for 4-thiazolidinone 4a-c is incorrect, the R substituent should be X. Additionally, in the ‘Entry’ column, number 18 appears twice. As a result, entries ‘18, 18, 19 and 20’ should read ‘18, 19, 20 and 21’, respectively. Finally, in the ‘Product’ column, the labels for products 7e-7u were omitted.

The corrected Table 2 appears below as Table 1.

**Table 1:** Q-tube [4 + 2] cyclocondensation reactions of 4-thiazolidinone **4a-c** and arylhydrazonal **5**.


Entry	Reactants	Ar_1_	Ar_2_	X	Product	Yield (%)^a^
**1**	**4a** + **5a**	C_6_H_4_	C_6_H_5_			**98**
**2**	**4a** + **5b**	C_6_H_4_	4-Cl-C_6_H_4_			**99**
**3**	**4a** + **5c**	C_6_H_5_	4-NO_2_-C_6_H_4_			**94**
**4**	**4a** + **5d**	C_6_H_5_	4-MeO-C_6_H_4_			**95**
**5**	**4a** + **5e**	4-Cl-C_6_H_4_	4-Cl-C_6_H_4_			**96**
**6**	**4a** + **5 f**	4-Cl-C_6_H_4_	2-Cl-5-NO_2_-Ph			**98**
**7**	**4a** + **5 g**	4-Br-C_6_H_4_	2-Cl-5-NO_2_-Ph			**97**
**8**	**4a** + **5 h**	4-Br-C_6_H_4_	2,4-diF-C_6_H_3_			**94**
**9**	**4a** + **5i**	4-F-C_6_H_4_	4-Me-C_6_H_4_			**92**
**10**	**4a** + **5j**	C_10_H_7_	4-Cl-C_6_H_4_			**96**
**11**	**4a** + **5k**	C_10_H_7_	4-NO_2_-C_6_H_4_			**95**
**12**	**4a** + **5 l**	C_10_H_7_	4-NO_2_-C_6_H_4_			**93**
**13**	**4a** + **5 m**	C_4_H_3_S	C_6_H_5_			**92**
**14**	**4a** + **5n**	C_4_H_3_S	4-Cl-C_6_H_4_			**93**
**15**	**4a** + **5o**	C_4_H_3_O	2-Cl-5-NO_2_-Ph			**90**
**16**	**4a** + **5p**	*N*-Me-Ind	4-NO_2_-C_6_H_4_			**91**
**17**	**4b** + **5a**	C_6_H_5_	C_6_H_5_			**91**
**18**	**4b** + **5e**	4-Cl-C_6_H_4_	4-Cl-C_6_H_4_			**94**
**19**	**4b** + **5c**	C_6_H_5_	4-NO_2_-C_6_H_4_			**92**
**20**	**4c** + **5a**	C_6_H_5_	C_6_H_5_			**88**
**21**	**4c** + **5b**	C_6_H_5_	4-Cl-C_6_H_4_			**89**

^a^Reaction conditions: a mixture of 4-thiazolidinone **4a-c** (5 mmol), arylhydrazonal **5** (5 mmol), anhydrous sodium acetate (10 mmol) and glacial acetic acid (10 mL) was charged in the glass tube of the Q-tube reactor and heated in an oil bath at 170 °C for 25 min.

