# Supplementary material for: The first Q-Tube based high-pressure synthesis of anti-cancer active thiazolo[4,5-c]pyridazines via the [4 + 2] cyclocondensation of 3-oxo-2-arylhydrazonopropanals with 4-thiazolidinones
Source: Sci Rep. 2020 Apr 16;10:6492. doi: 10.1038/s41598-020-63453-2 (PMC7162912; doi:10.1038/s41598-020-63453-2)
Supplement: Supplementary file 1 — Supplementary Information. [file 41598_2020_63453_MOESM1_ESM.pdf]

Supporting Information

For

The first Q-Tube based high-pressure synthesis of anti-cancer active thiazolo[4,5-*c*]pyridazines *via* the [4+2] cyclocondensation of 3-oxo-2-arylhydrazonopropanals with 4-thiazolidinones

Hamada Mohamed Ibrahim,[*,1,2] and Haider Behbehani,[*,1]

[1] Chemistry Department, Faculty of Science, Kuwait University, P.O. Box 5969, Safat 13060, Kuwait.

[2] Chemistry Department, Faculty of Science, Fayoum University, P.O. Box 63514, Fayoum, Egypt.

[E-Mail: hmi00@fayoum.edu.eg, hamadaaldeb@yahoo.com, Tel.: +965-55216585, Fax: +965-248-164-82]

ORCID iD: 0000-0001-8355-2676

[E-Mail: drhaider.b@gmail.com, Tel.: +965-55888646, Fax: +965-248-164-82]

ORCID iD: 0000-0002-1859-5191

Contents

1- Copies of ^{1}H, ^{13}C NMR, MS and HRMS spectra for the reported compounds. S2

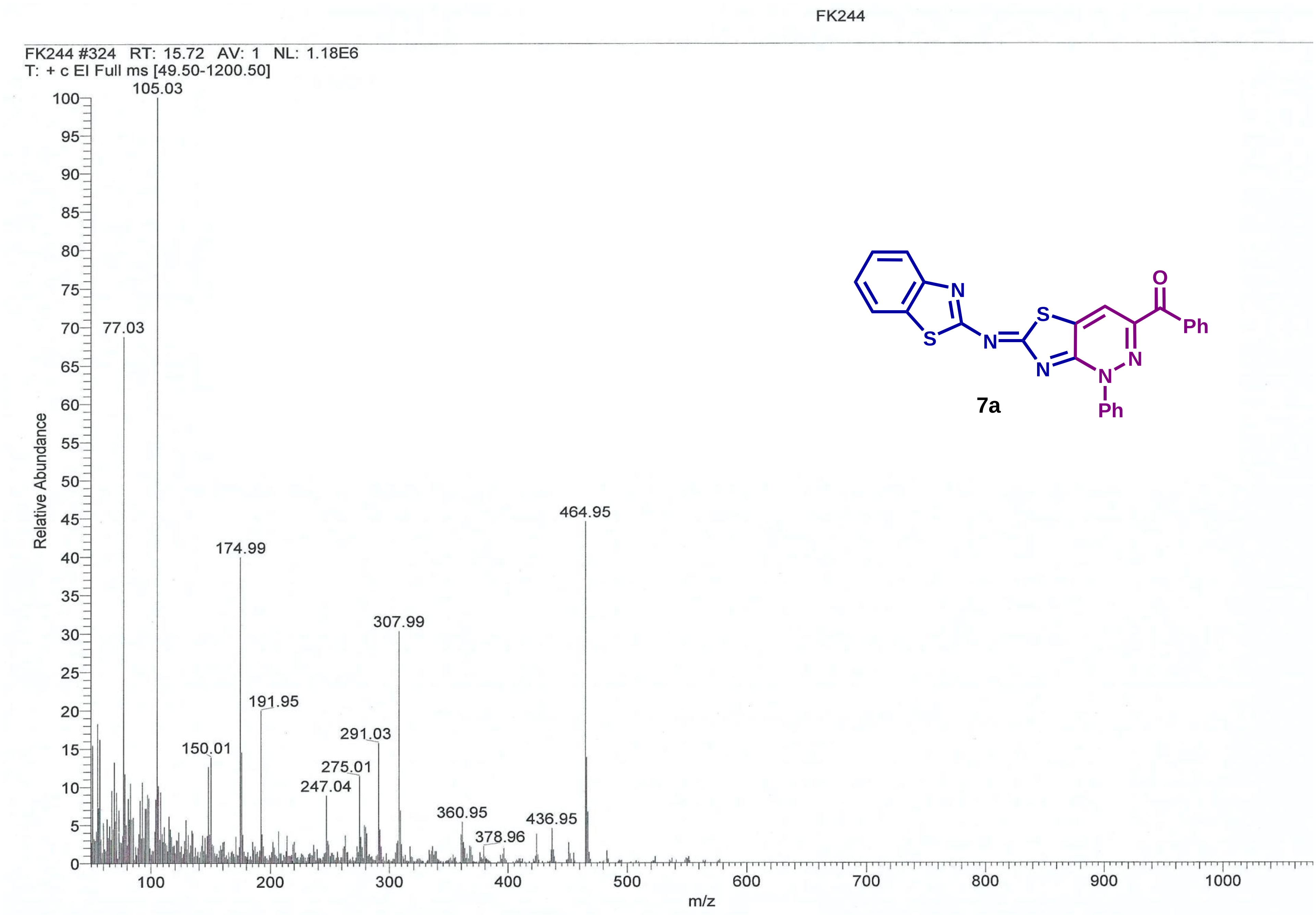

Figure S1. Mass Spectra for compound **7a**.

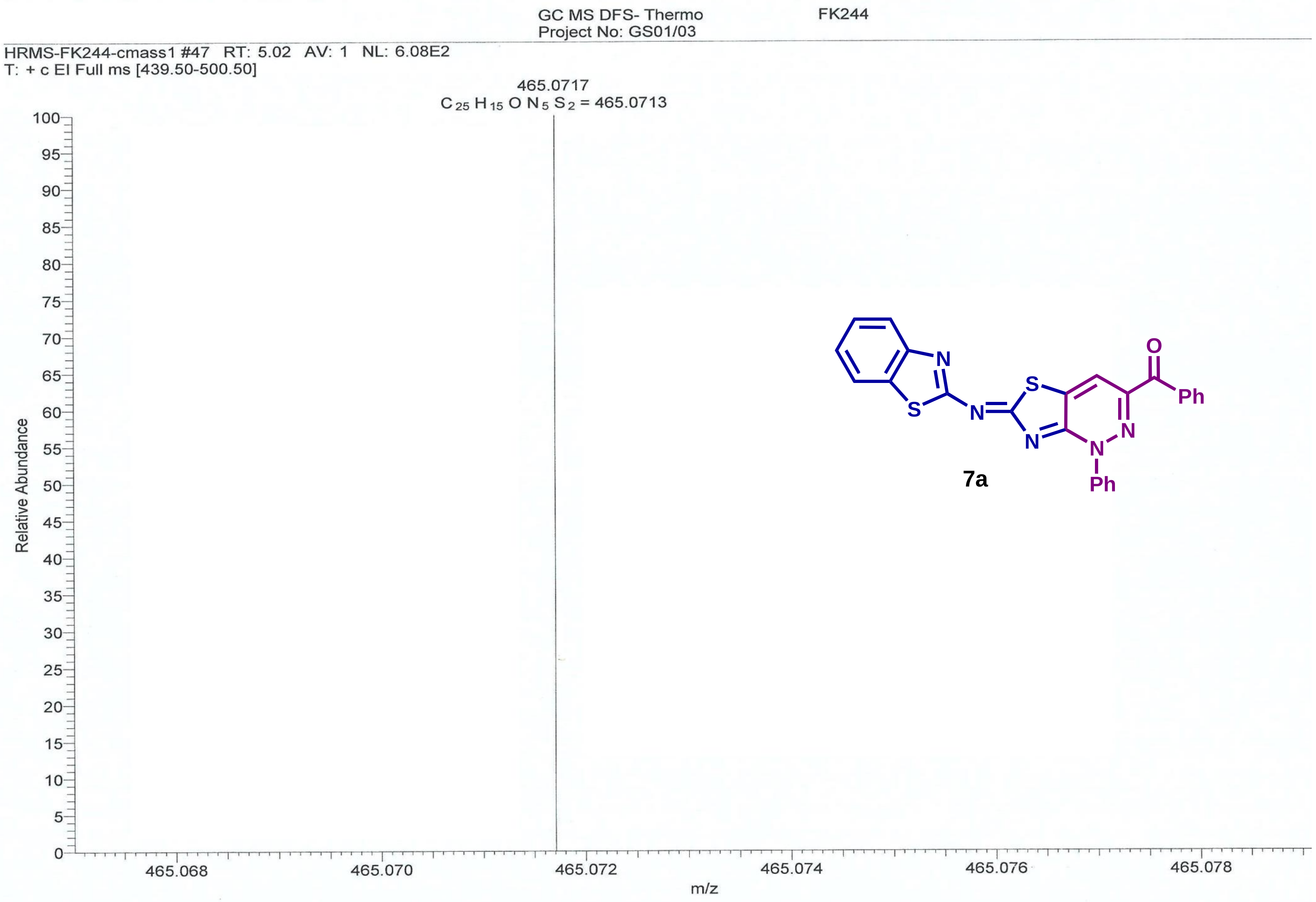

Figure S2. HRMS Spectra for compound **7a**.

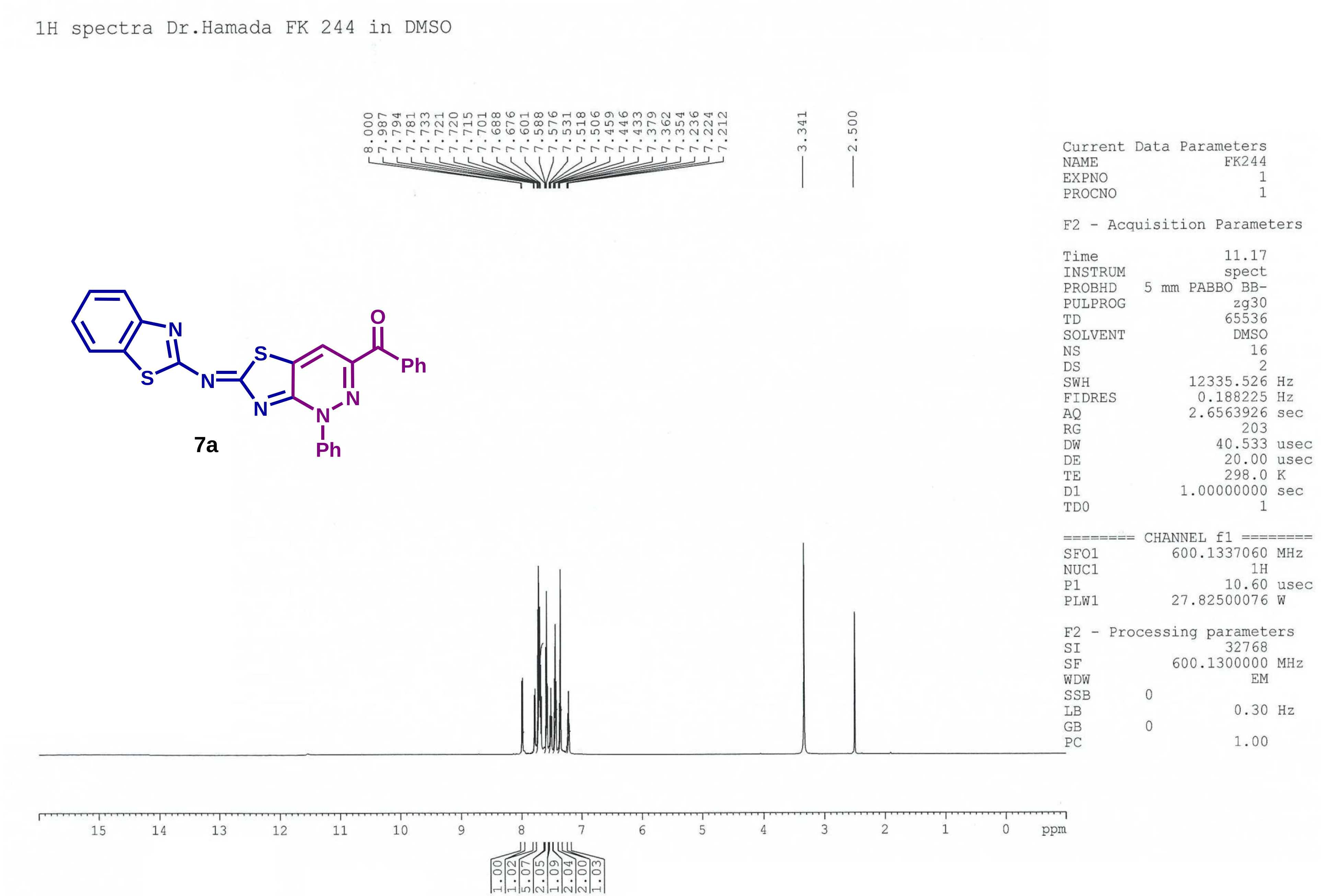

Figure S3. ^{1}H NMR Spectra (DMSO-d_6, 600 MHz) for compound **7a**.

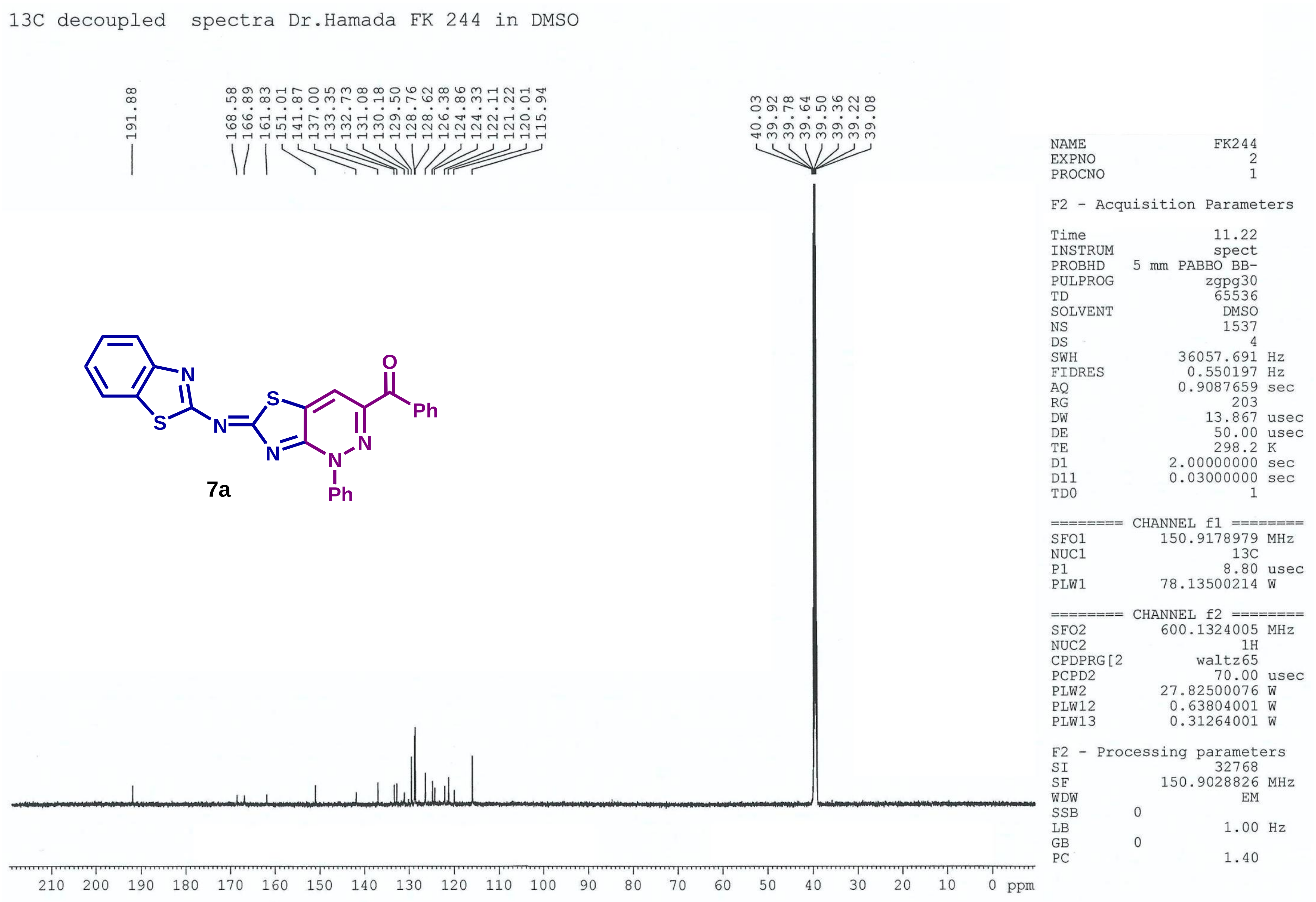

Figure S4. ^{13}C NMR Spectra (DMSO-d_6, 150 MHz) for compound **7a**.

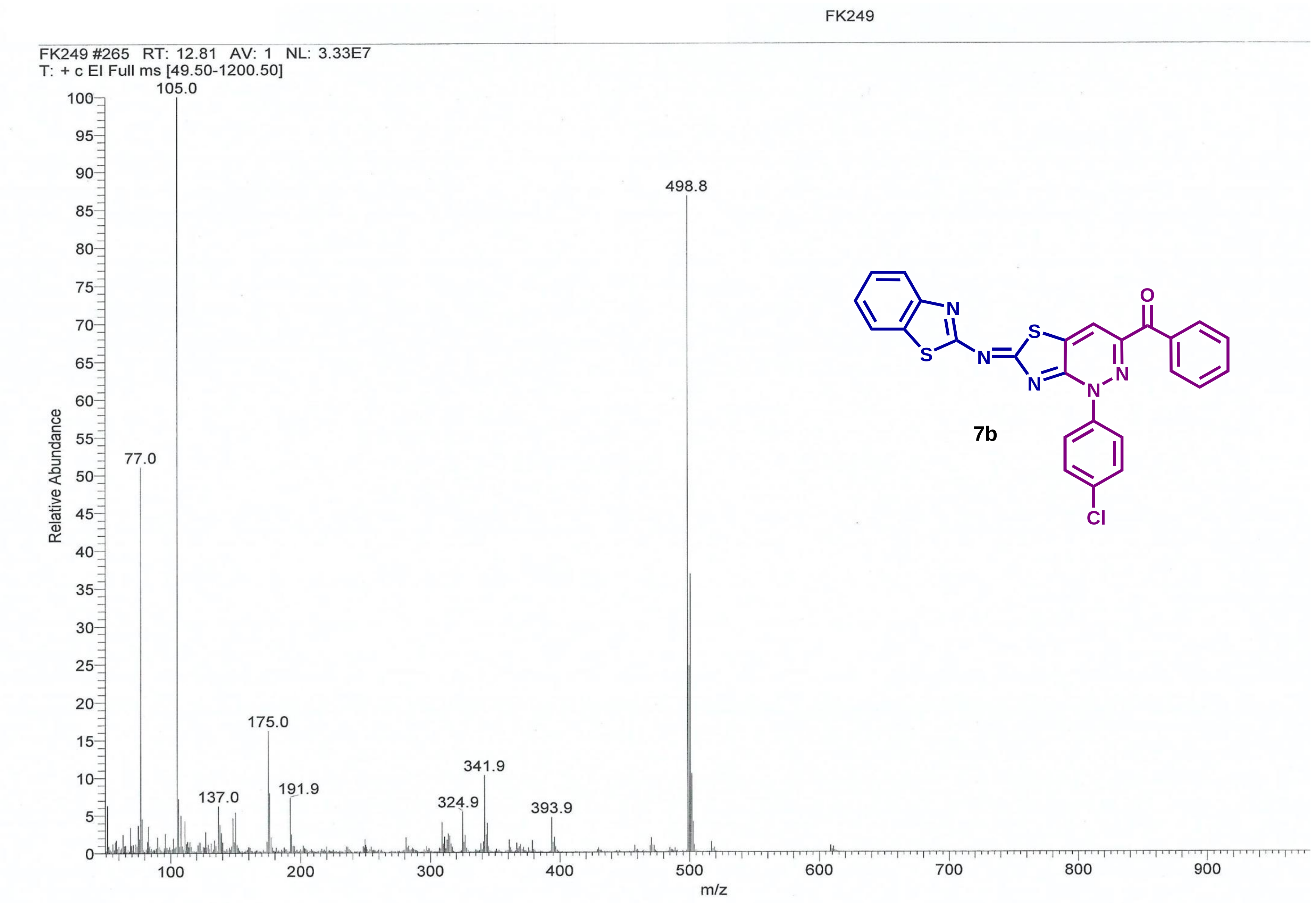

Figure S5. Mass Spectra for compound **7b**.

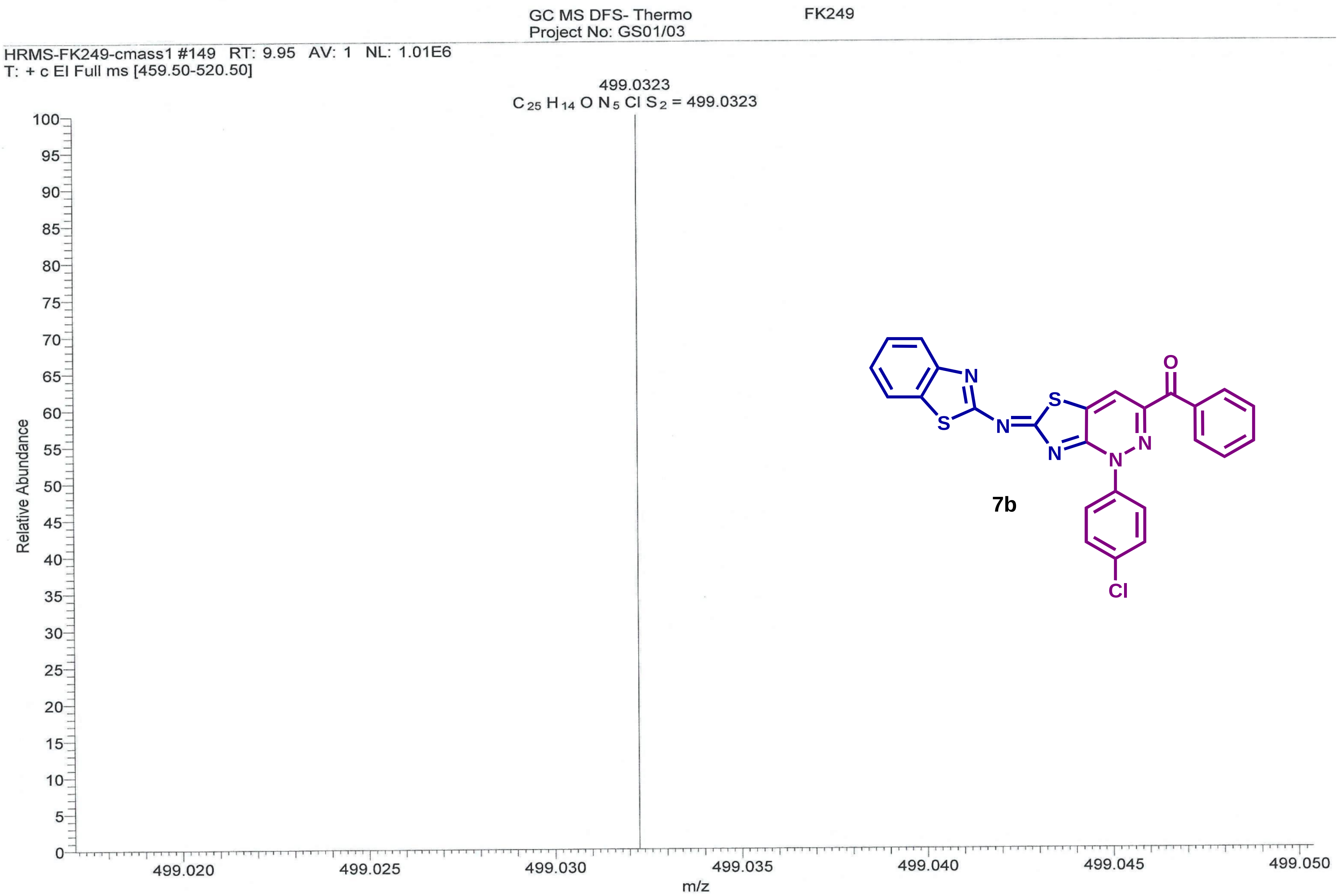

Figure S6. HRMS Spectra for compound **7b**.

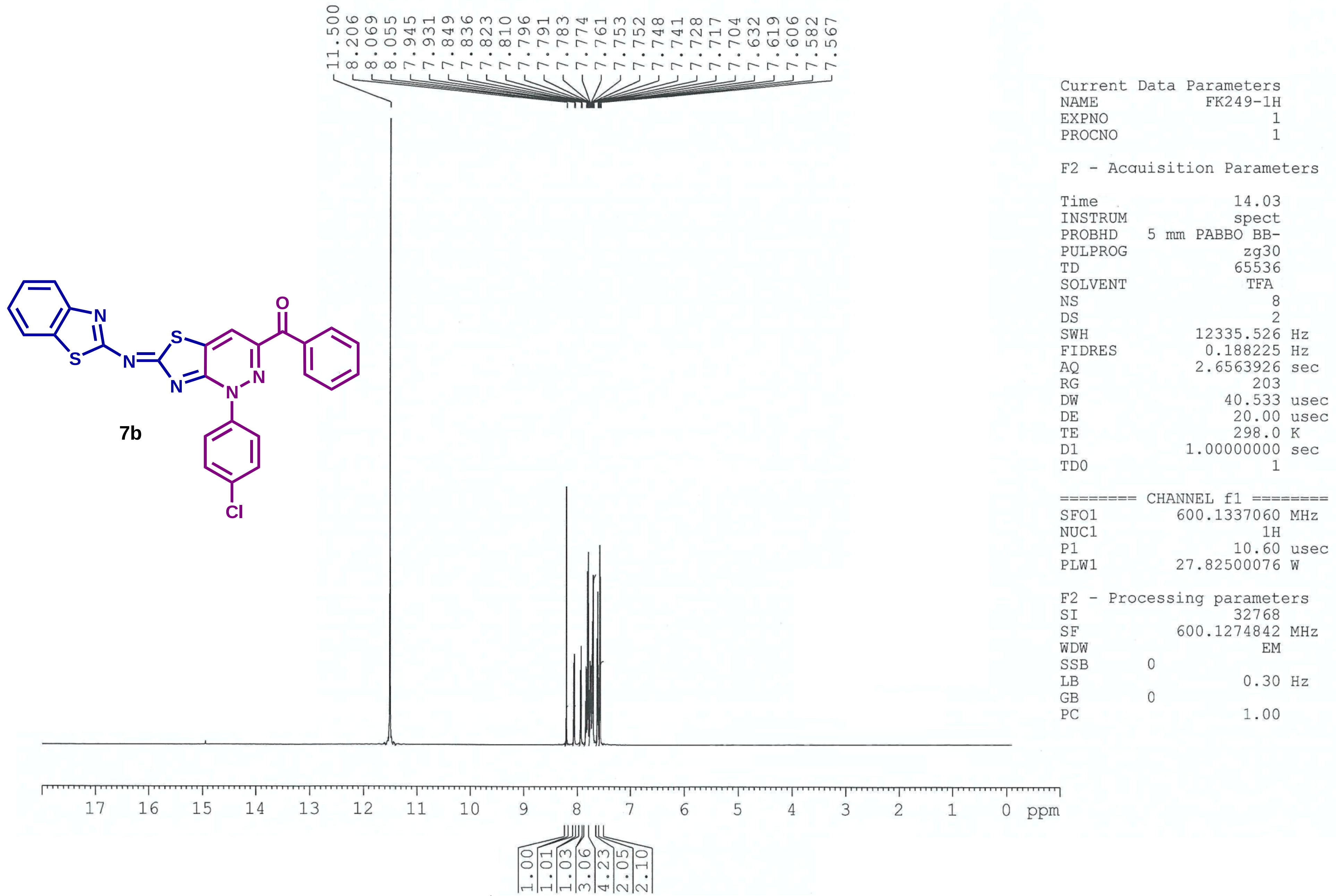

Figure S7. ^{1}H NMR Spectra (600 MHz, TFA-*d*) for compound **7b**.

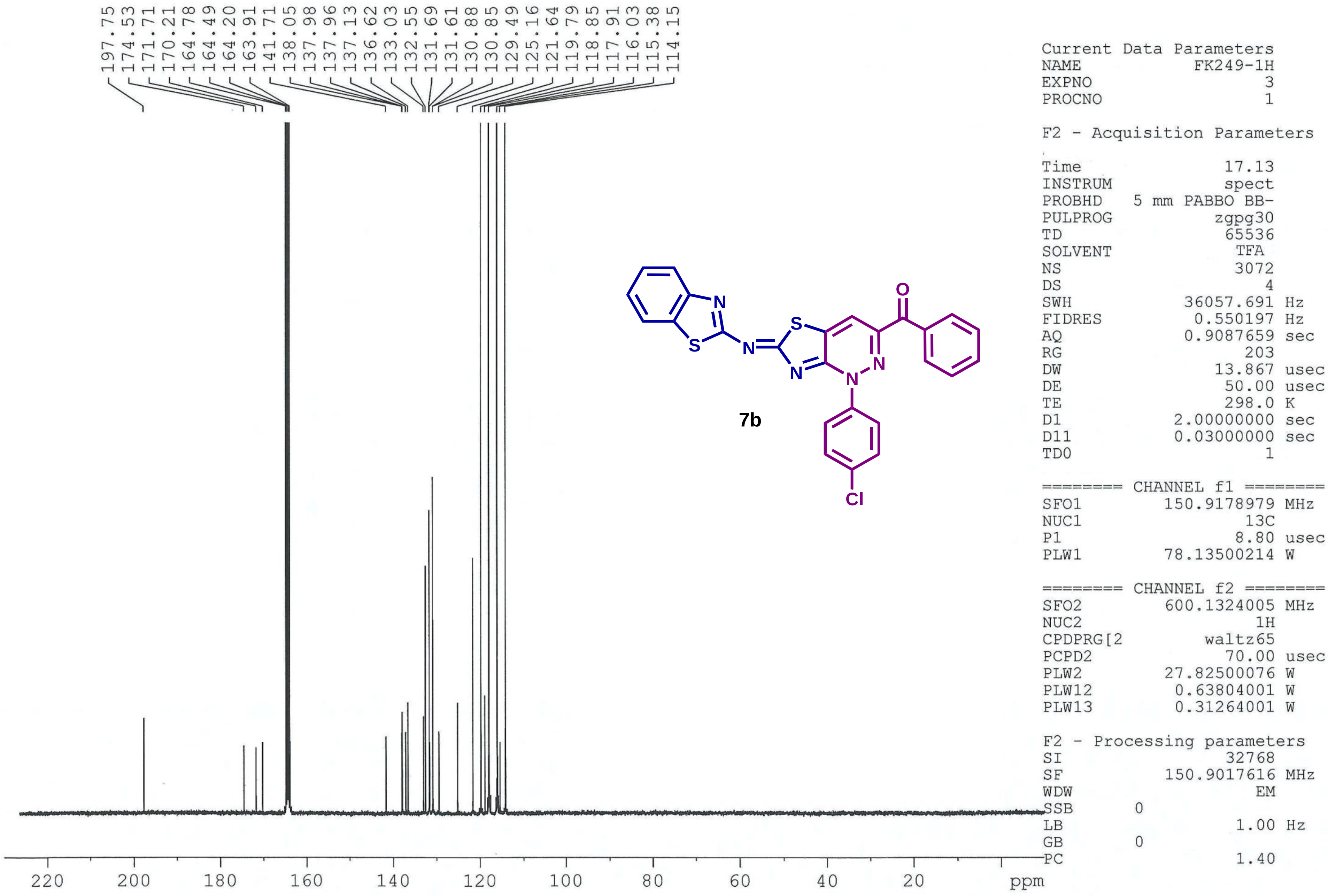

7b

Figure S8. ^{13}C NMR Spectra (TFA-*d*, 150 MHz) for compound **7b**.

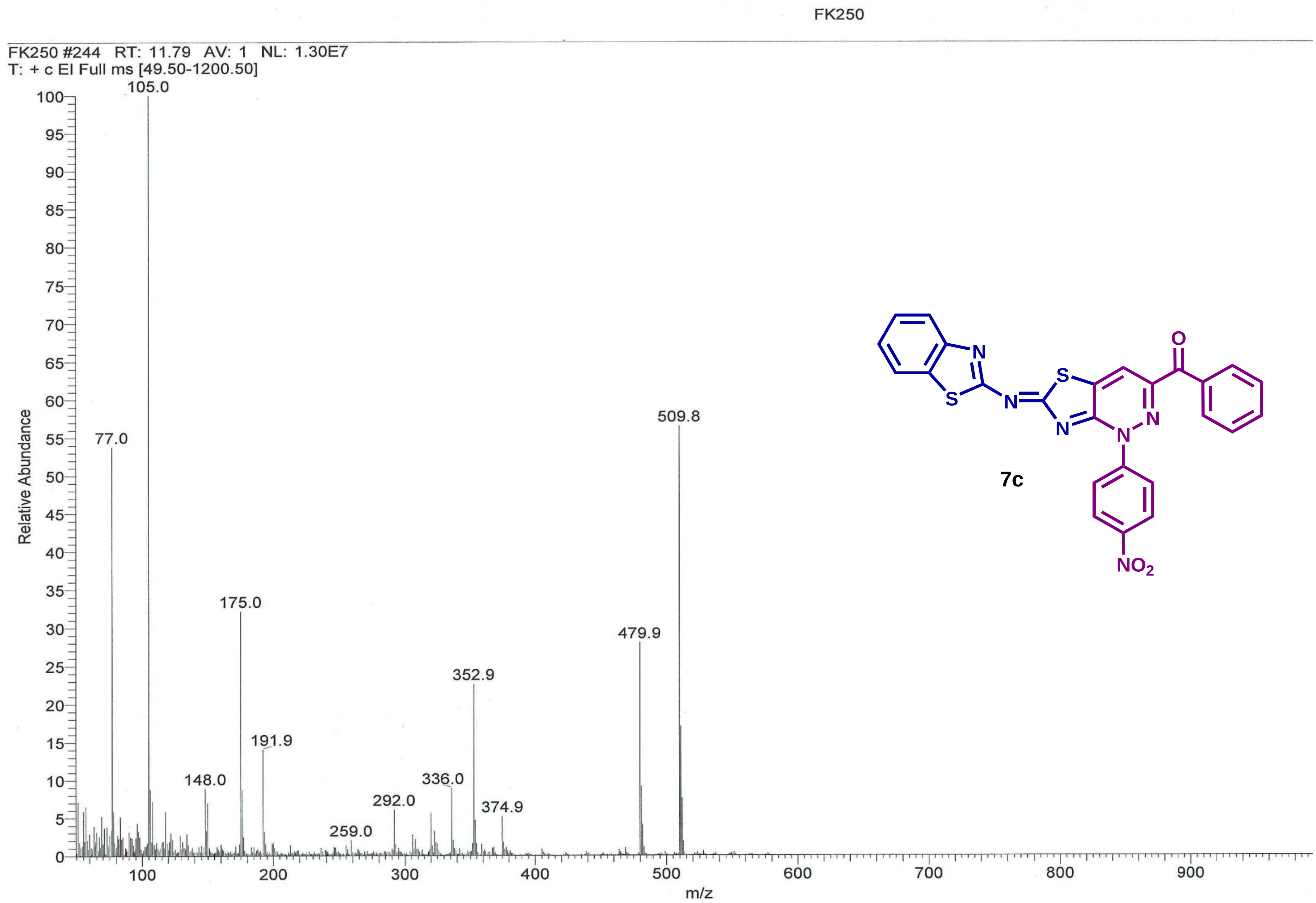

Figure S9. Mass Spectra for compound **7c**.

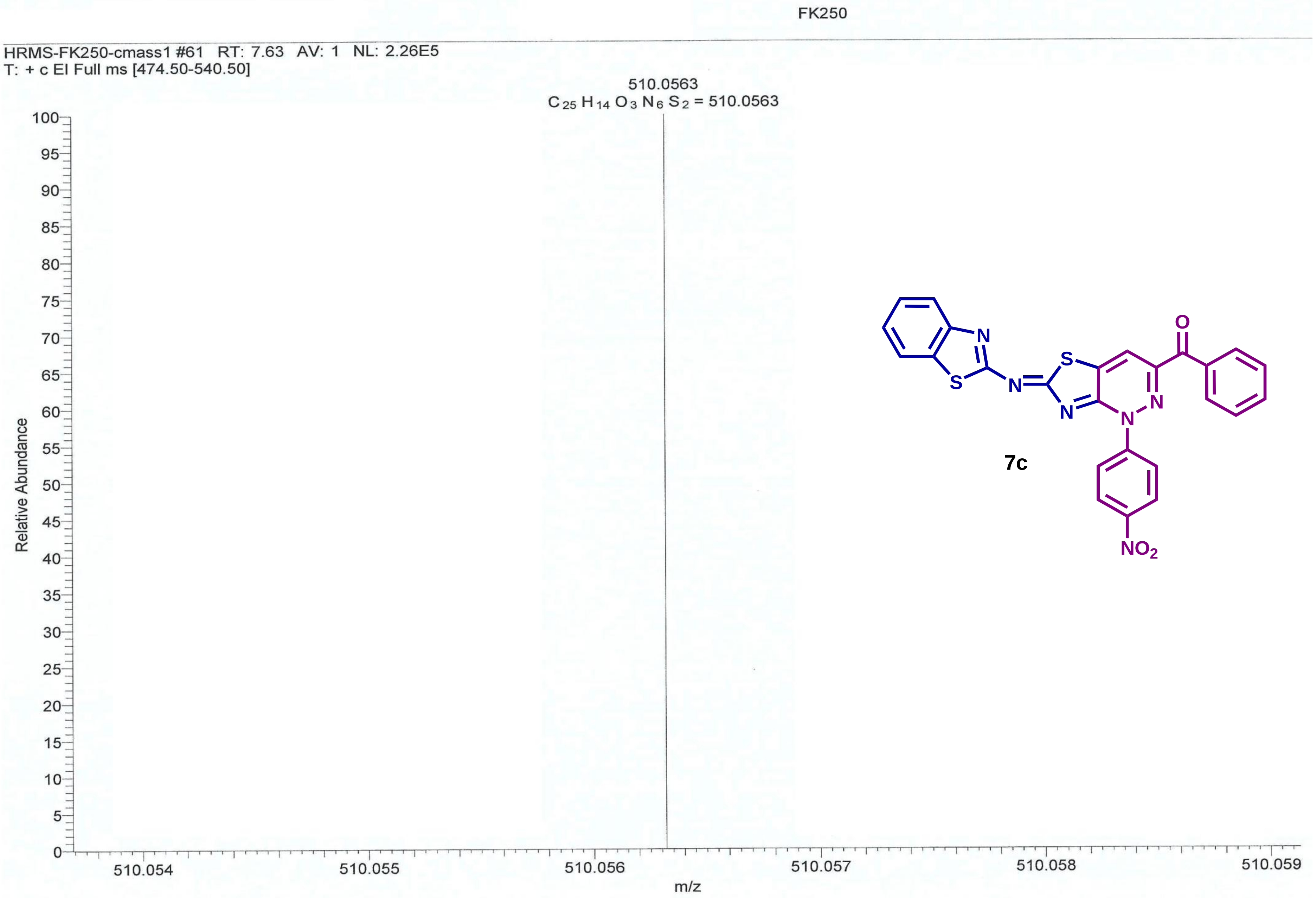

7c

Figure S10. HRMS Spectra for compound **7c**.

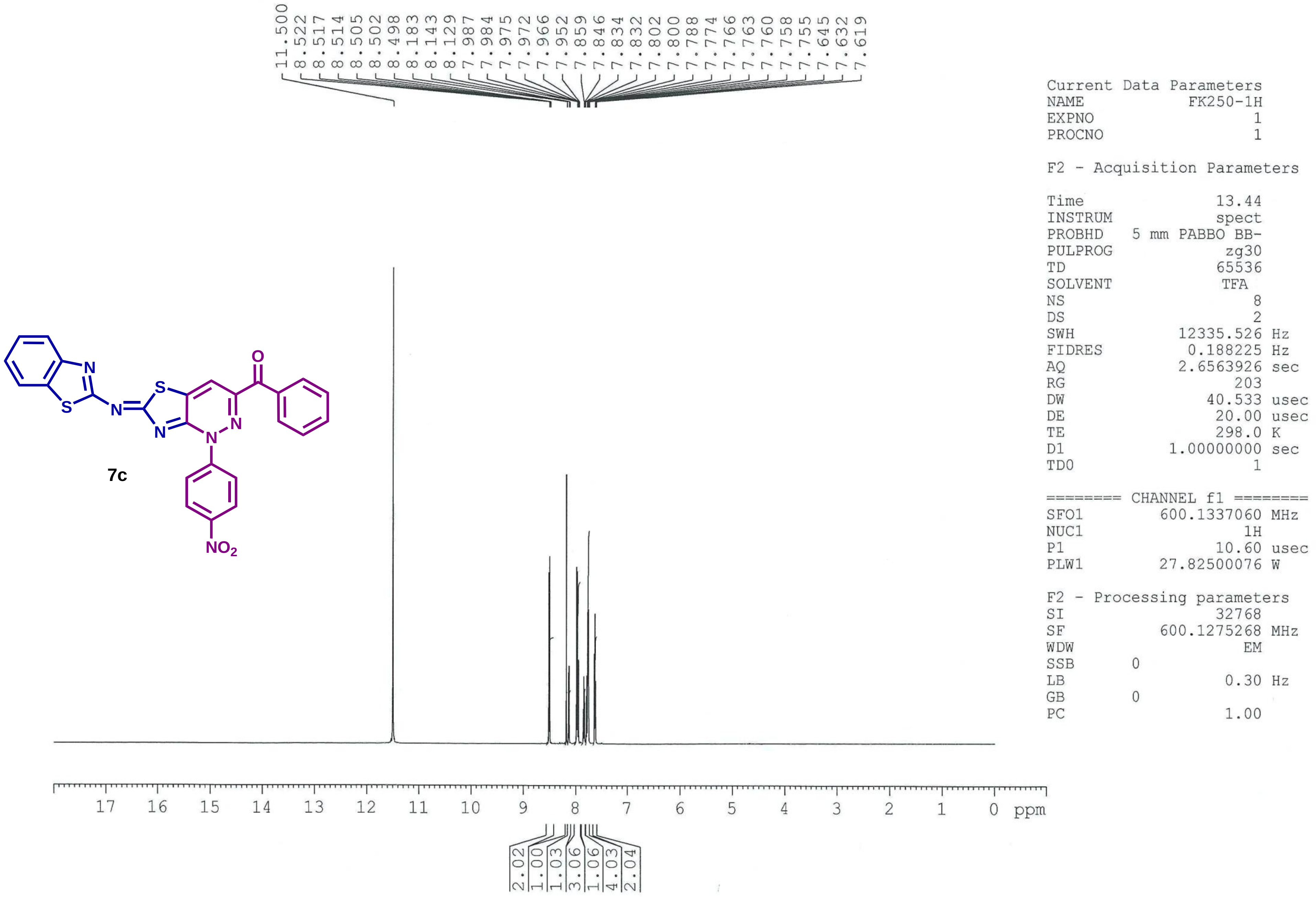

Figure S11. ^{1}H NMR Spectra (600 MHz, TFA-*d*) for compound **7c**.

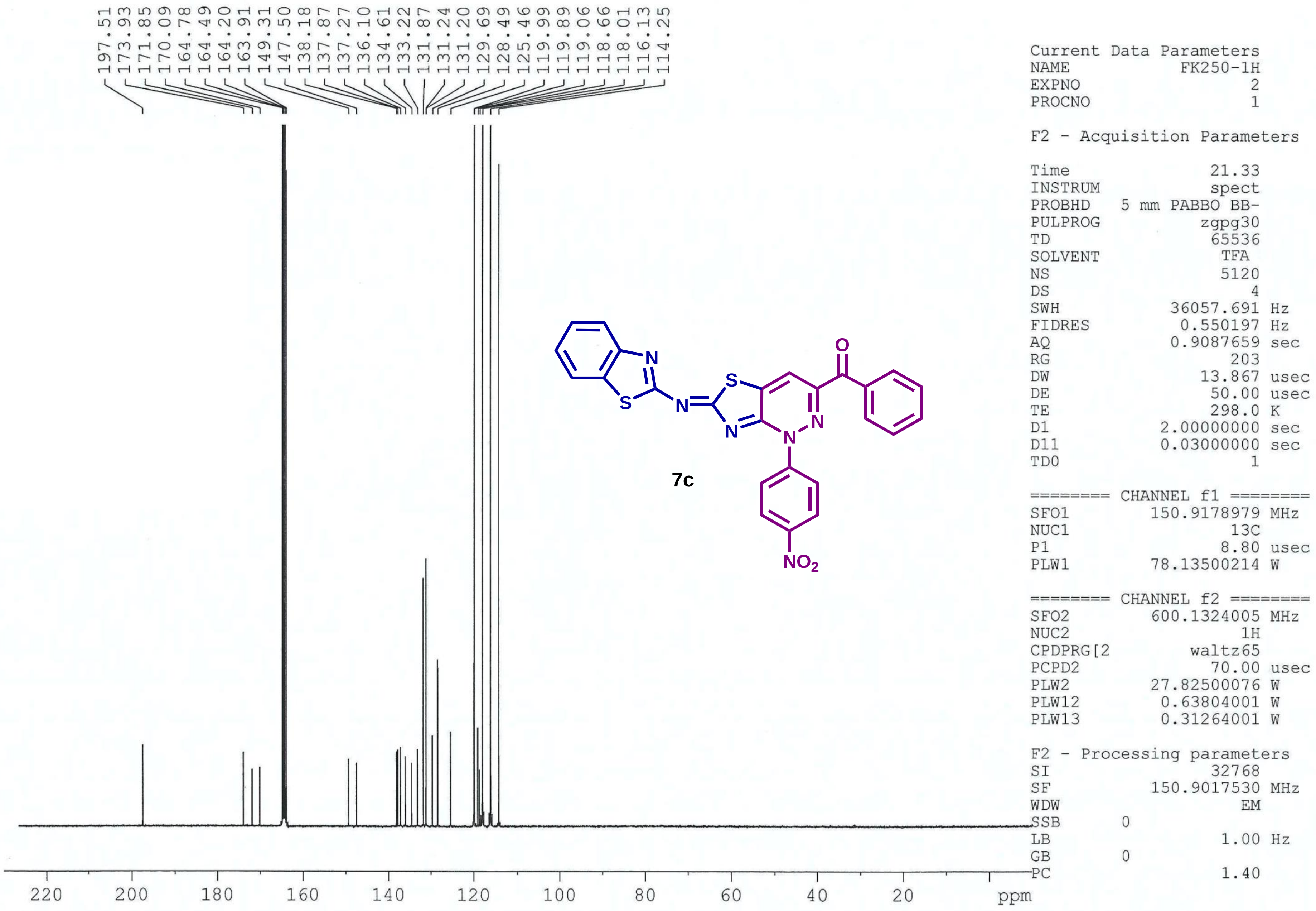

Figure S12. ^{13}C NMR Spectra (TFA-*d*, 150 MHz) for compound **7c**.

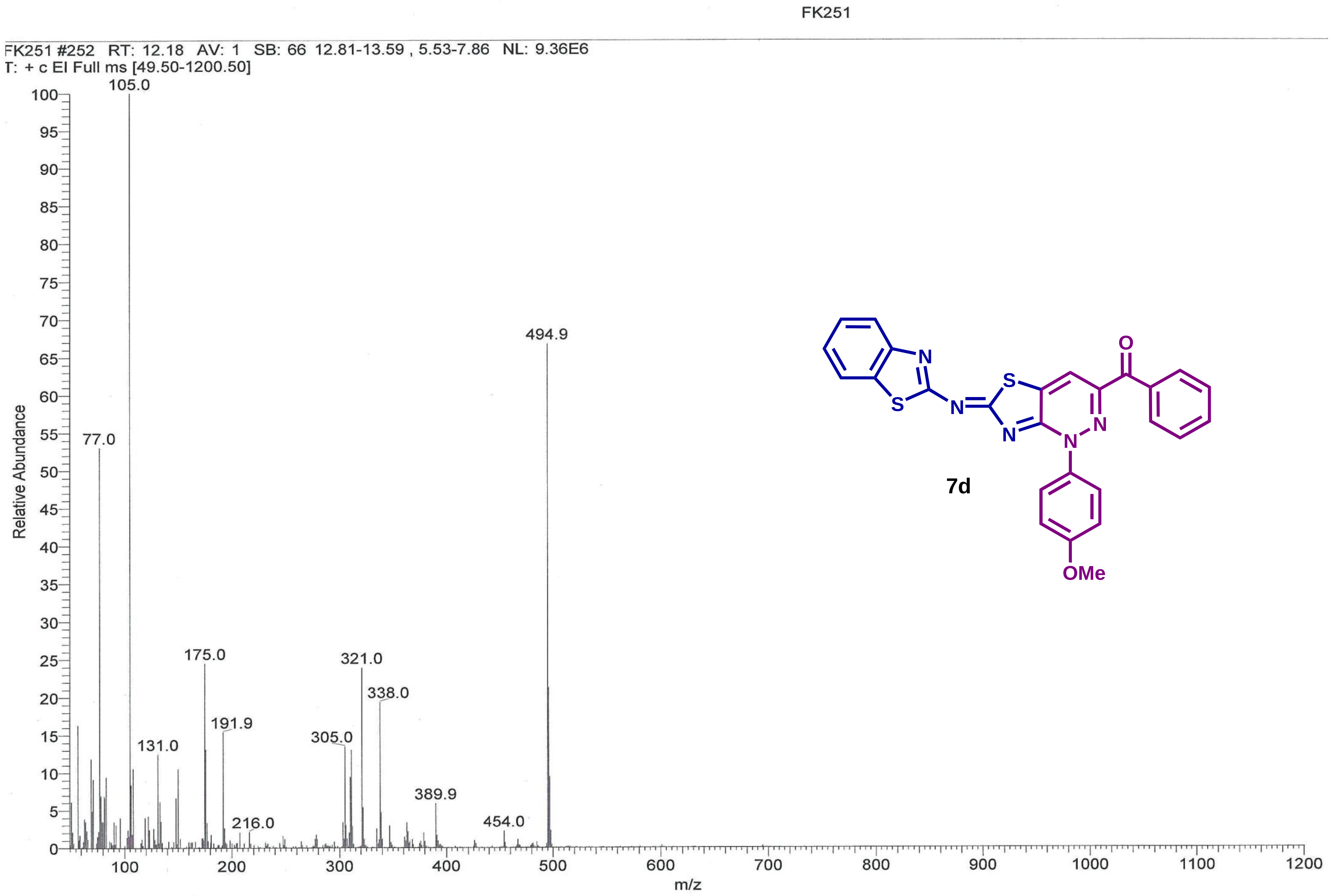

Figure S13. Mass Spectra for compound **7d**.

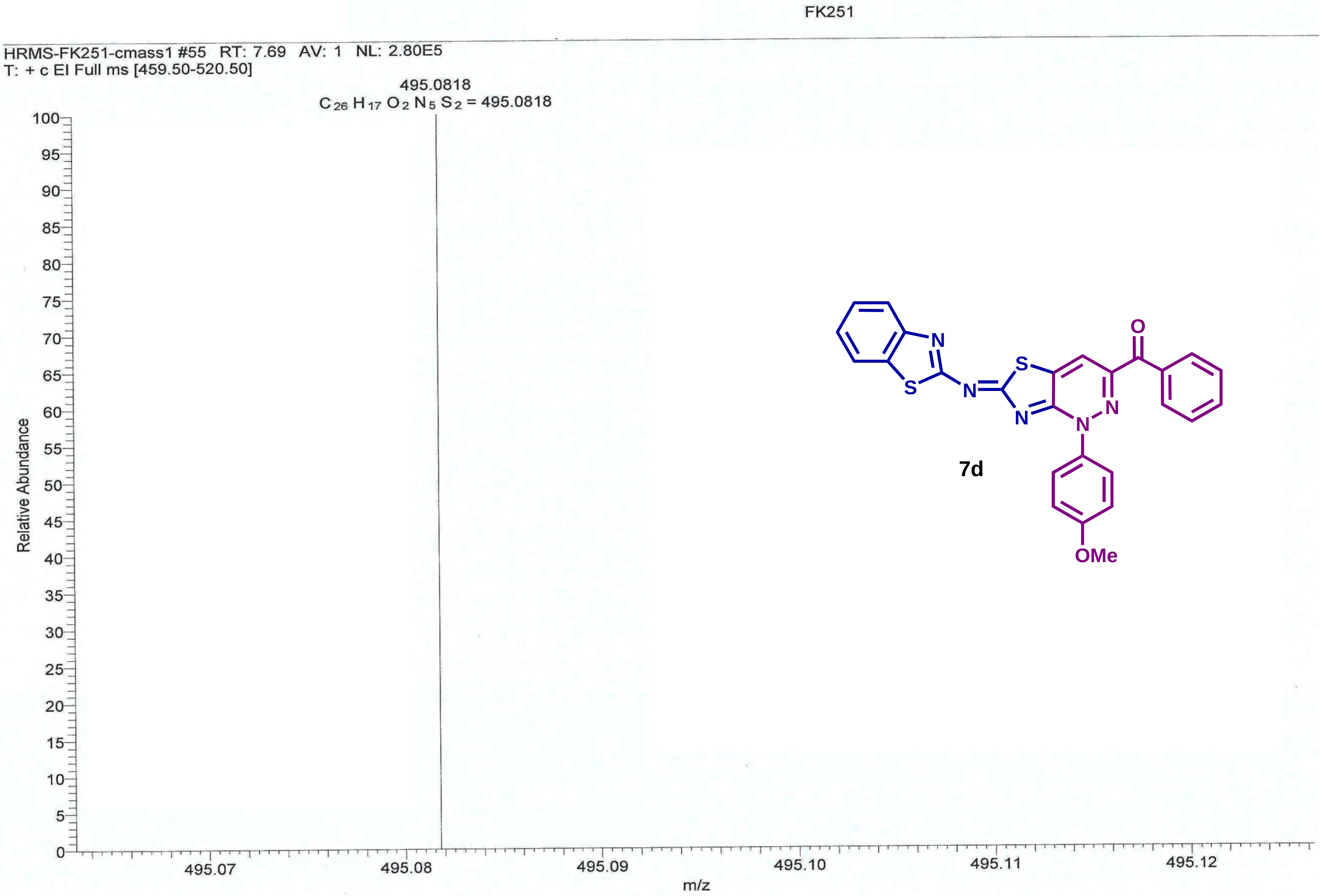

Figure S14. HRMS Spectra for compound **7d**.

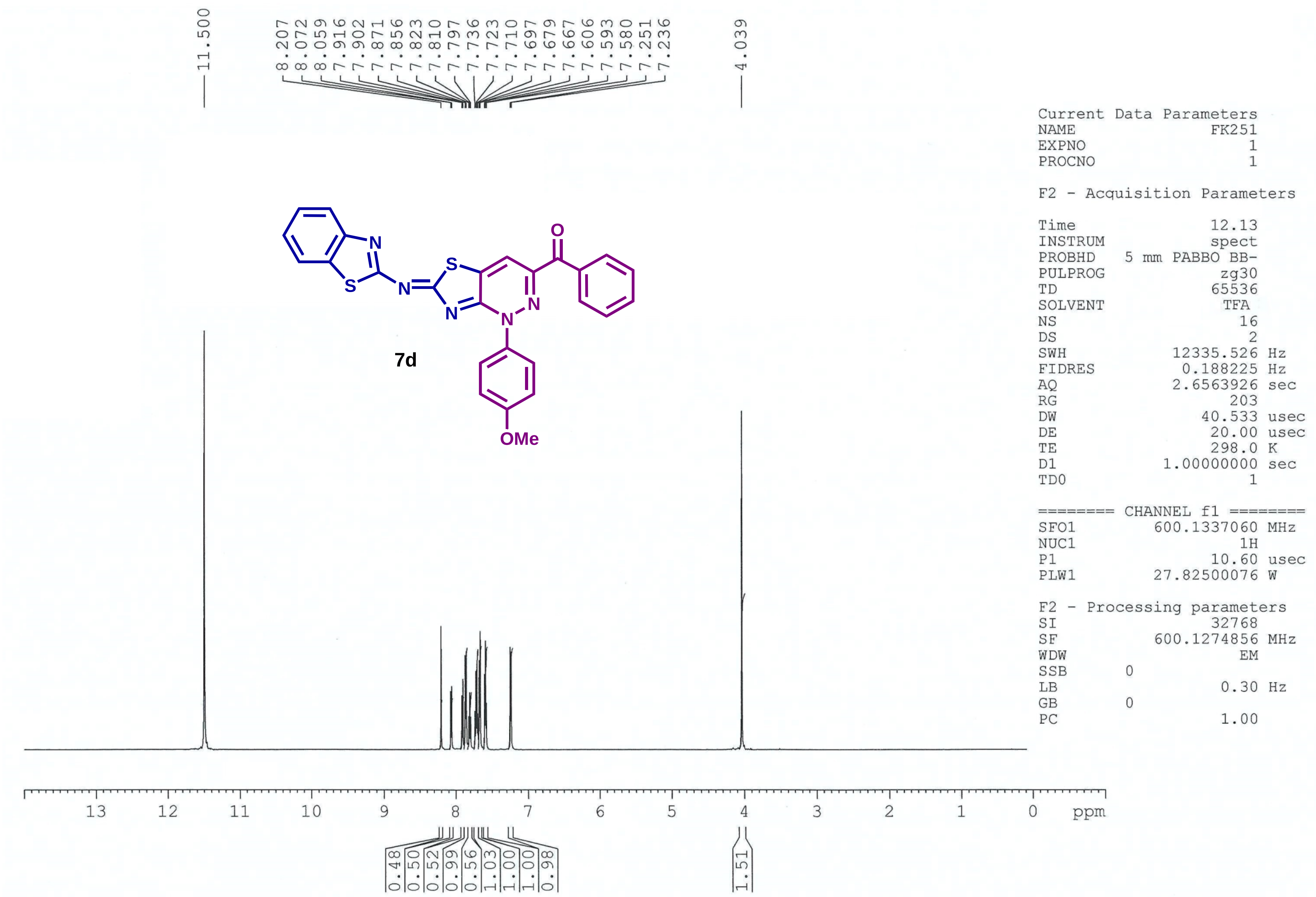

Figure S15. ^{1}H NMR Spectra (TFA-*d*, 600 MHz) for compound **7d.**

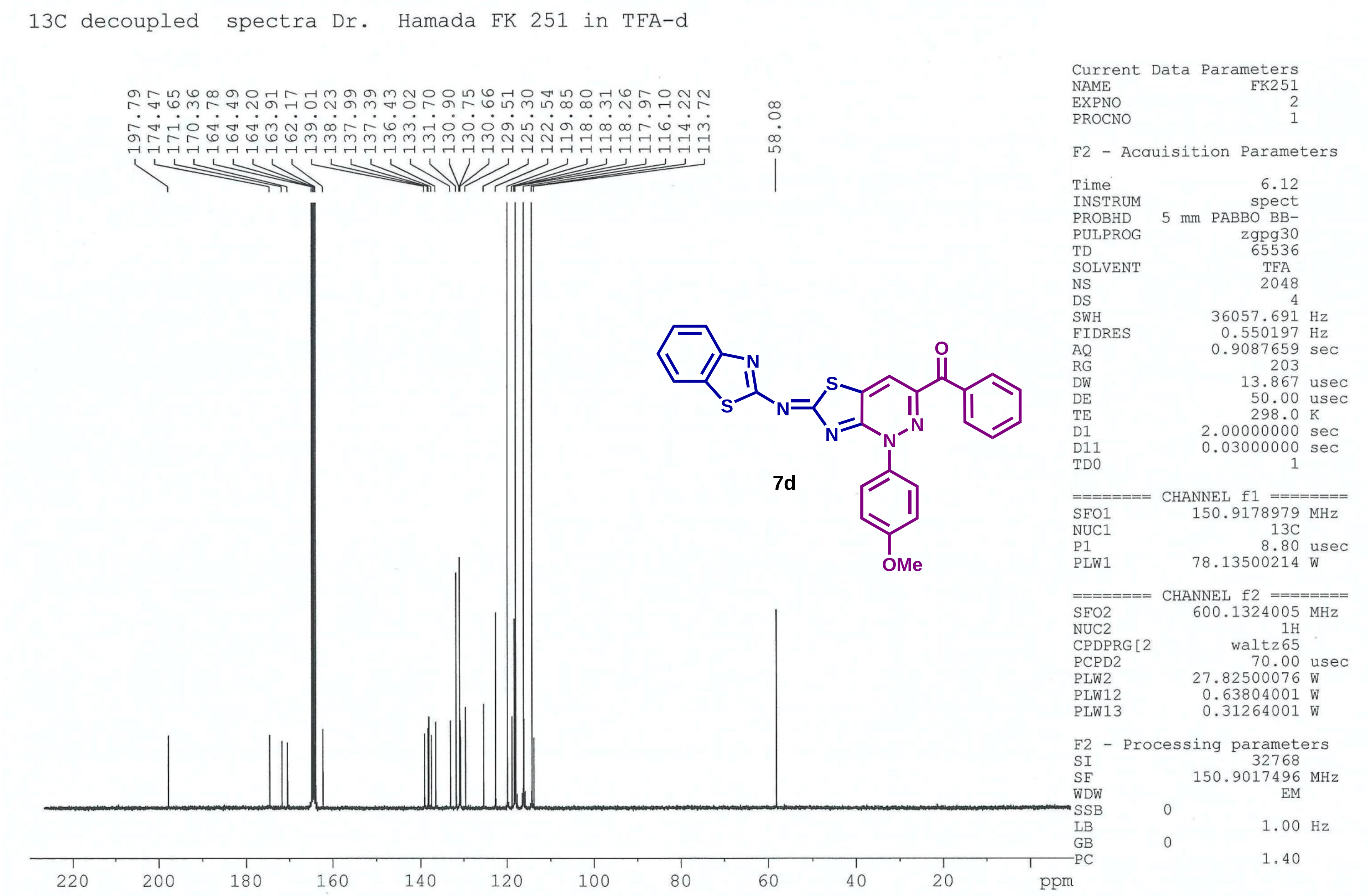

Figure S16. ^{13}C NMR Spectra (TFA-*d*, 150 MHz) for compound **7d.**

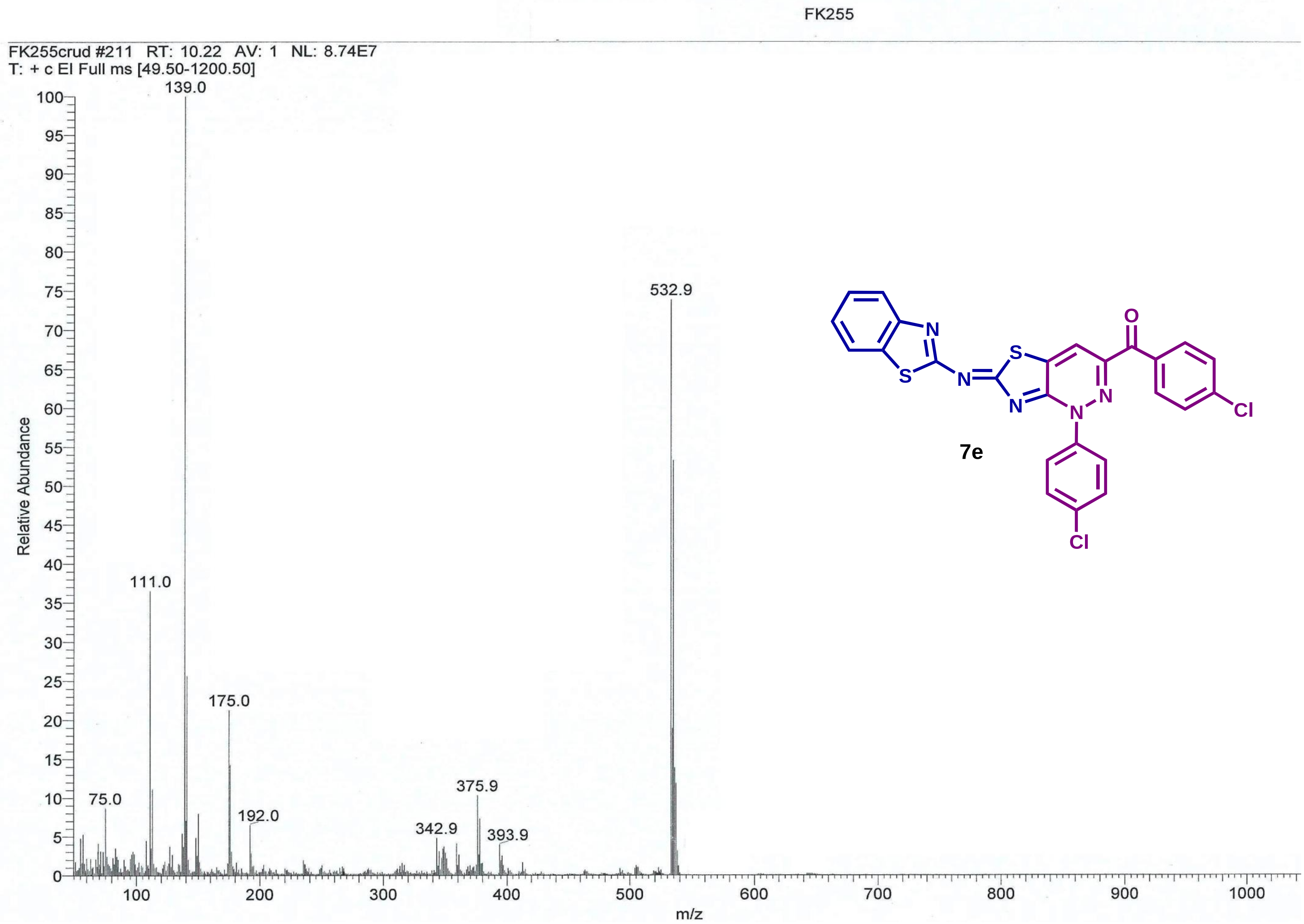

Figure S17. Mass Spectra for compound **7e**.

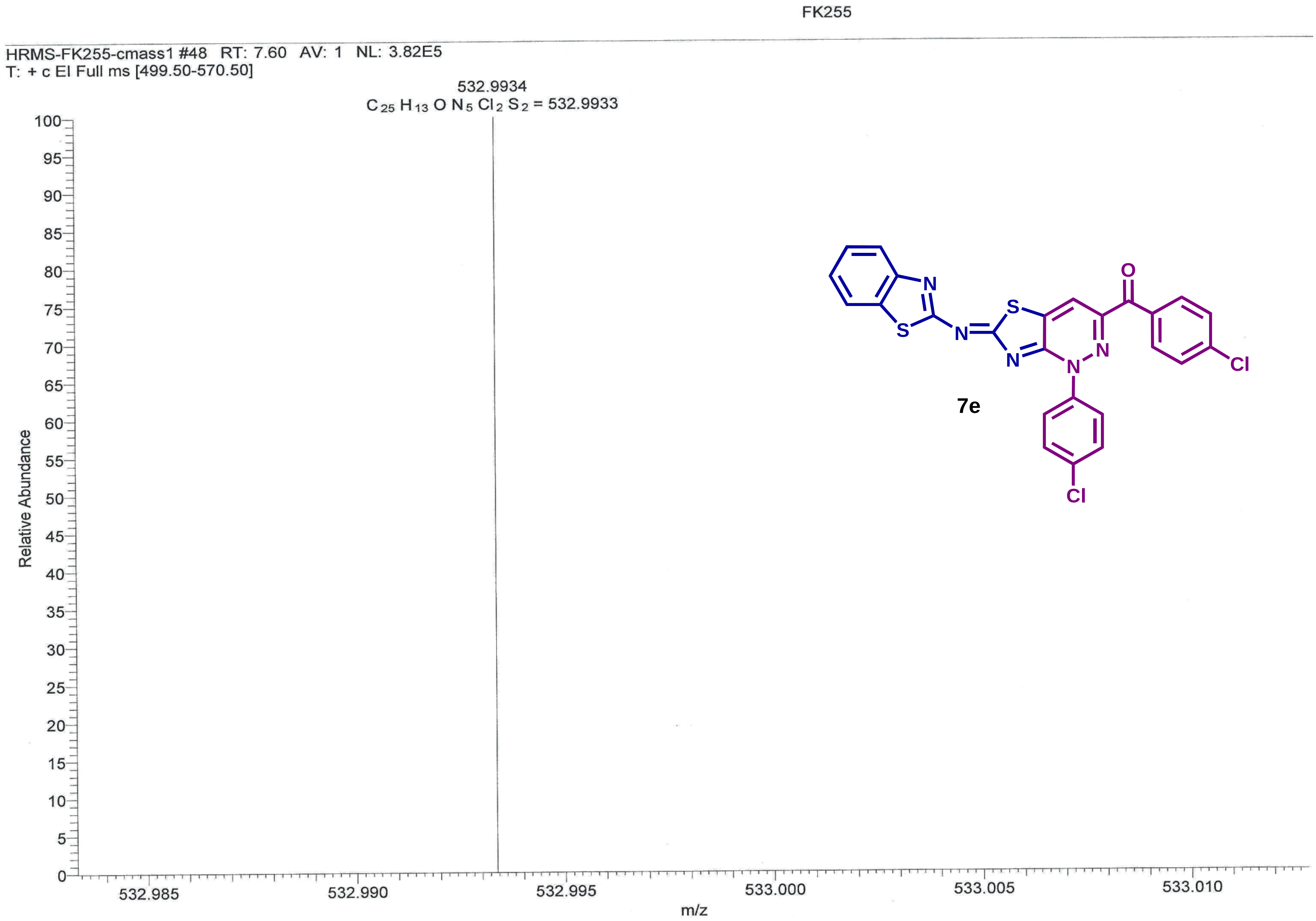

Figure S18. HRMS Spectra for compound **7e**.

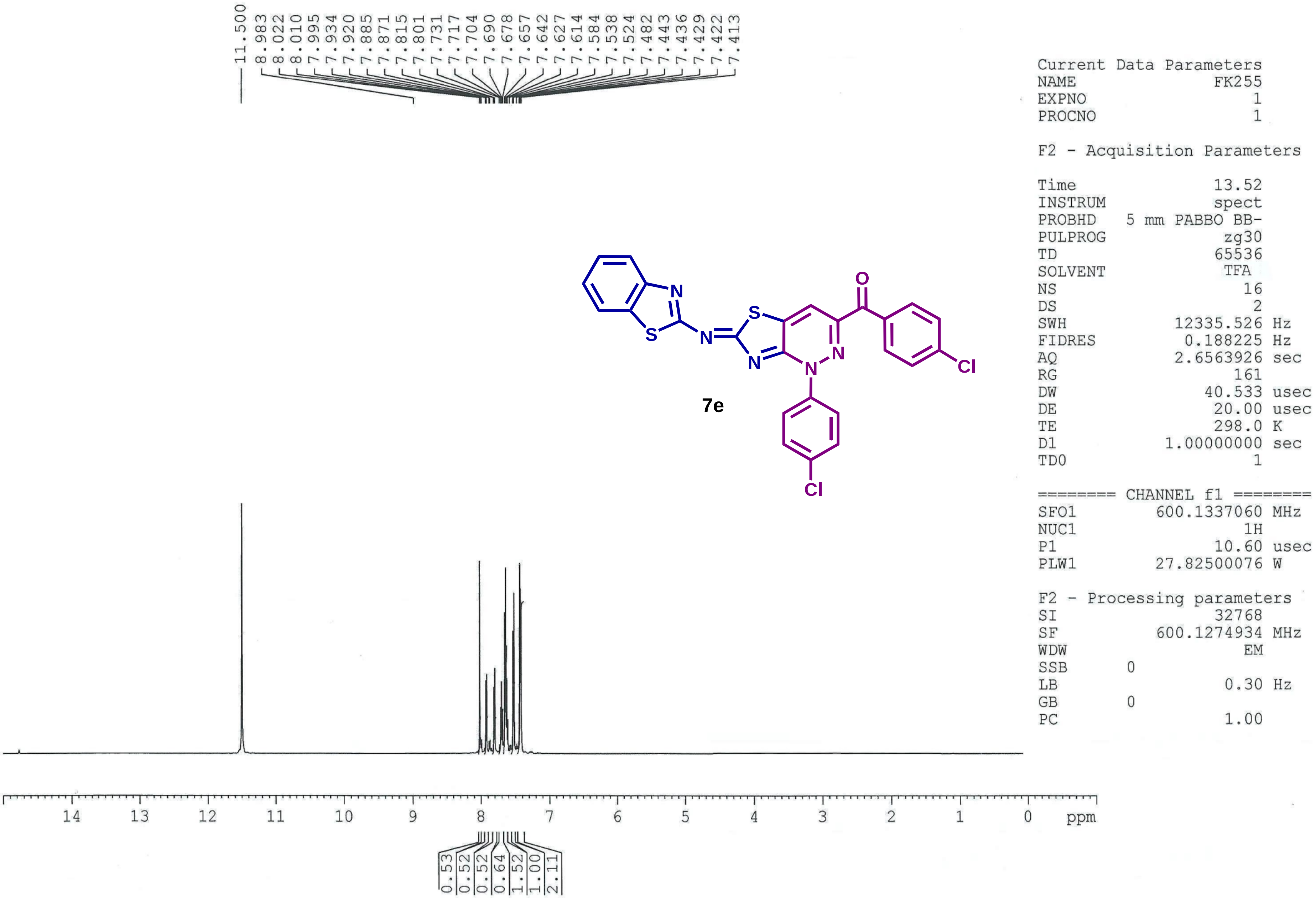

Figure S19. ^{1}H NMR Spectra (TFA-*d*, 600 MHz) for compound **7e**.

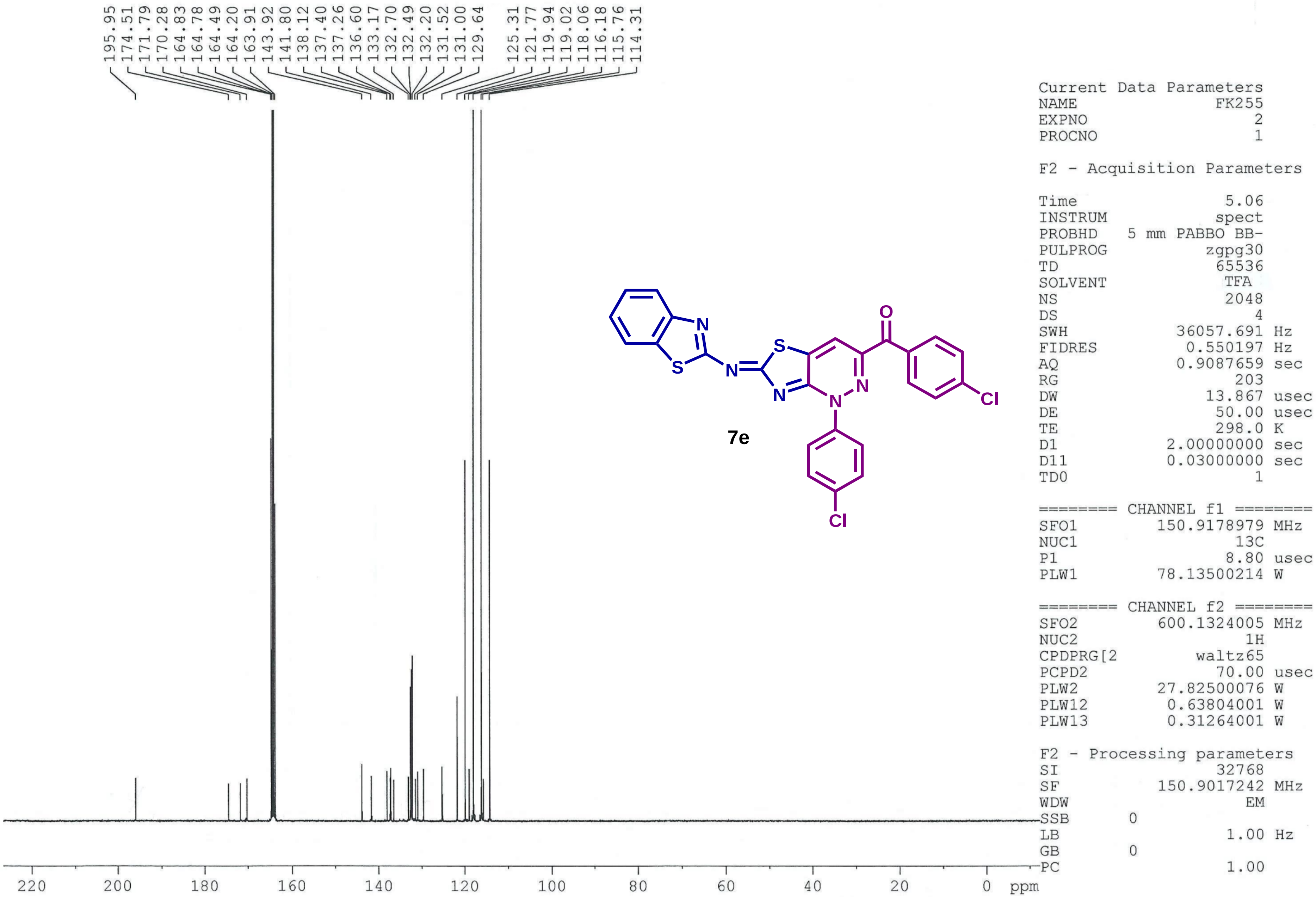

Figure S20. ^{13}C NMR Spectra (TFA-*d*, 150 MHz) for compound **7e**.

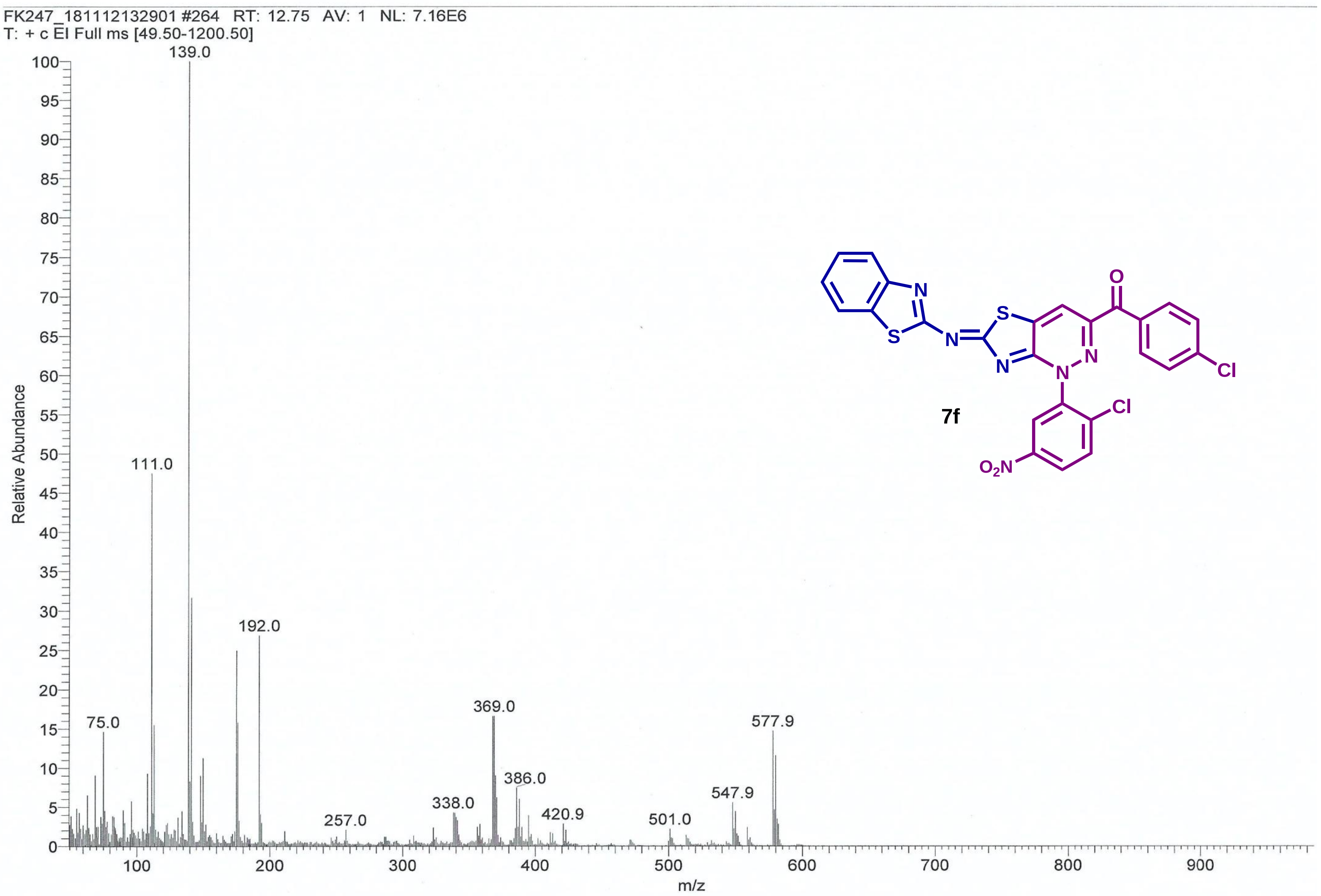

Figure S21. Mass Spectra for compound 7**f**.

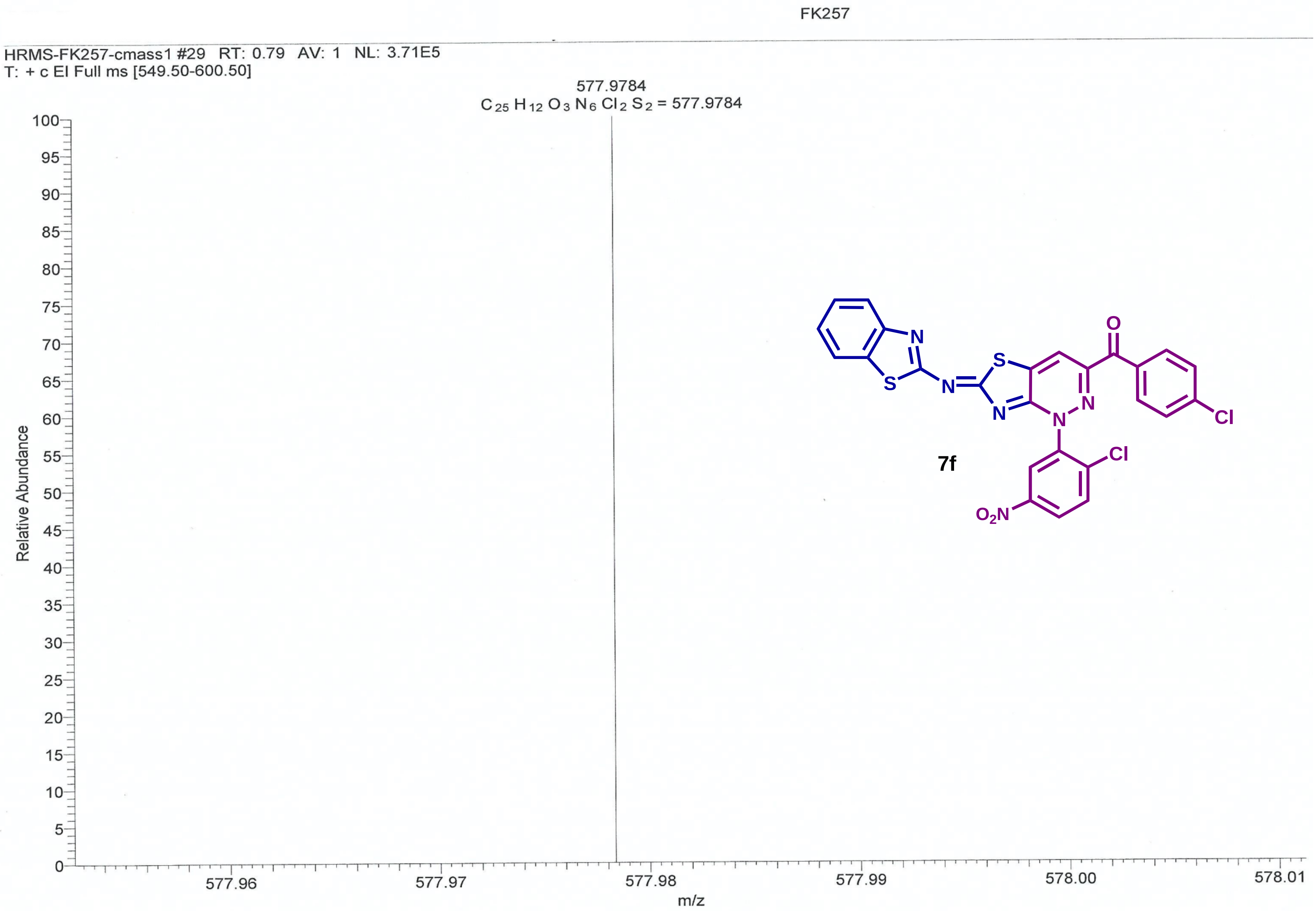

Figure S22. HRMS Spectra for compound **7f**.

1H spectra Dr.Hamada FK 257 in TFA-d

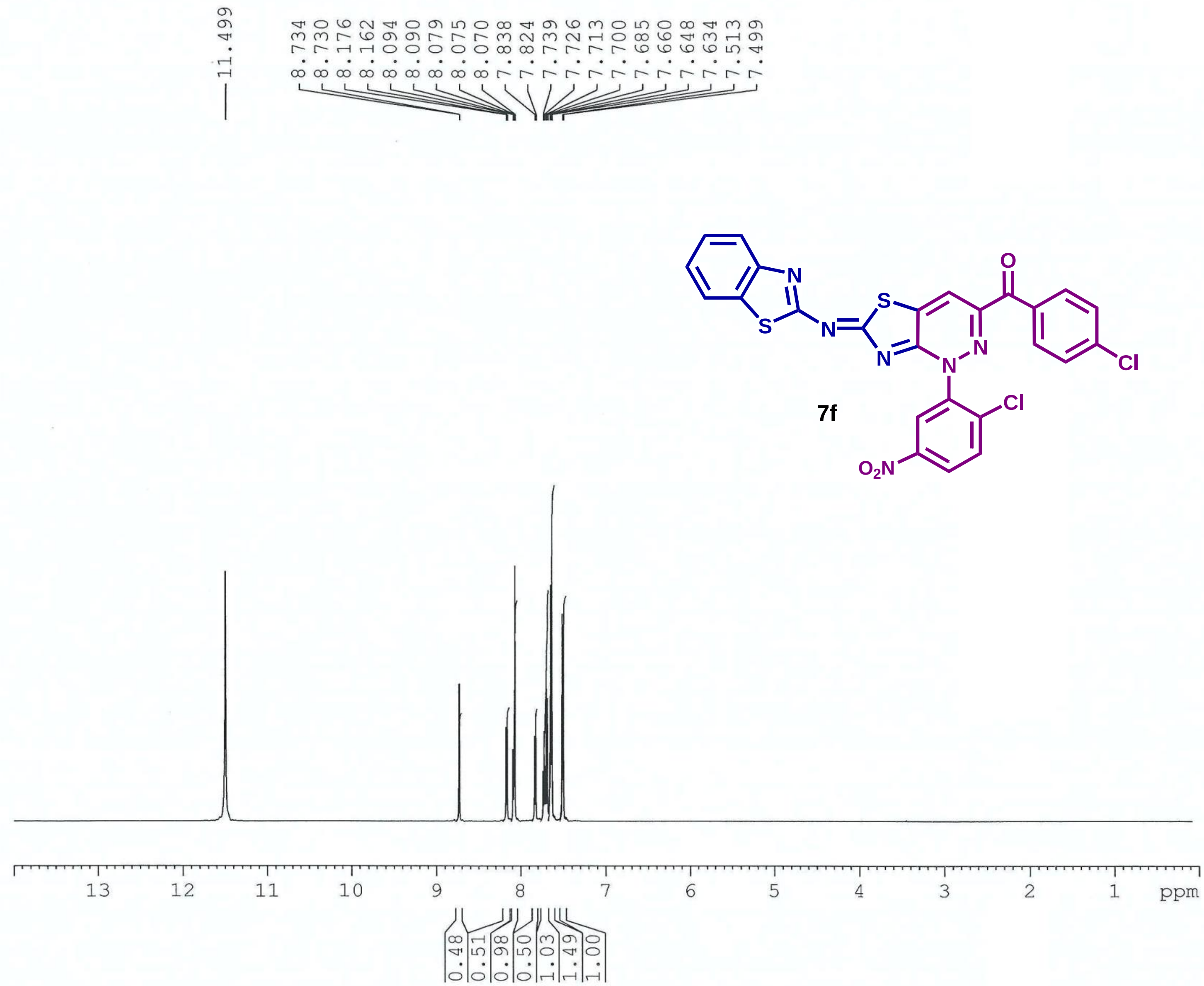

7f

Current Data Parameters
NAME FK257
EXPNO 1
PROCNO 1

F2 - Acquisition Parameters

Time 14.00
INSTRUM spect
PROBHD 5 mm PABBO BB-
PULPROG zg30
TD 65536
SOLVENT TFA
NS 16
DS 2
SWH 12335.526 Hz
FIDRES 0.188225 Hz
AQ 2.6563926 sec
RG 203
DW 40.533 usec
DE 20.00 usec
TE 298.0 K
D1 1.00000000 sec
TD0 1

======== CHANNEL f1 ========
SFO1 600.1337060 MHz
NUC1 1H
P1 10.60 usec
PLW1 27.82500076 W

F2 - Processing parameters
SI 32768
SF 600.1274888 MHz
WDW EM
SSB 0
LB 0.30 Hz
GB 0
PC 1.00

Figure S23. ^{1}H NMR Spectra (TFA-*d*, 600 MHz) for compound **7f**.

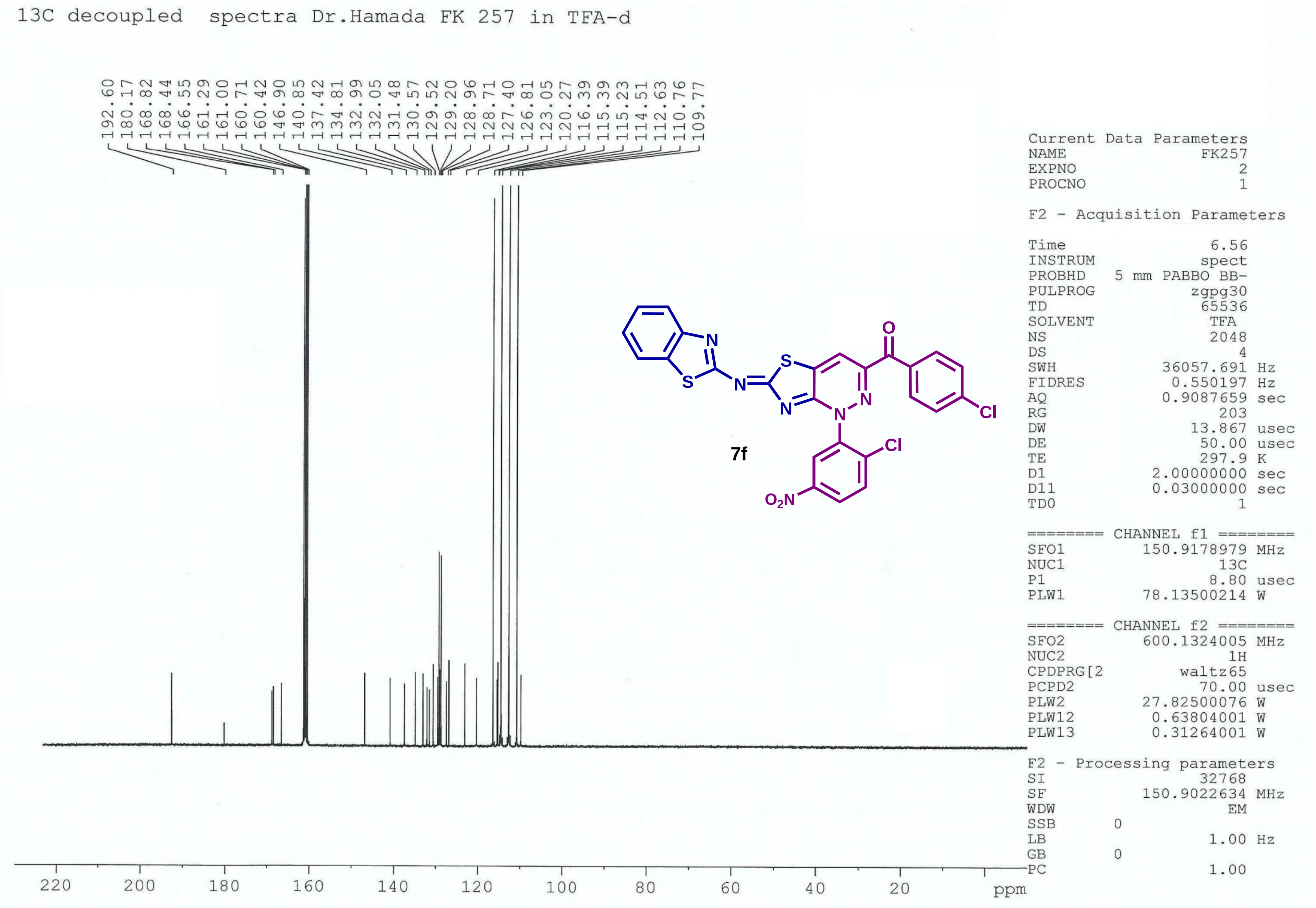

Figure S24. ^{13}C NMR Spectra (TFA-*d*, 150 MHz) for compound **7f**.

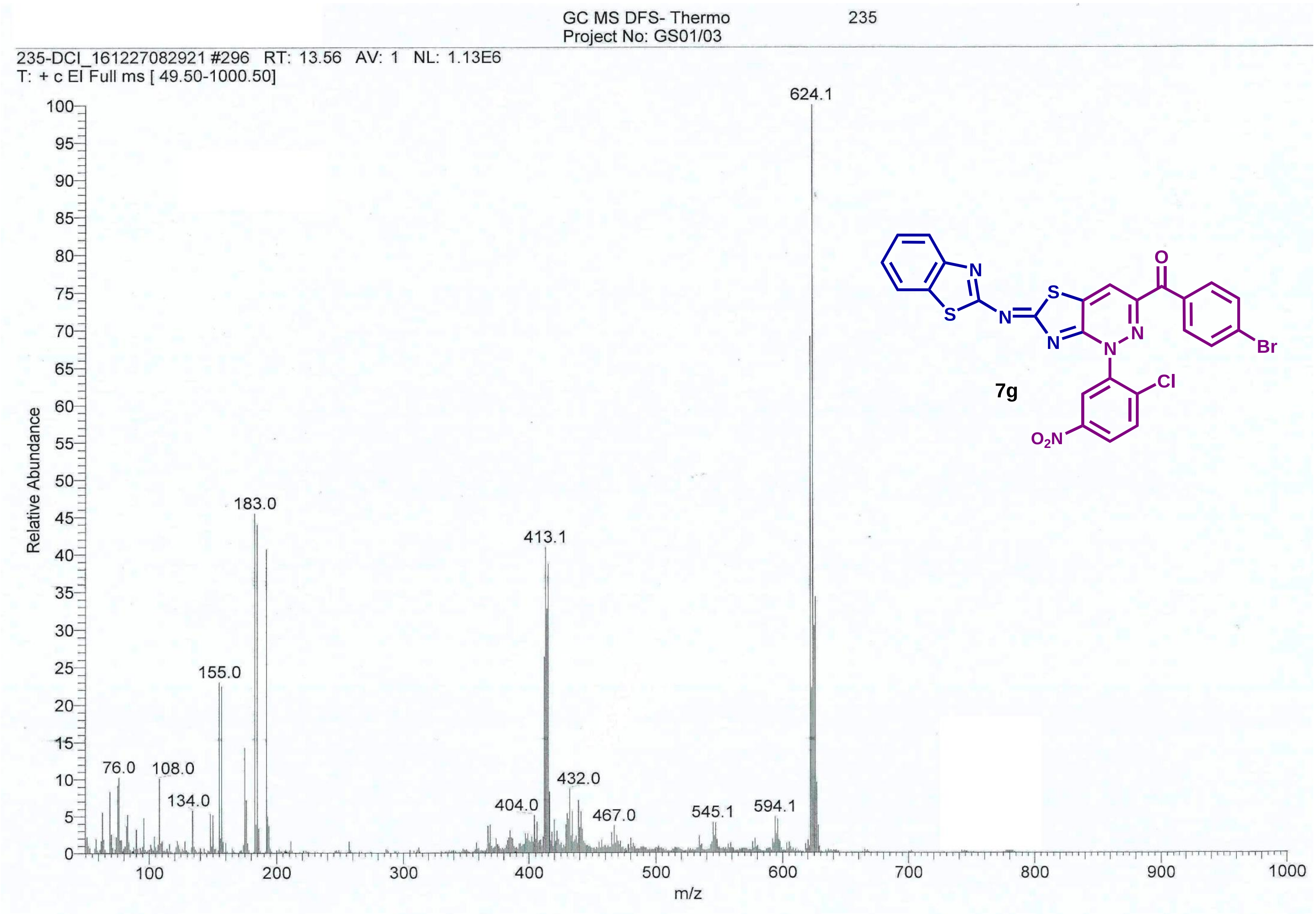

Figure S25. Mass Spectra for compound **7g**.

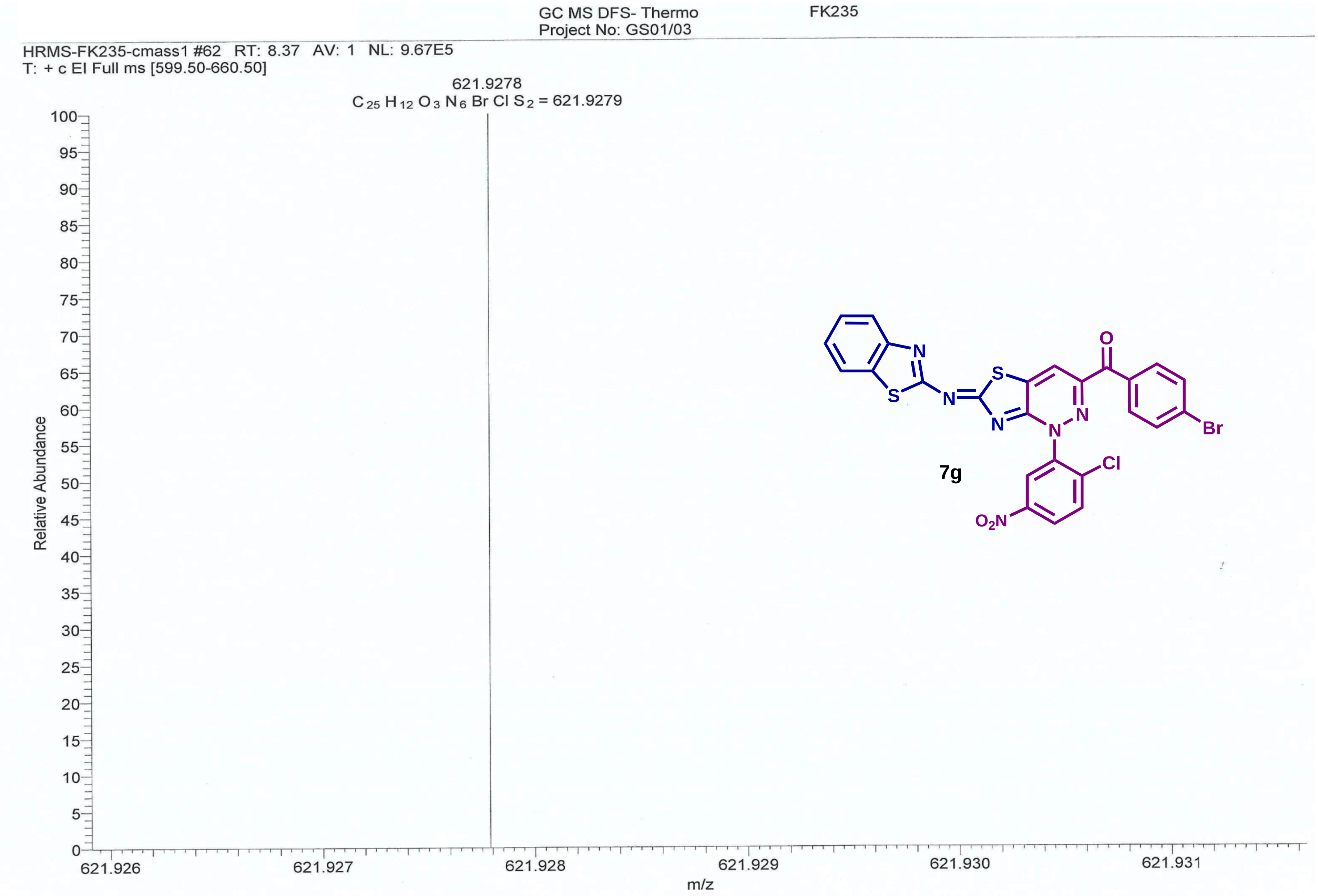

Figure S26. HRMS Spectra for compound **7g**.

1H spectrum Dr.Hamada FK 235 in THF

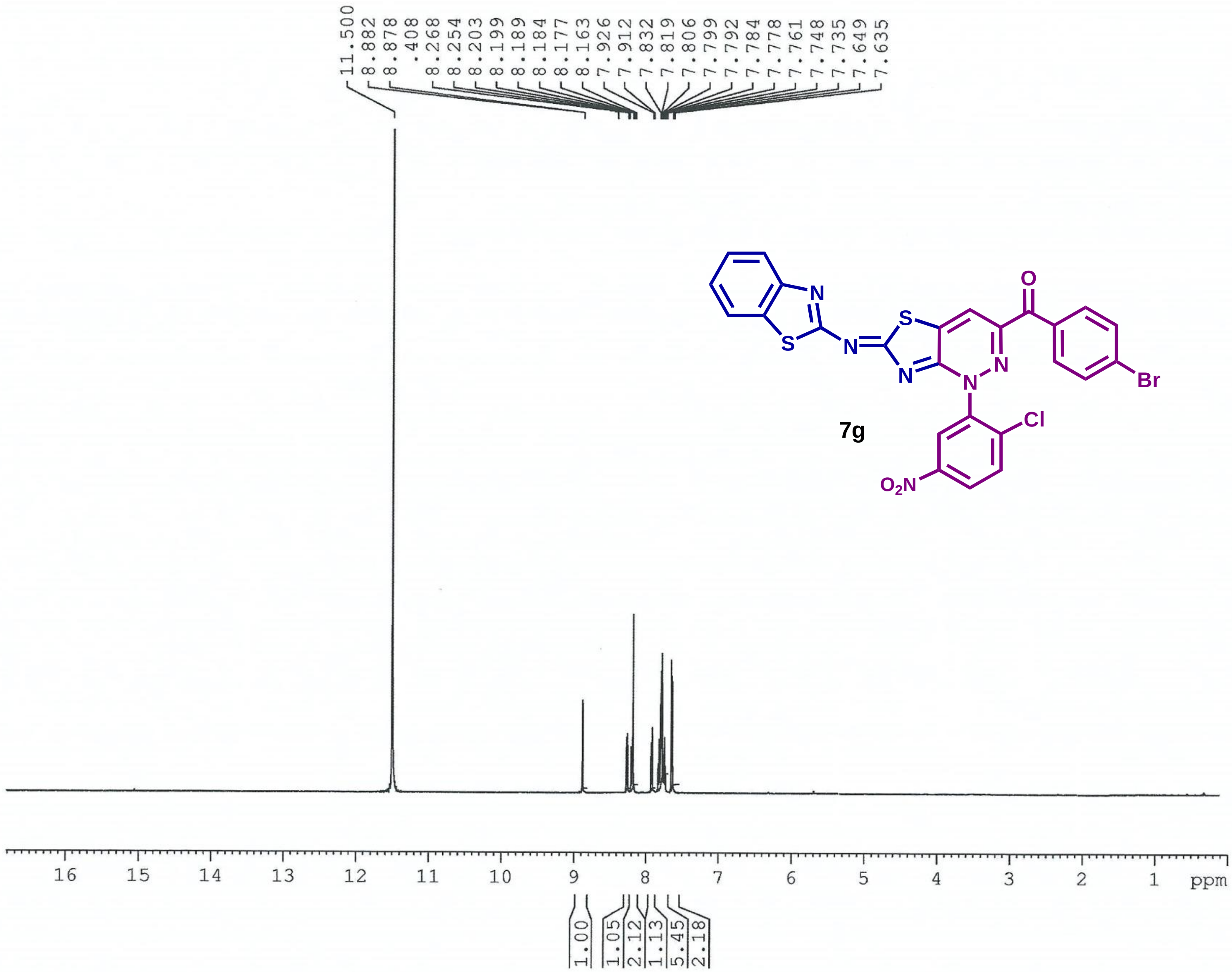

Current Data Parameters
NAME fk235-1H
EXPNO 1
PROCNO 1

F2 - Acquisition Parameters

Time 14.49
INSTRUM spect
PROBHD 5 mm PABBO BB-
PULPROG zg30
TD 65536
SOLVENT TFA
NS 8
DS 2
SWH 12335.526 Hz
FIDRES 0.188225 Hz
AQ 2.6563926 sec
RG 203
DW 40.533 usec
DE 20.00 usec
TE 298.0 K
D1 1.00000000 sec
TD0 1

======== CHANNEL f1 ========
SFO1 600.1337060 MHz
NUC1 1H
P1 10.60 usec
PLW1 27.82500076 W

F2 - Processing parameters
SI 32768
SF 600.1274733 MHz
WDW EM
SSB 0
LB 0.30 Hz
GB 0
PC 1.00

Figure S27. ^{1}H NMR Spectra (TFA-*d*, 600 MHz) for compound **7g**.

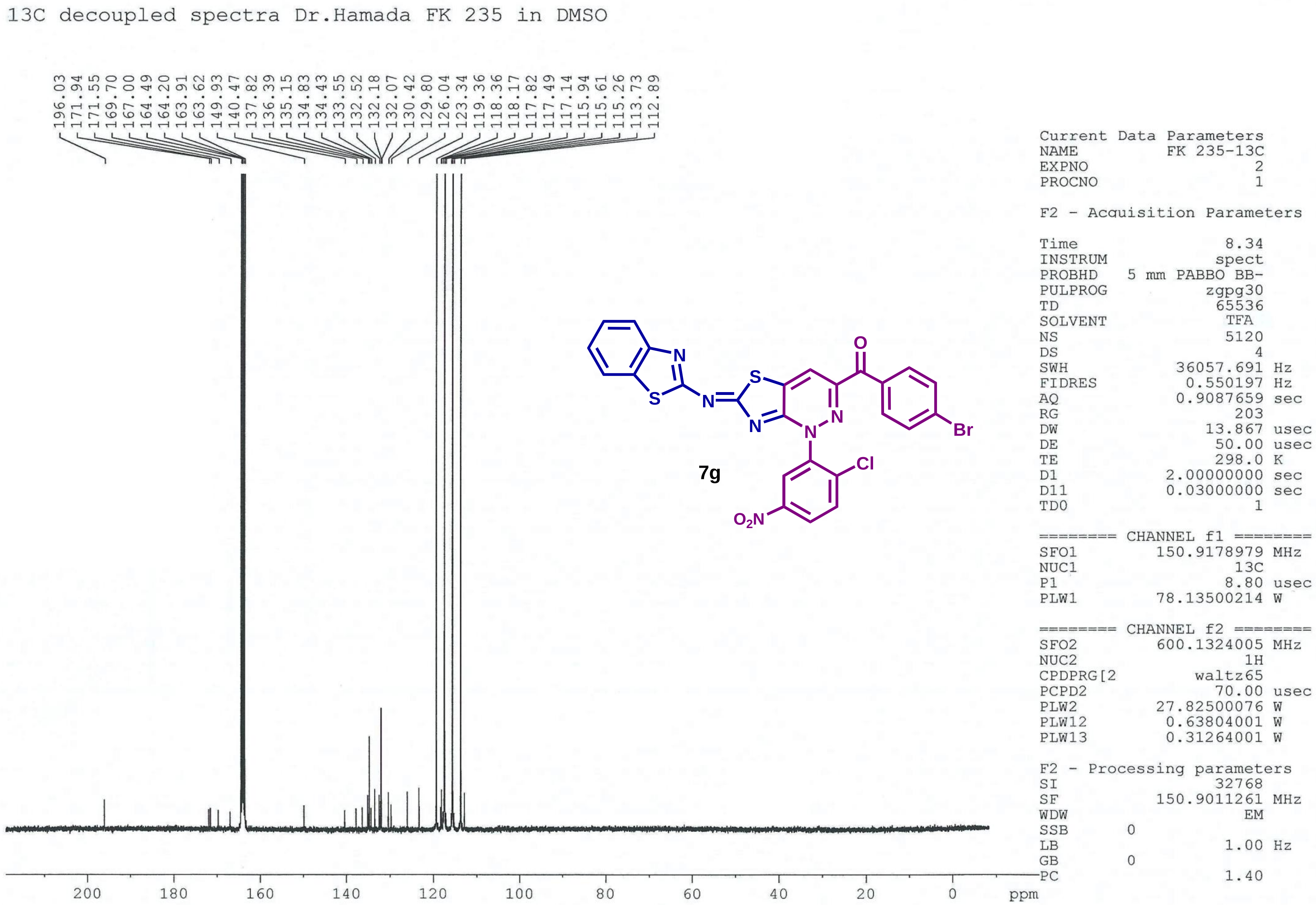

Figure S28. ^{13}C NMR Spectra (TFA-*d*, 150 MHz) for compound **7g**.

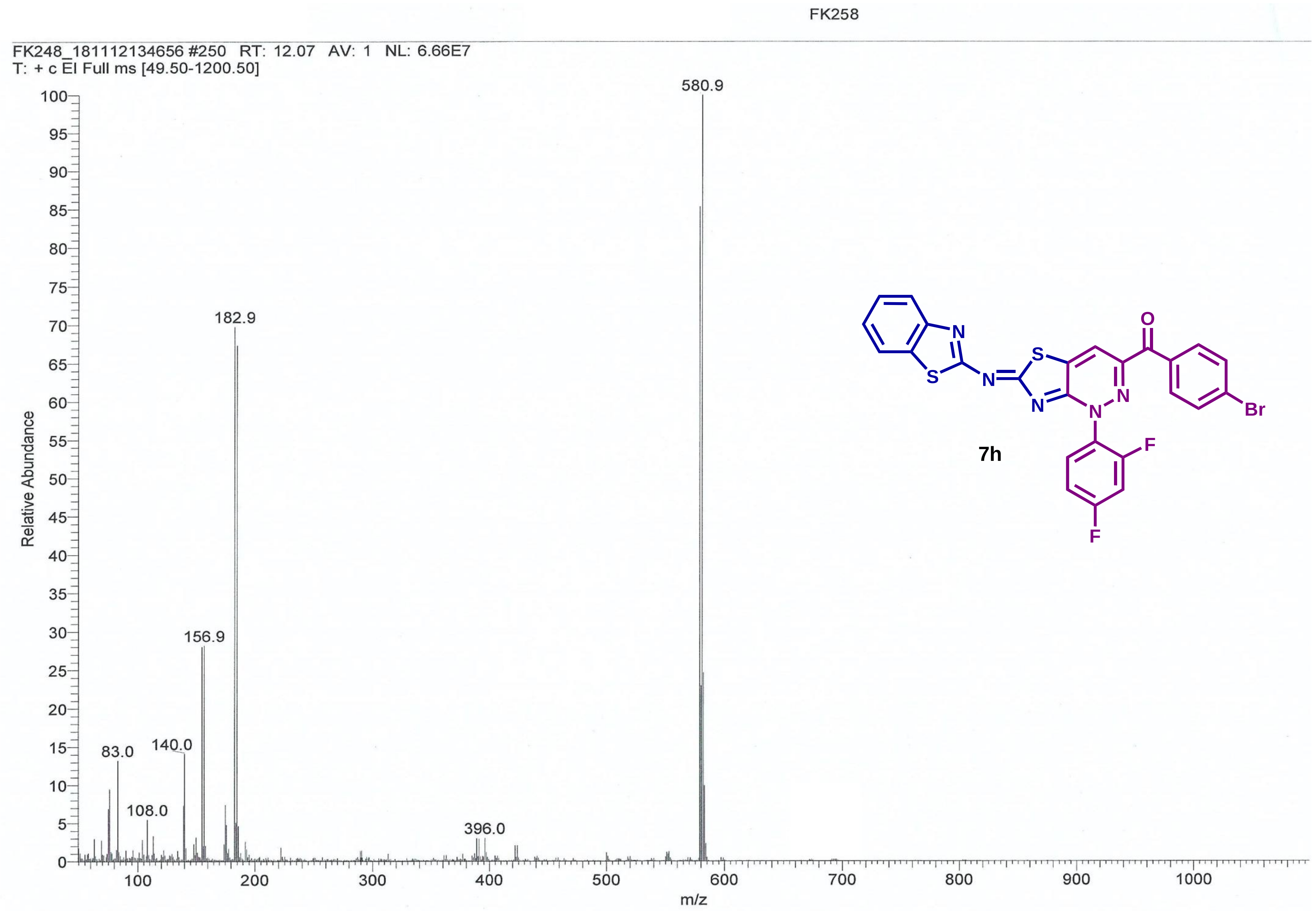

Figure S29. Mass Spectra for compound **7h**.

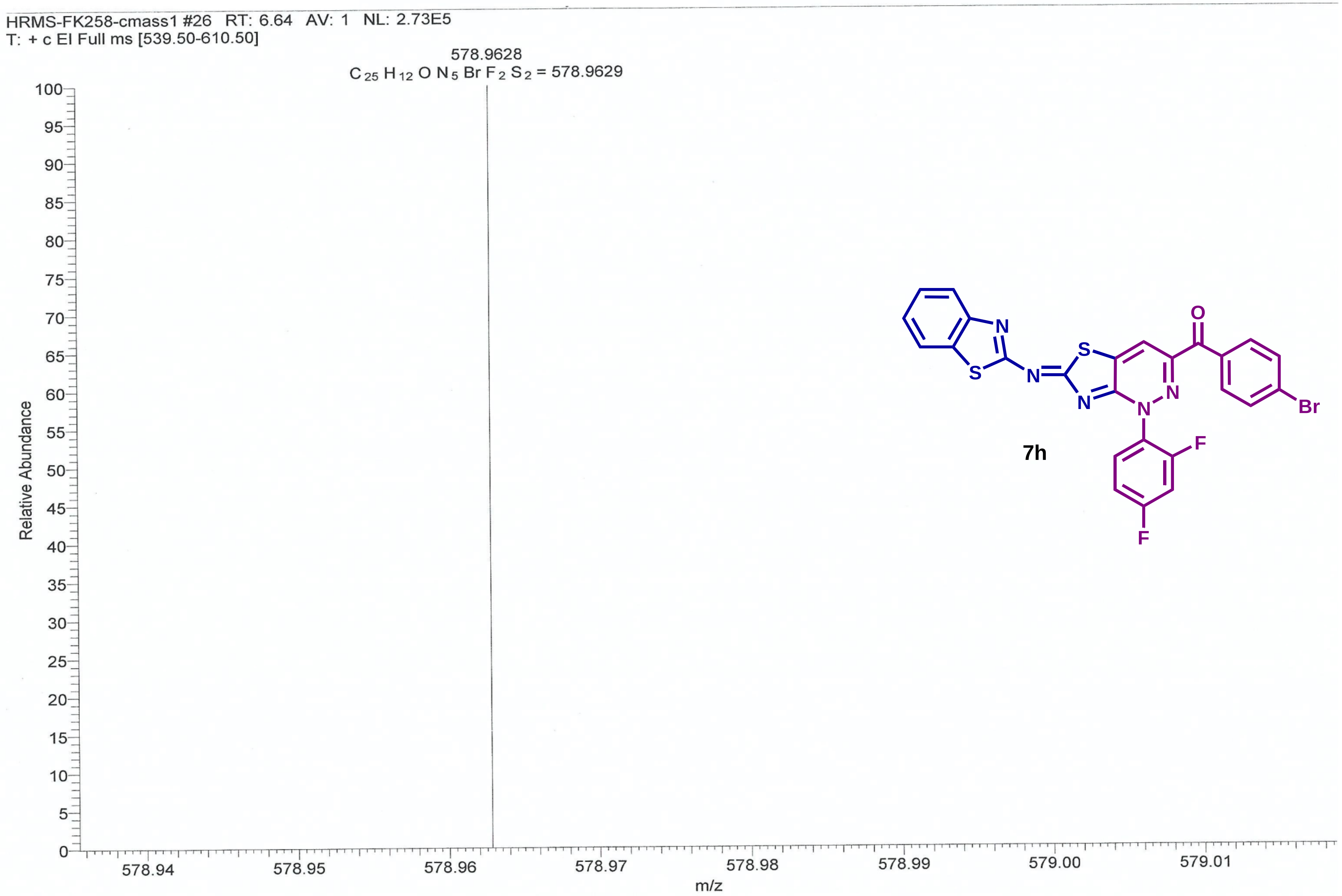

Figure S30. HRMS Spectra for compound **7h**.

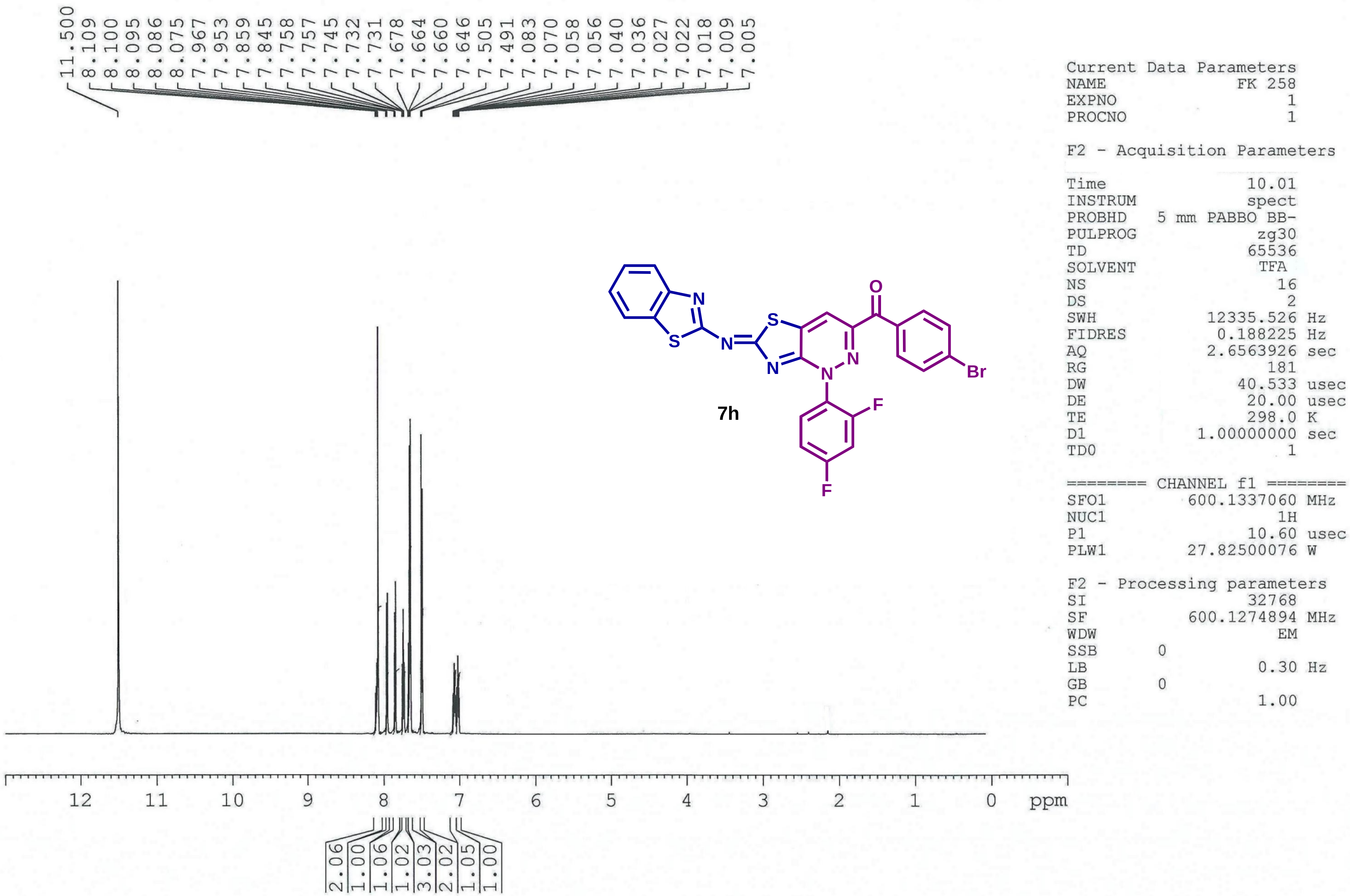

Figure S31. ^1H NMR Spectra (TFA-*d*, 600 MHz) for compound **7h**.

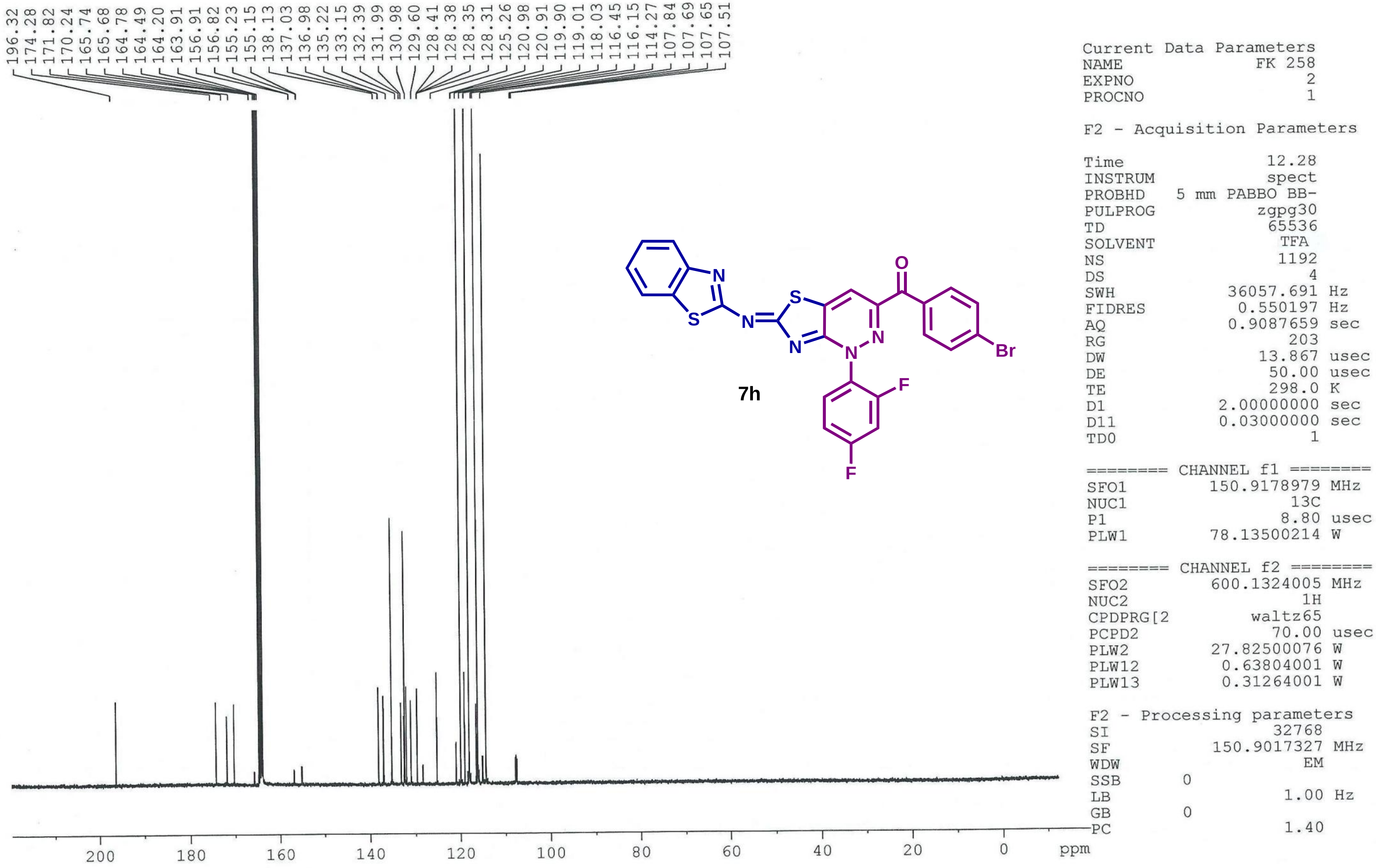

Figure S32. ^{13}C NMR Spectra (TFA-*d*, 150 MHz) for compound **7h**.

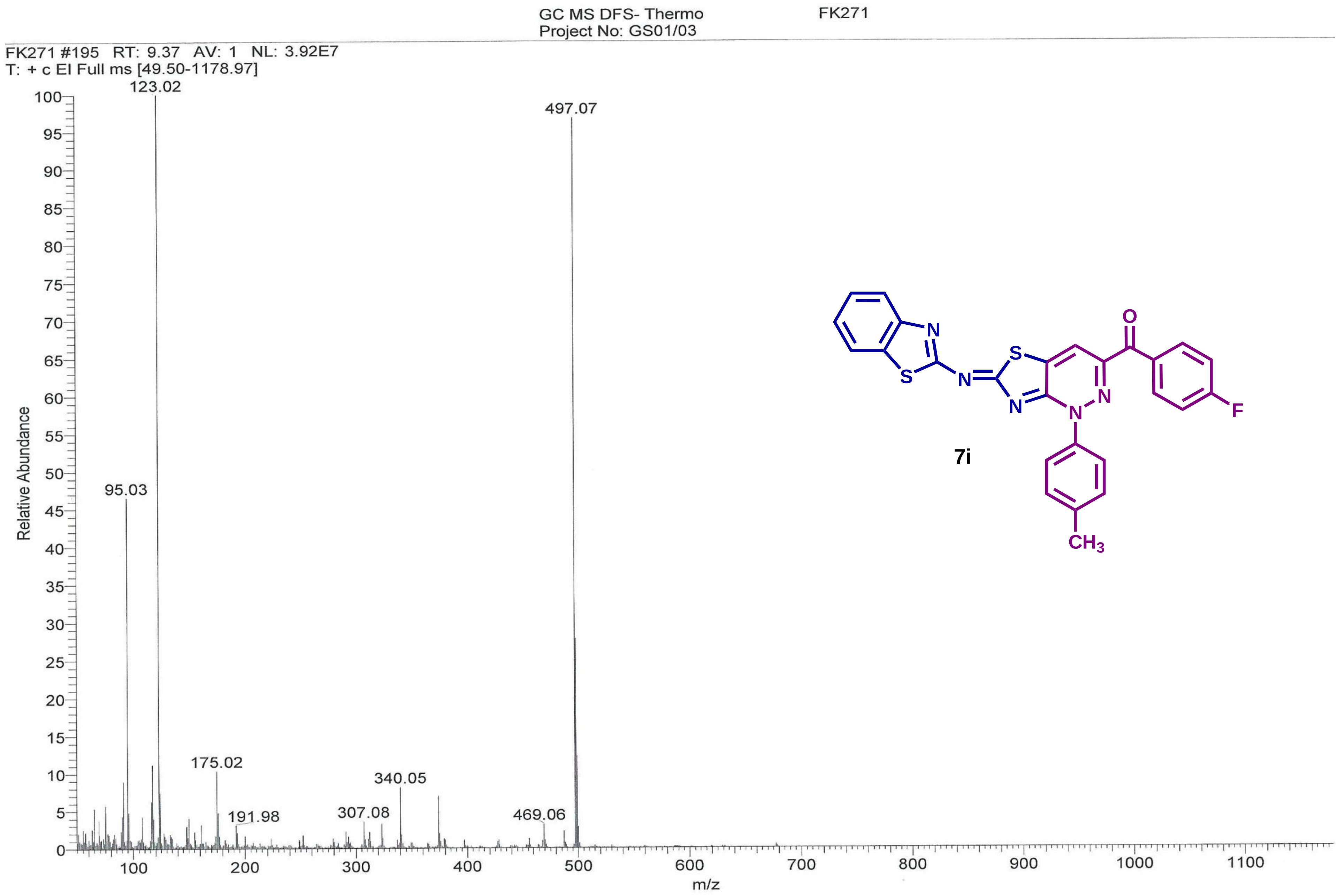

Figure S33. Mass Spectra for compound **7i**.

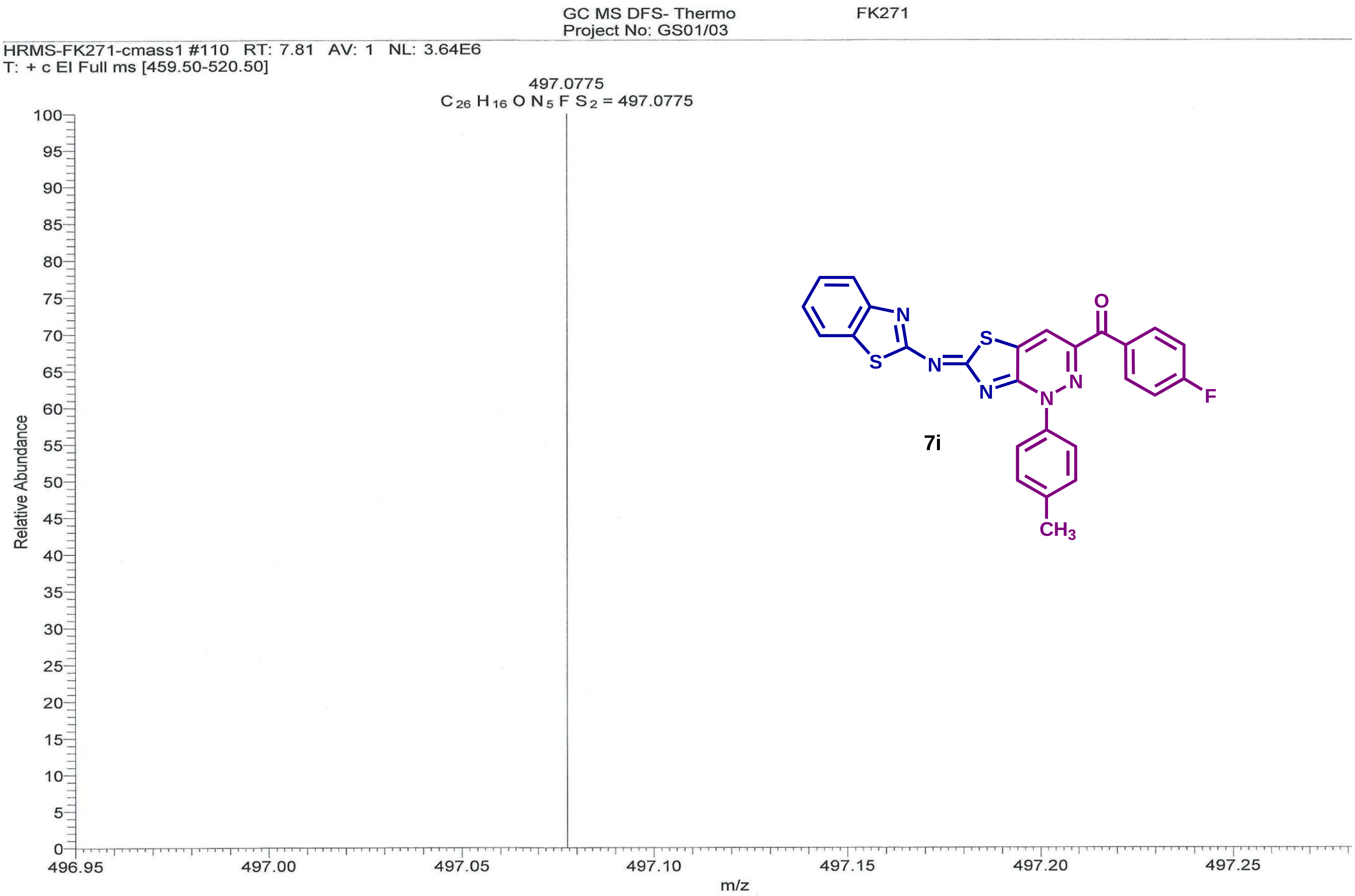

Figure S34. HRMS Spectra for compound **7i**.

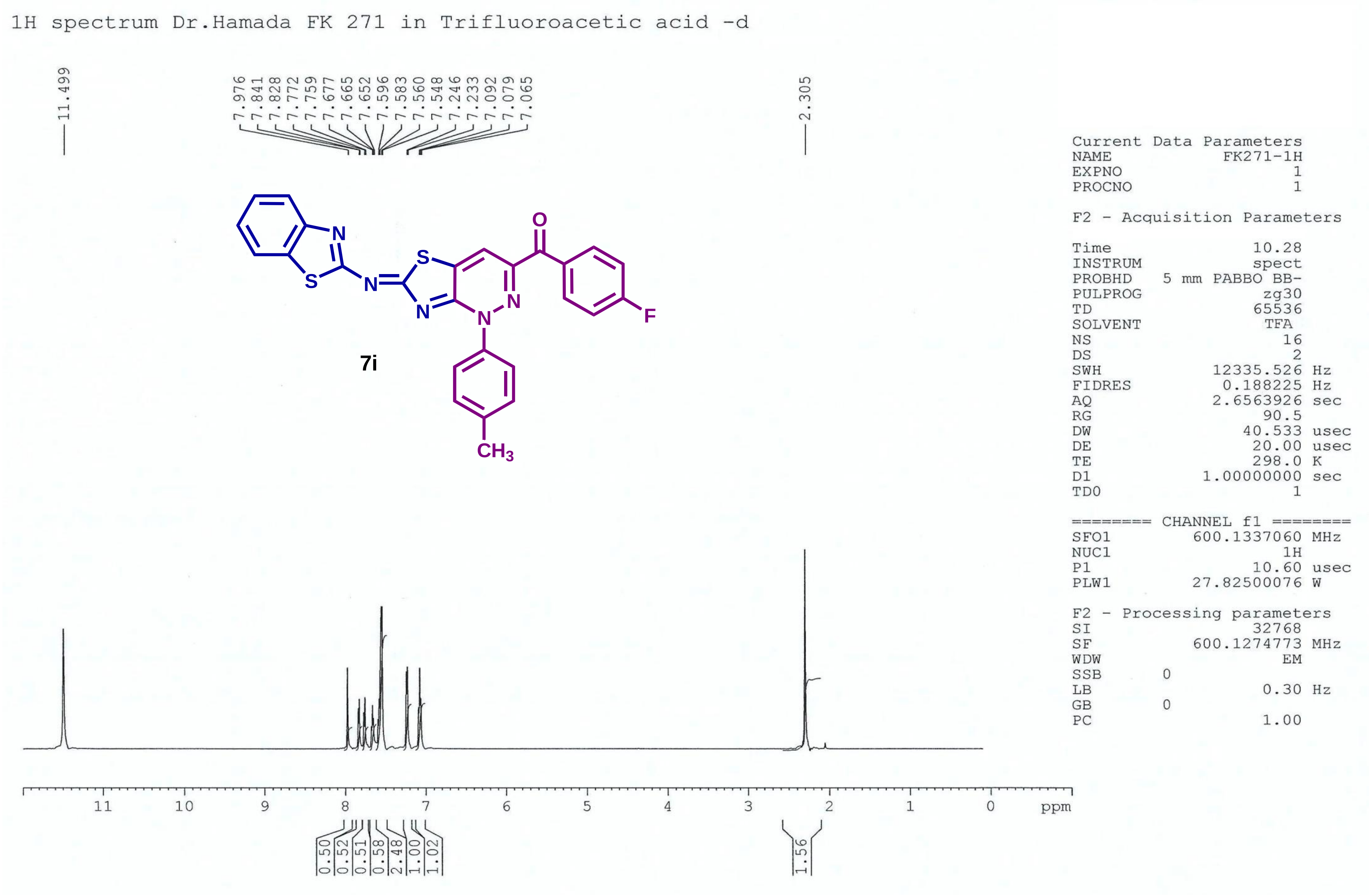

Figure S35. ^{1}H NMR Spectra (TFA-*d*, 600 MHz) for compound **7i**.

13C decoupled spectrum Dr.Hamada FK271 in TFA-d

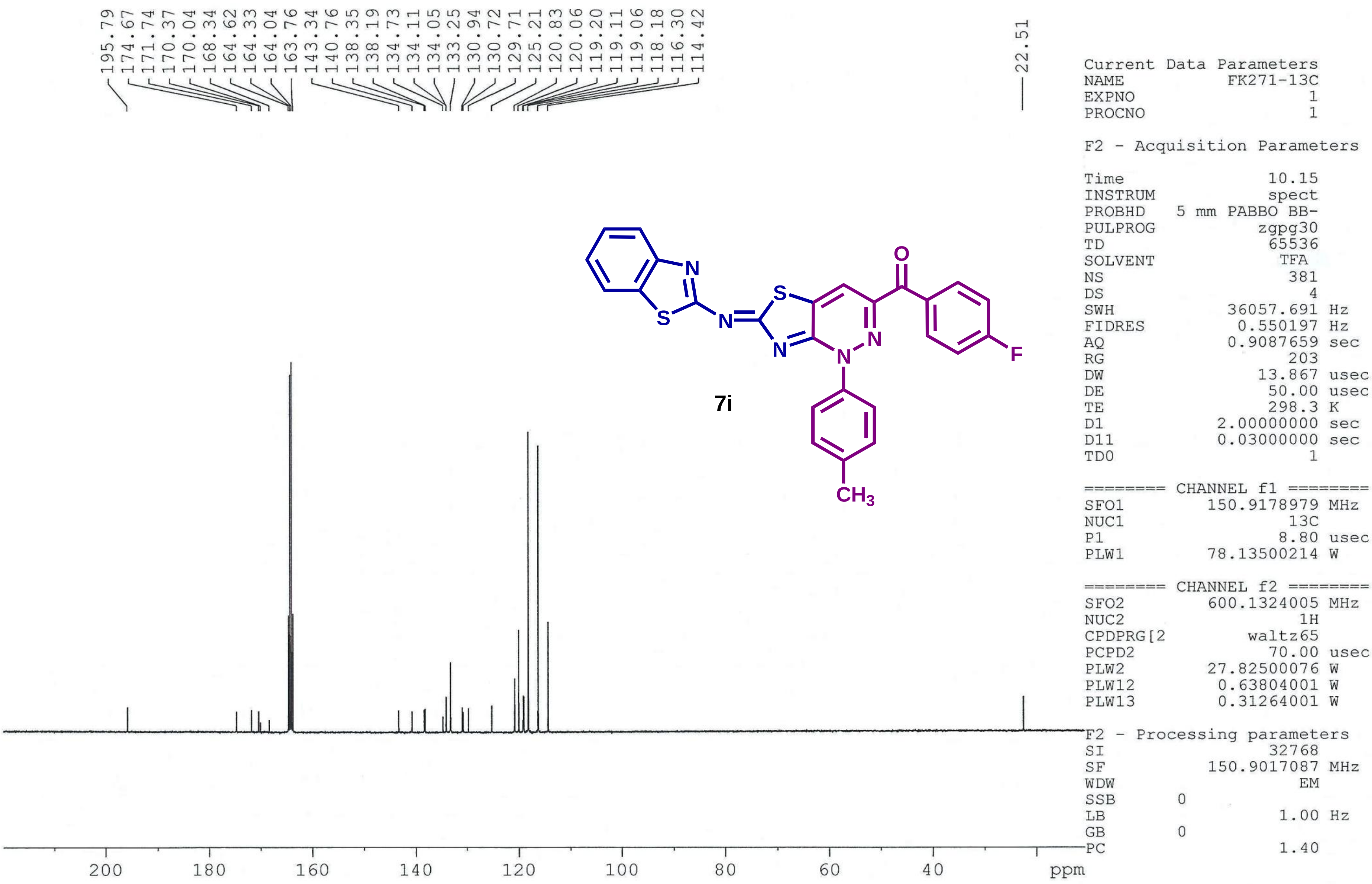


```
Current Data Parameters
NAME                FK271-13C
EXPNO                       1
PROCNO                      1

F2 - Acquisition Parameters
Time                    10.15
INSTRUM                 spect
PROBHD     5 mm PABBO BB-
PULPROG               zgpg30
TD                      65536
SOLVENT                   TFA
NS                        381
DS                          4
SWH              36057.691 Hz
FIDRES            0.550197 Hz
AQ               0.9087659 sec
RG                        203
DW                 13.867 usec
DE                  50.00 usec
TE                    298.3 K
D1              2.00000000 sec
D11             0.03000000 sec
TD0                         1

======== CHANNEL f1 ========
SFO1         150.9178979 MHz
NUC1                      13C
P1                   8.80 usec
PLW1             78.13500214 W

======== CHANNEL f2 ========
SFO2         600.1324005 MHz
NUC2                       1H
CPDPRG[2              waltz65
PCPD2               70.00 usec
PLW2             27.82500076 W
PLW12             0.63804001 W
PLW13             0.31264001 W

F2 - Processing parameters
SI                      32768
SF           150.9017087 MHz
WDW                        EM
SSB        0
LB                    1.00 Hz
GB         0
PC                       1.40
```

Figure S36. ^{13}C NMR Spectra (TFA-*d*, 150 MHz) for compound **7i**.

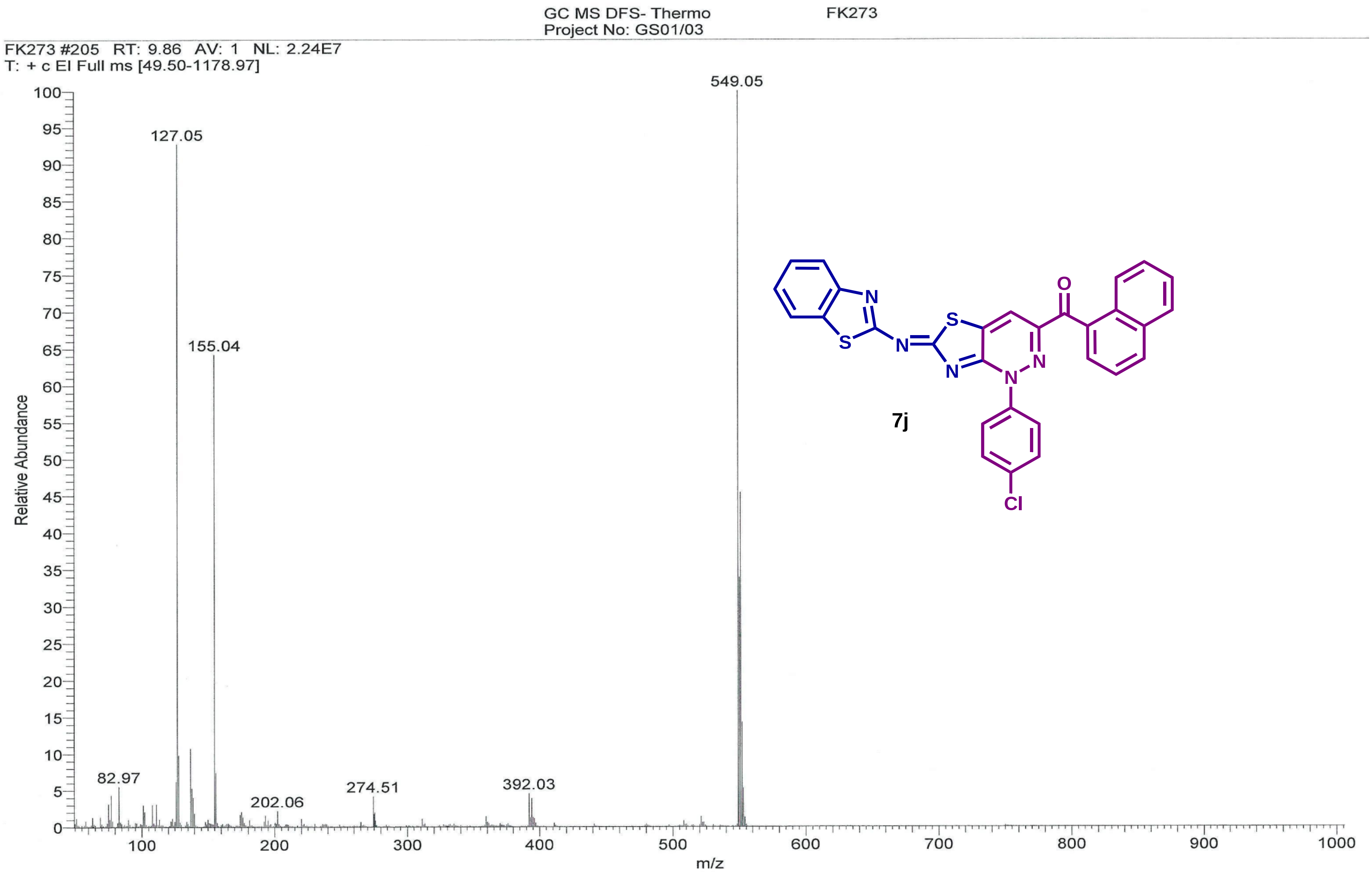

Figure S37. Mass Spectra for compound **7j**.

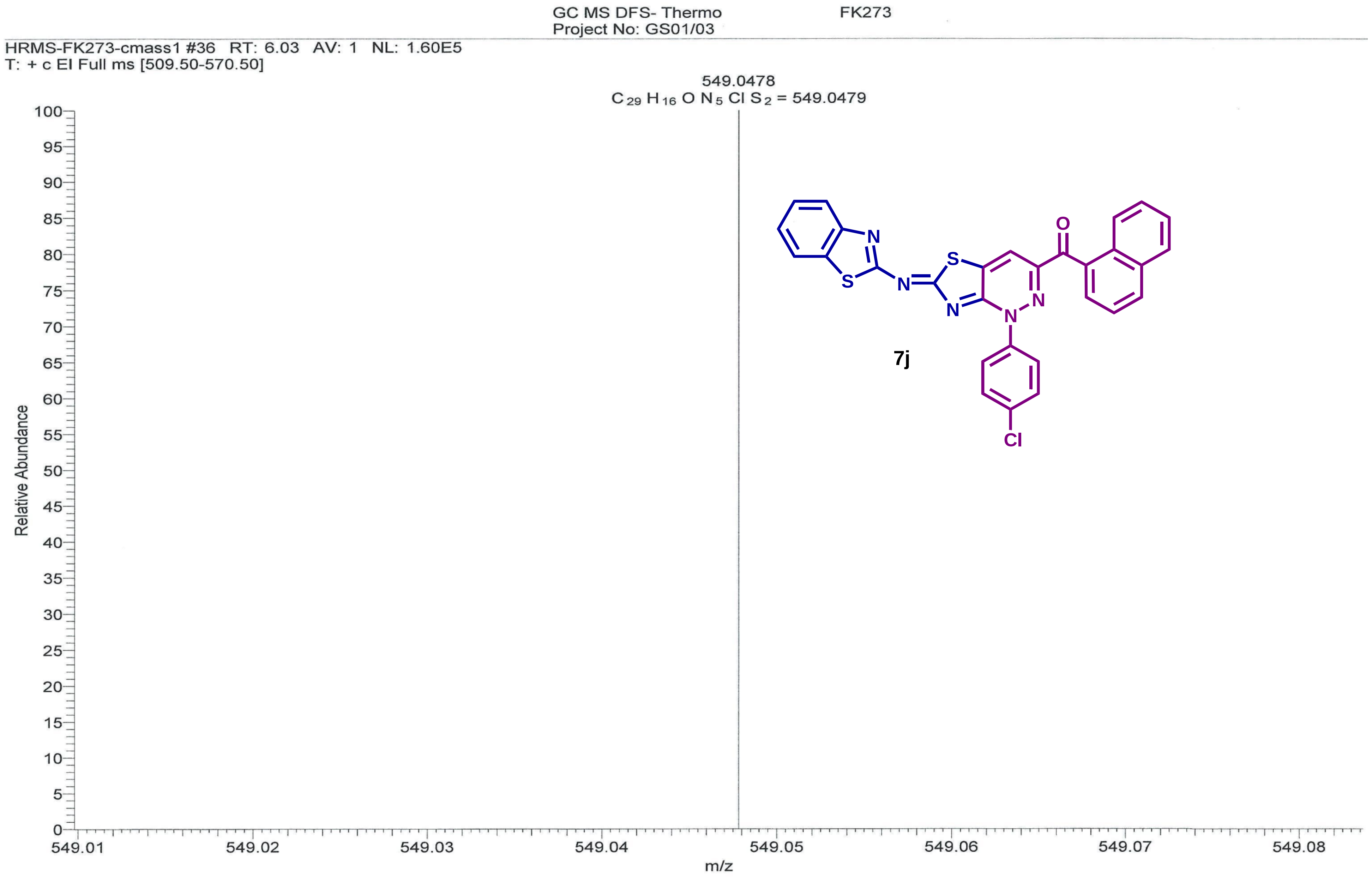

Figure S38. HRMS Spectra for compound **7j**.

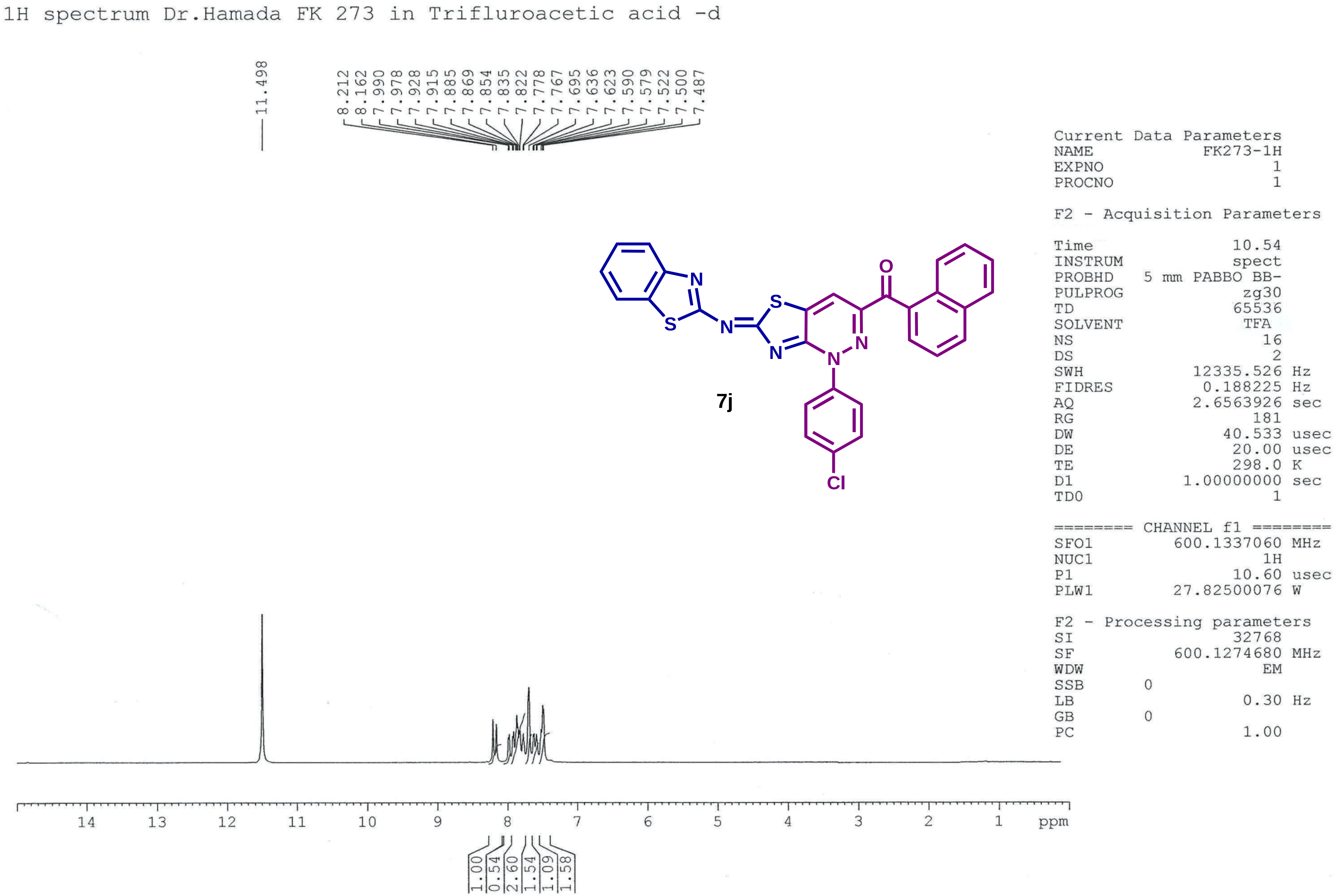

Figure S39. ^{1}H NMR Spectra (TFA-*d*, 600 MHz) for compound **7j**.

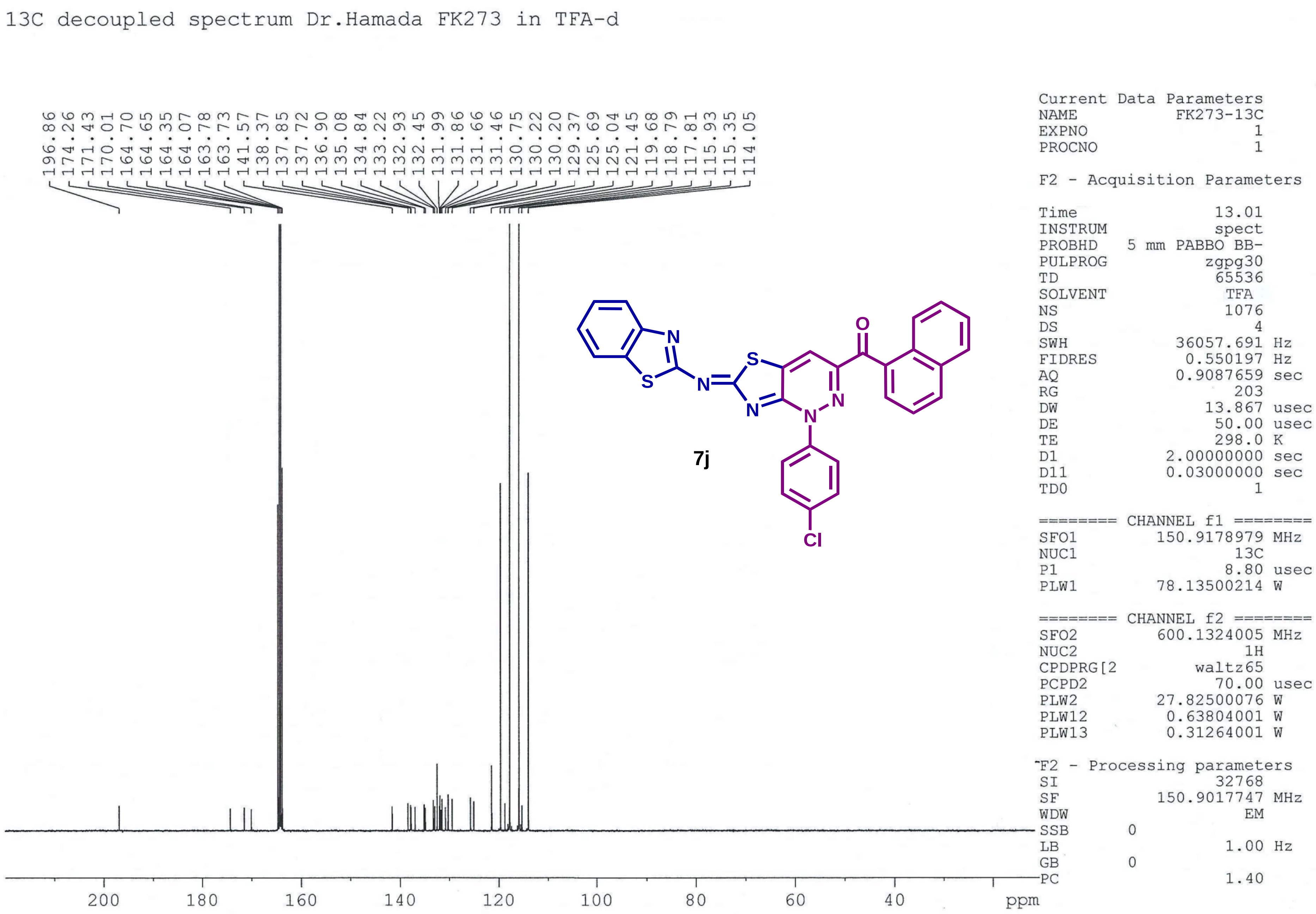

Figure S40. ^{13}C NMR Spectra (TFA-*d*, 150 MHz) for compound **7j**.

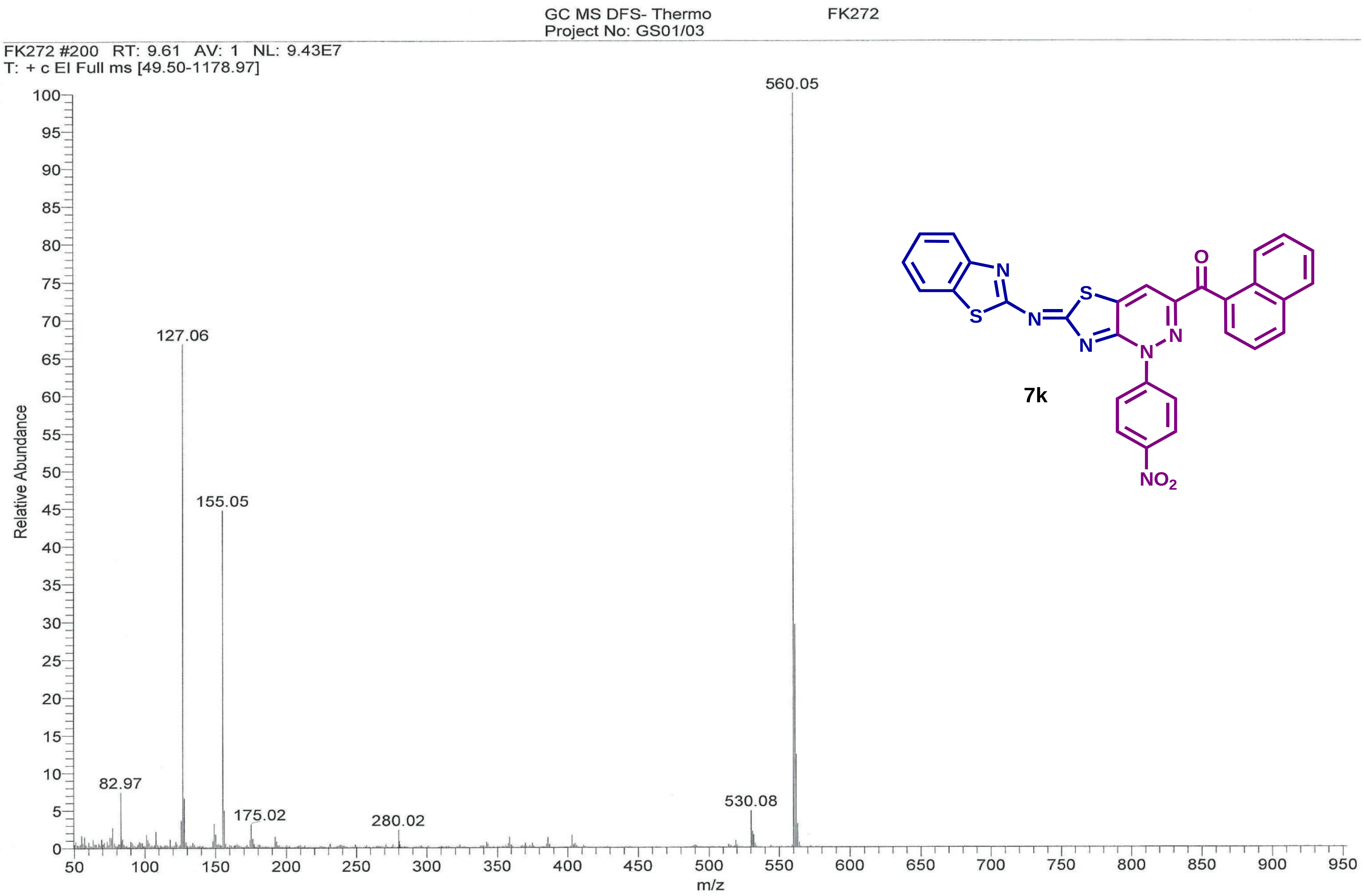

7k

Figure S41. Mass Spectra for compound **7k**.

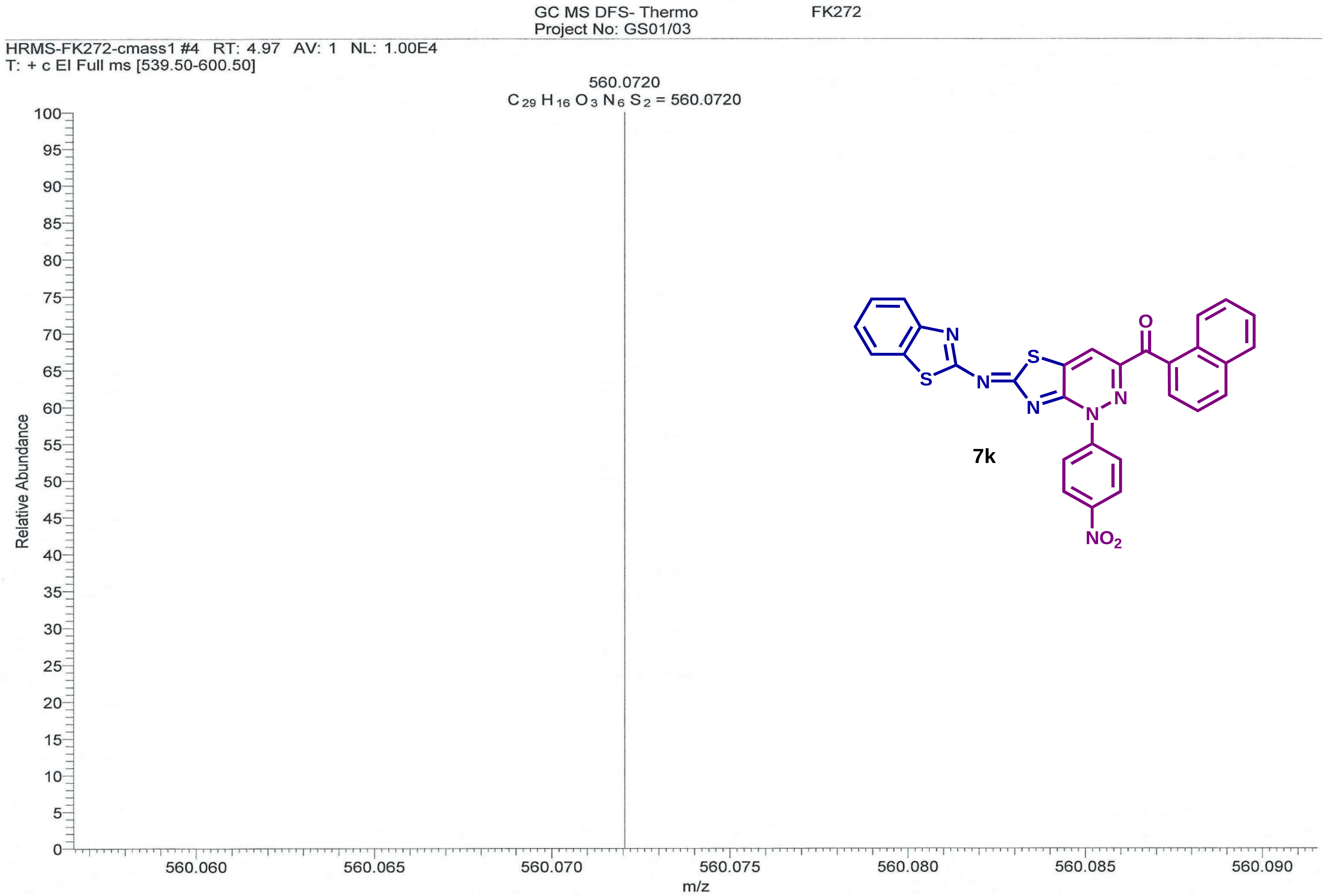

Figure S42. HRMS Spectra for compound **7k**.

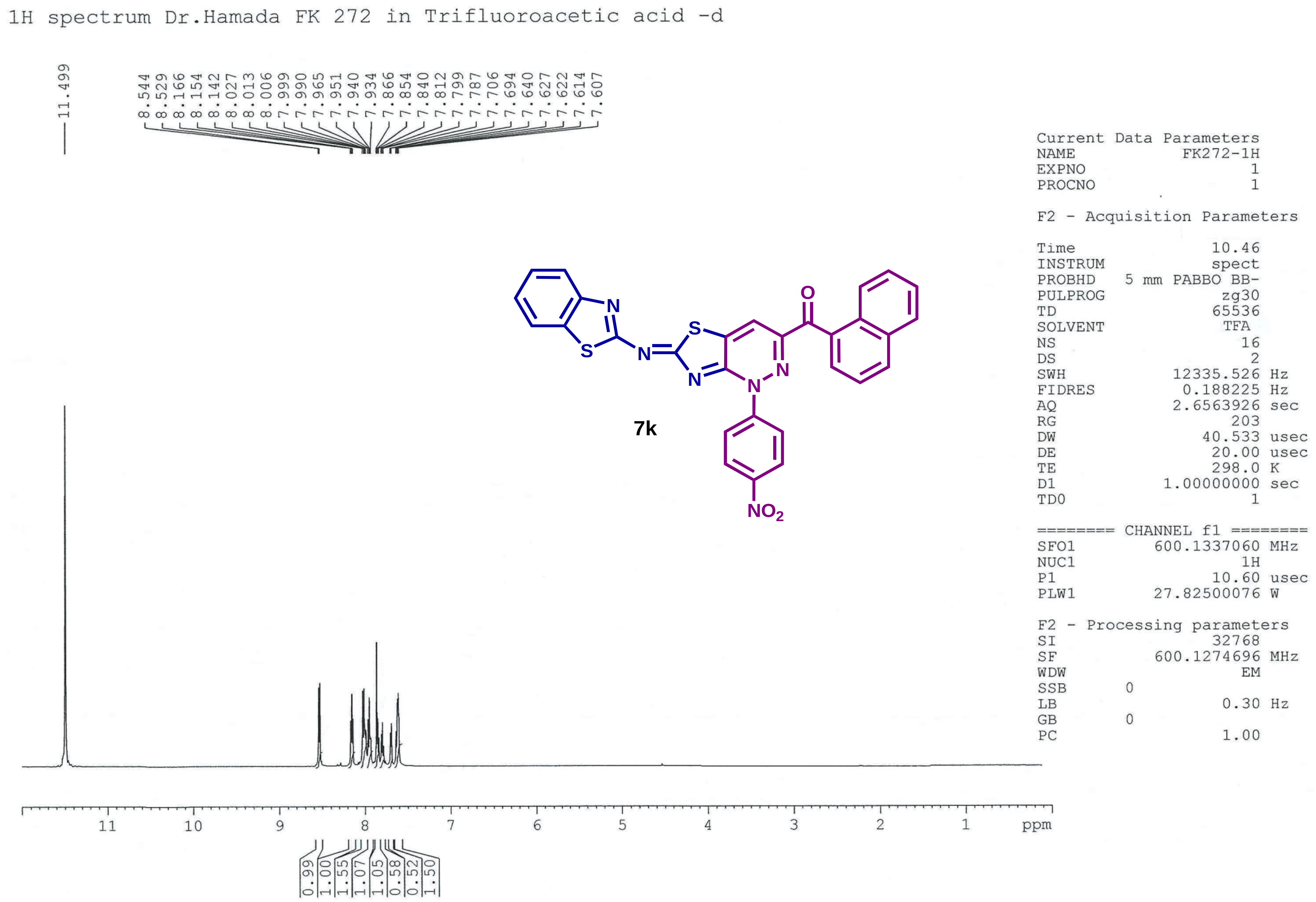

Figure S43. ^{1}H NMR Spectra (TFA-*d*, 600 MHz) for compound **7k**.

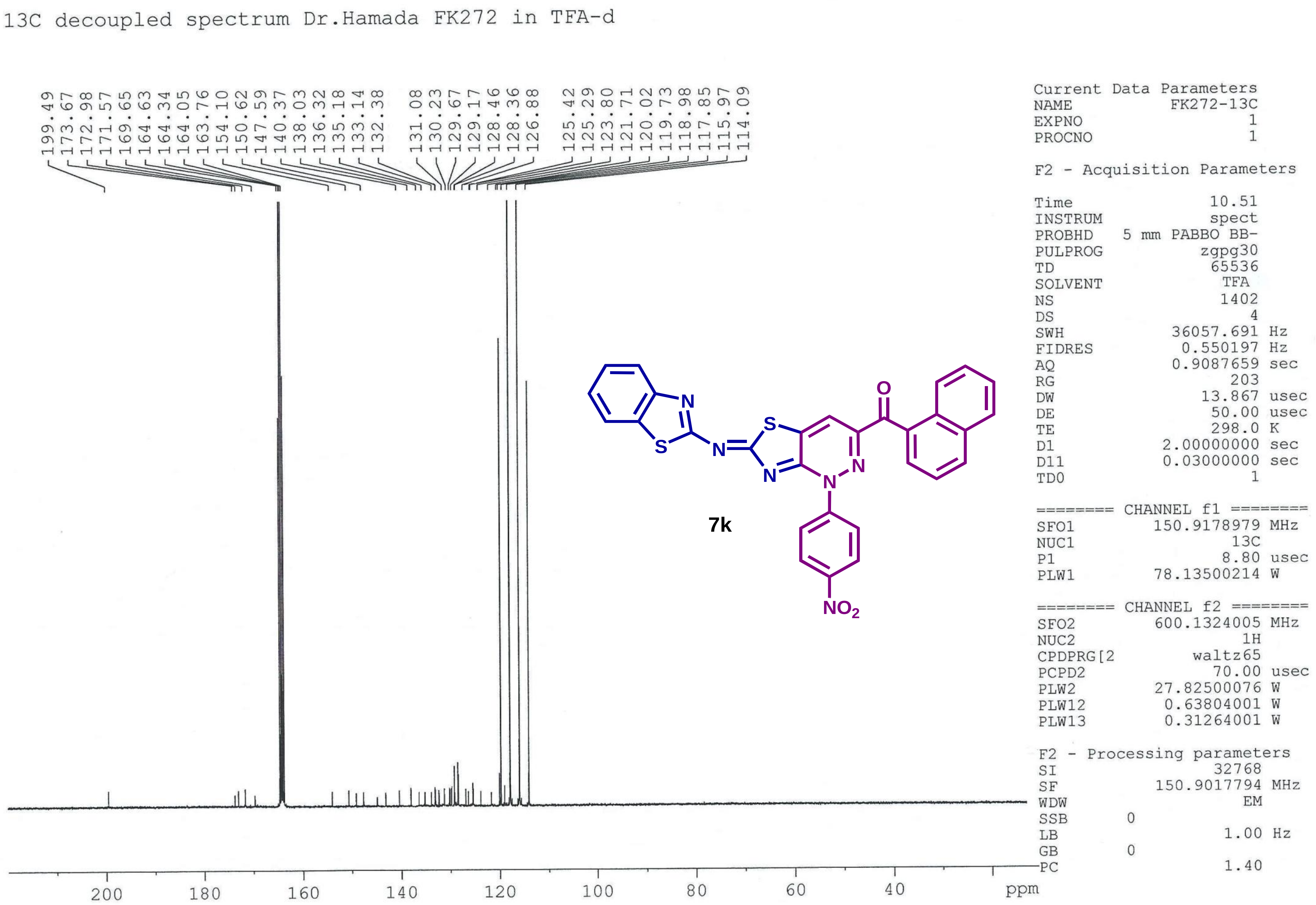

Figure S44. ^{13}C NMR Spectra (TFA-*d*, 150 MHz) for compound **7k**.

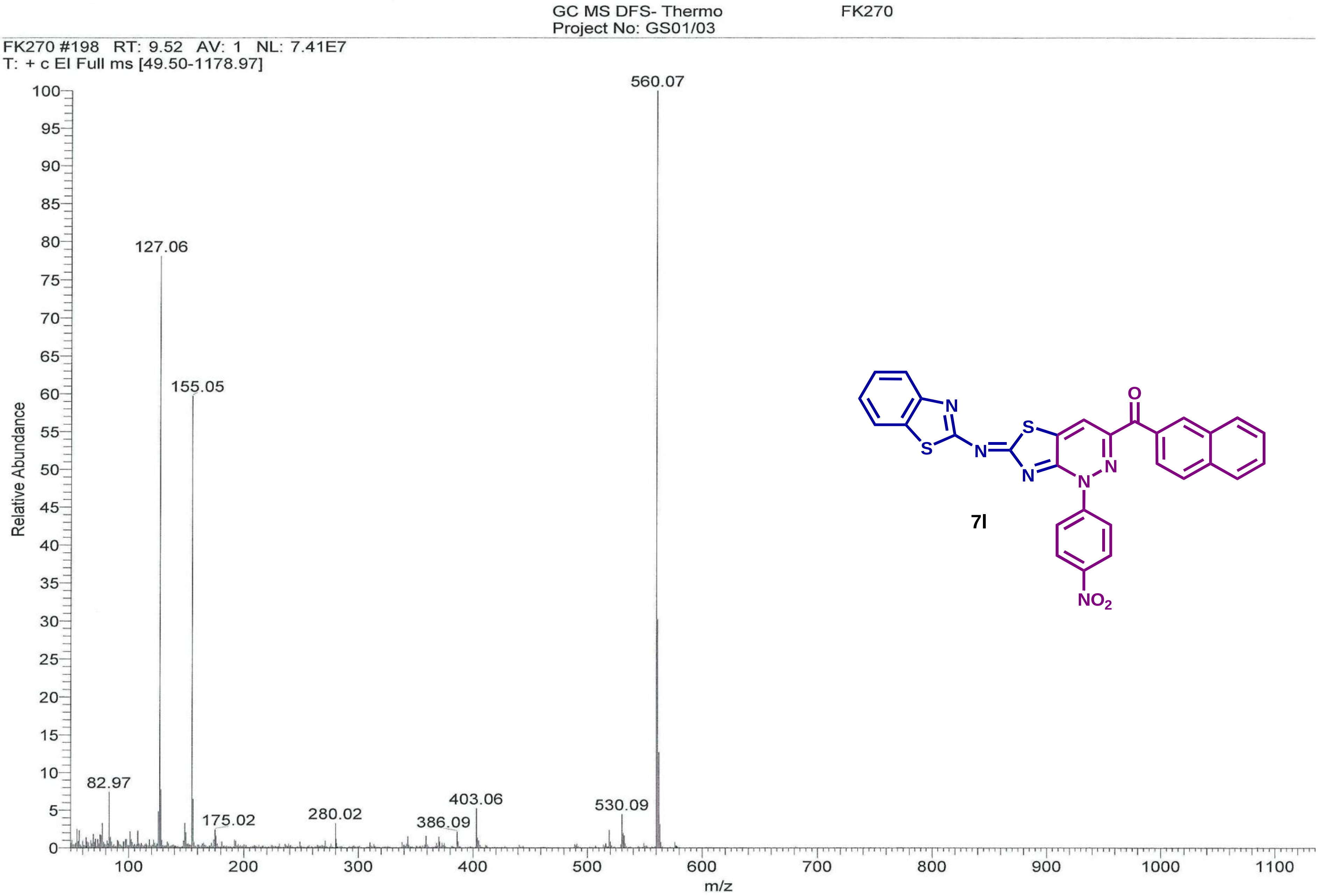

7l

Figure S45. Mass Spectra for compound **7l.**

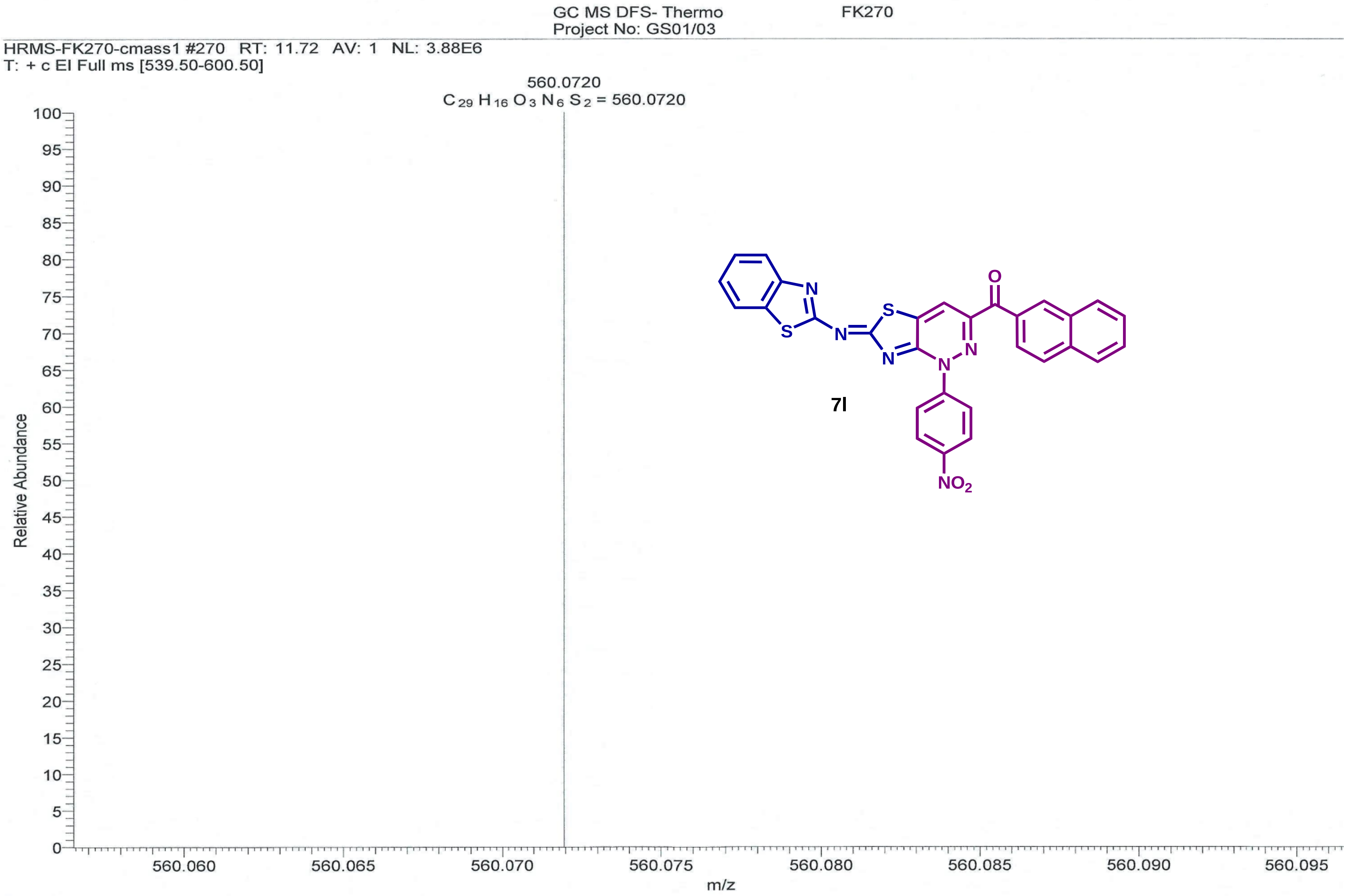

Figure S46. HRMS Spectra for compound **7l**.

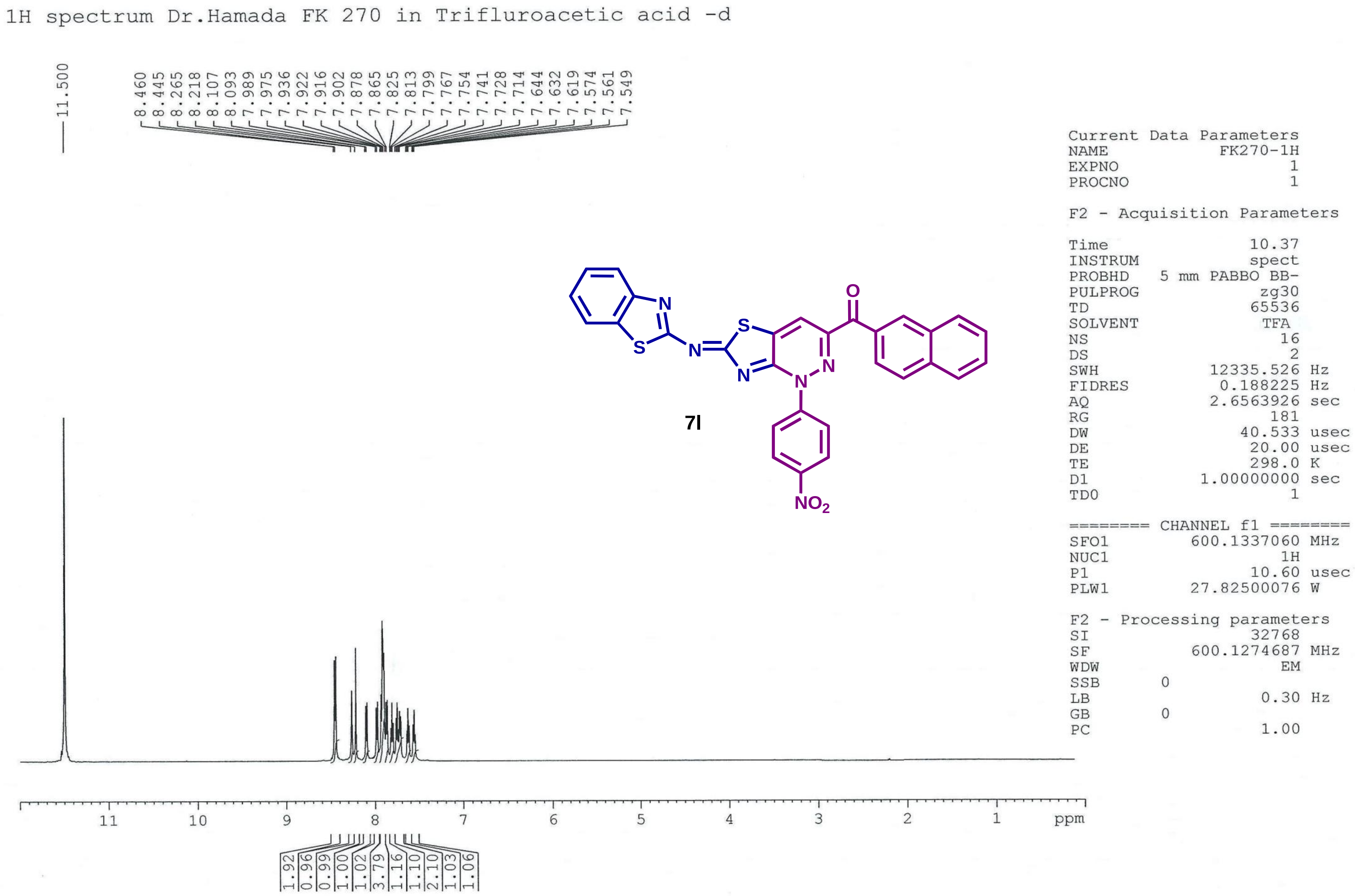

Figure S47. ^{1}H NMR Spectra (TFA-*d*, 600 MHz) for compound **7l**.

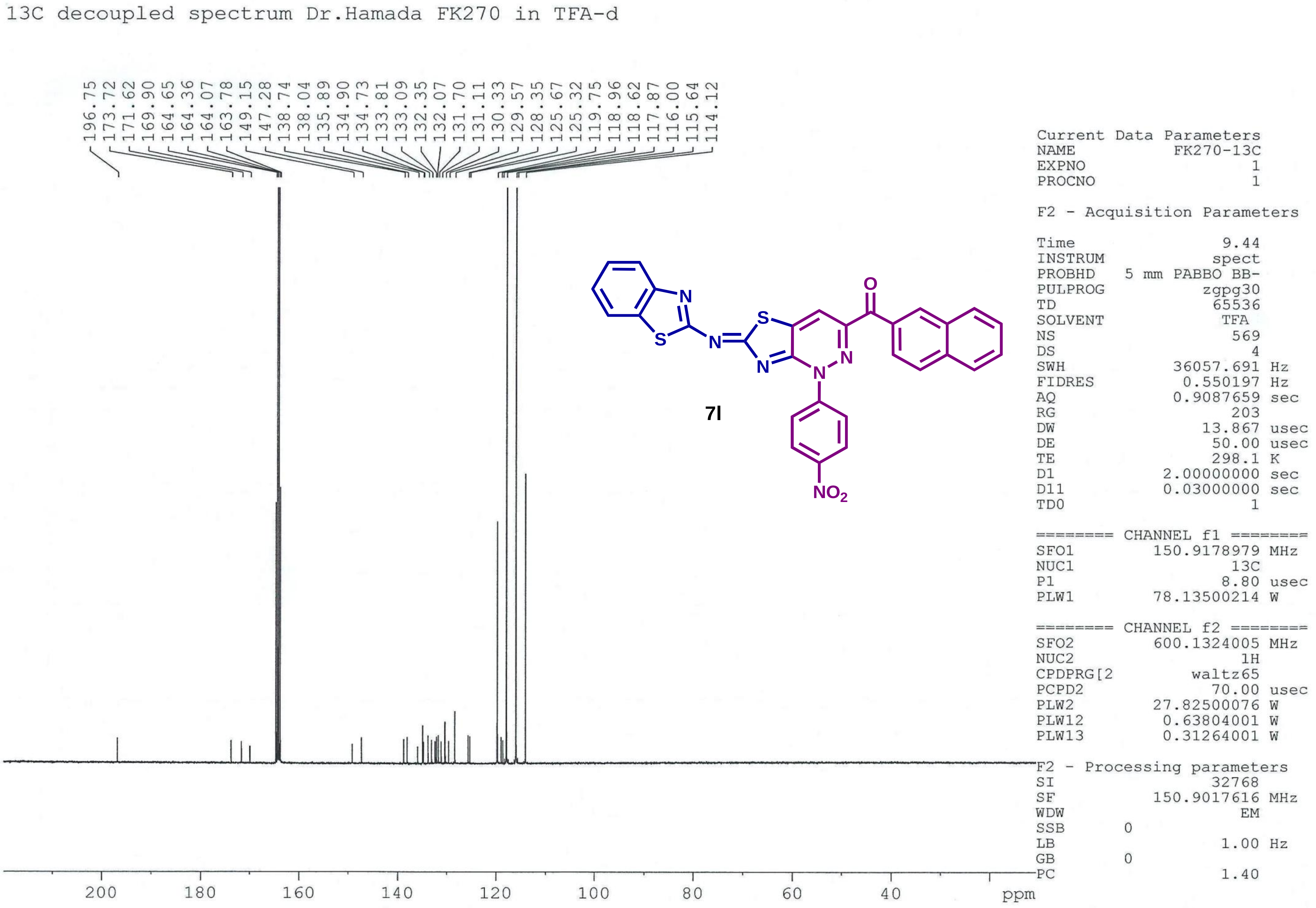

Figure S48. ^{13}C NMR Spectra (TFA-*d*, 150 MHz) for compound **7l**.

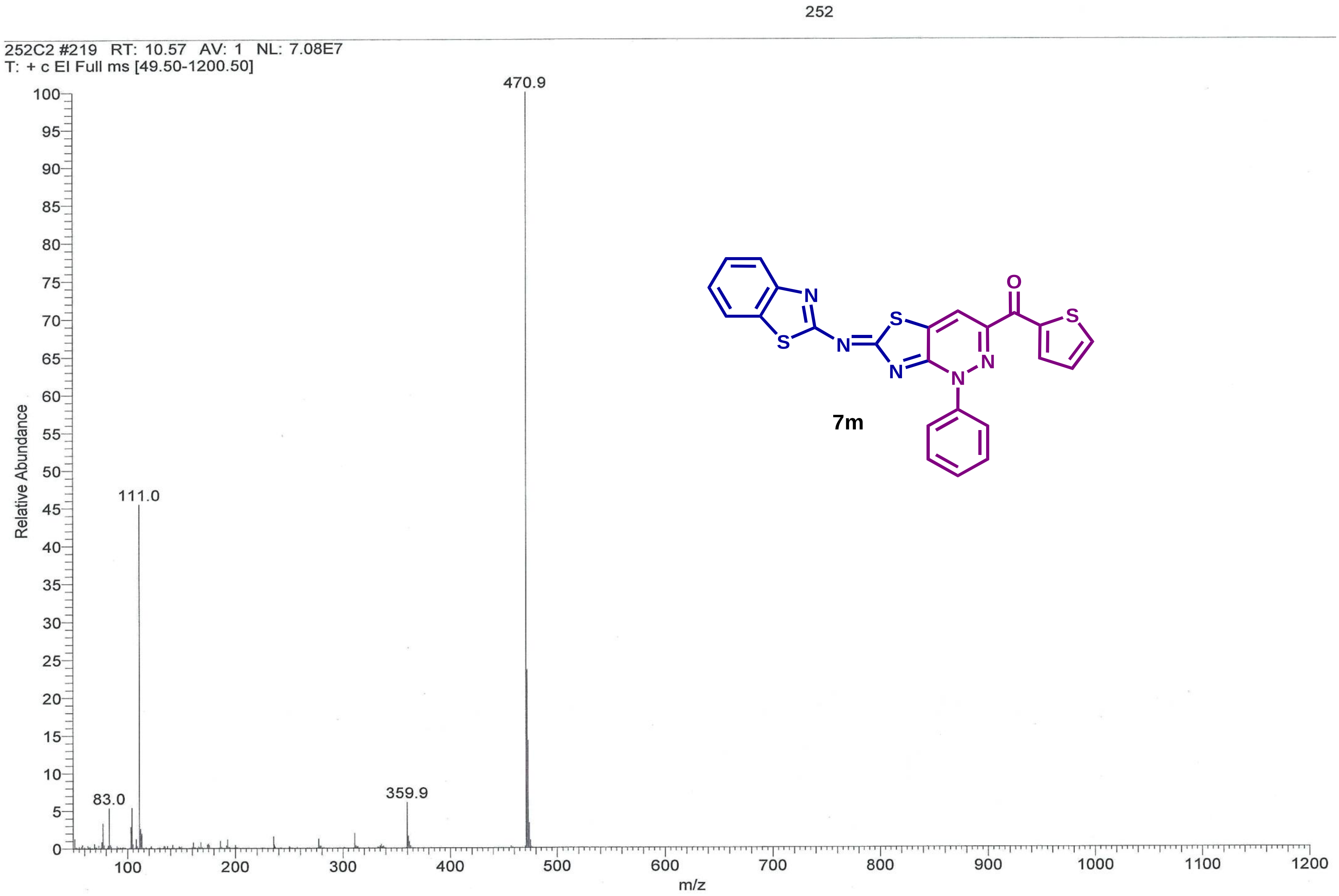

Figure S49. Mass Spectra for compound **7m.**

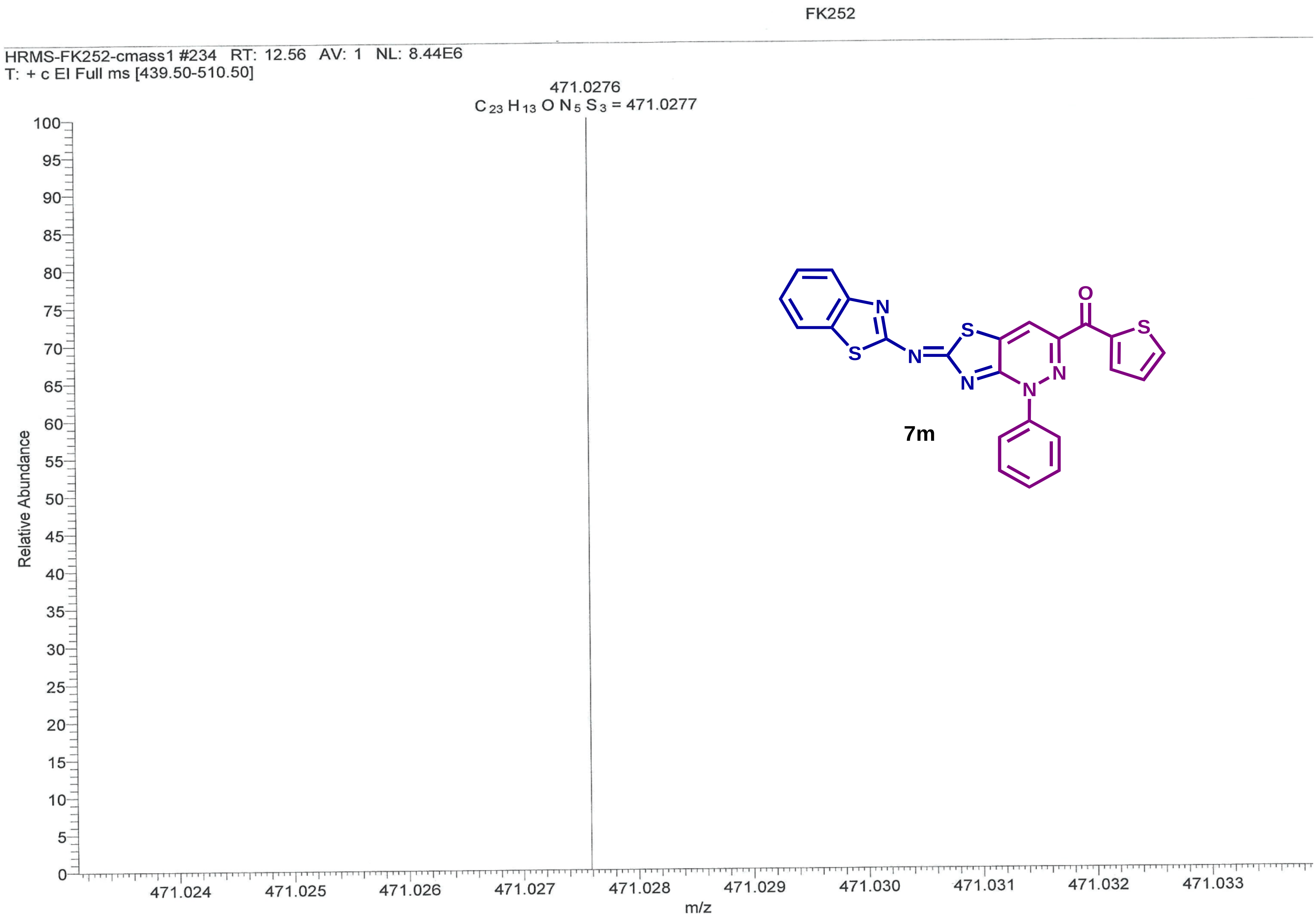

Figure S50. HRMS Spectra for compound **7m**.

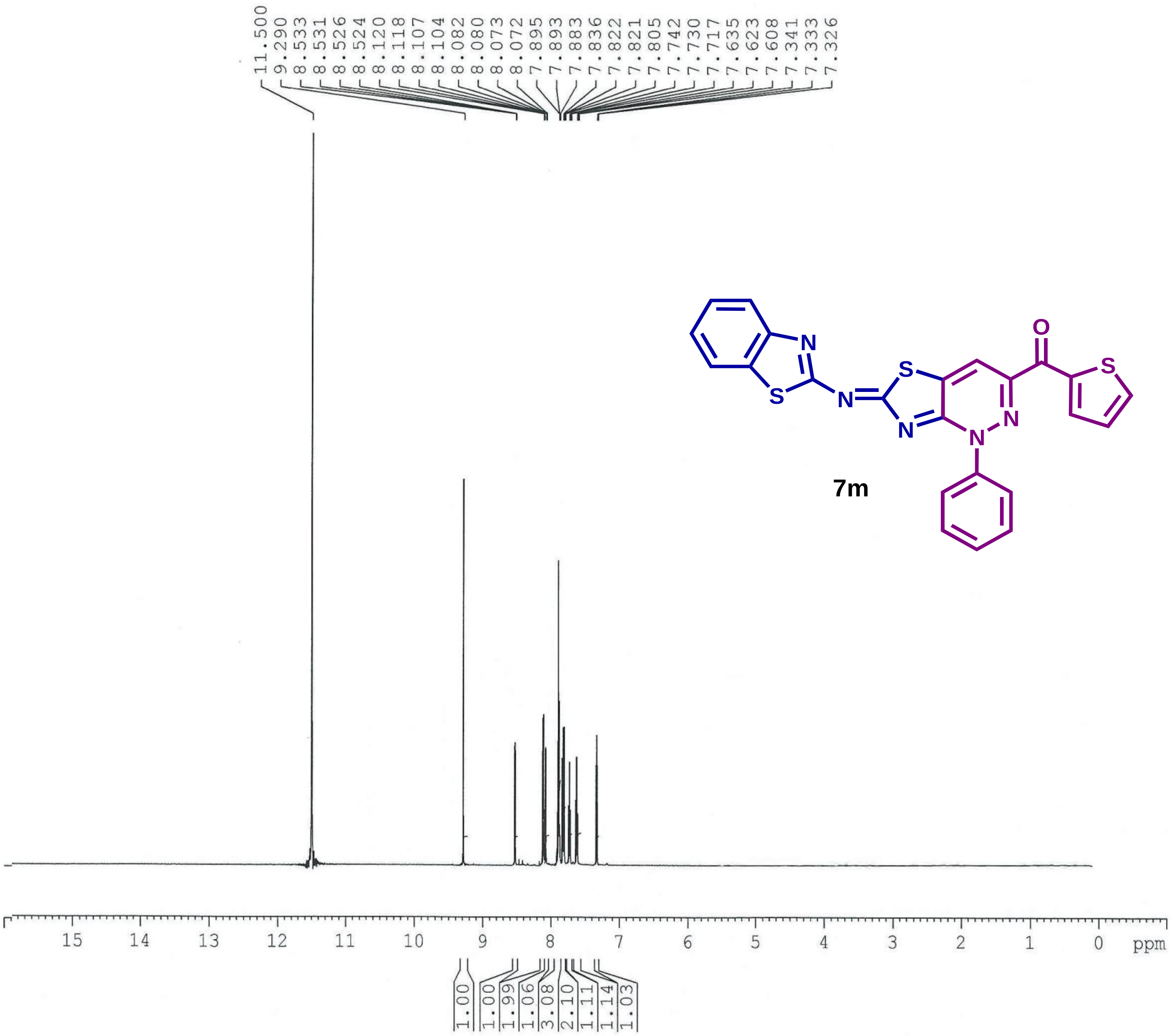

Current Data Parameters
NAME FK252C-1H
EXPNO 1
PROCNO 1

F2 - Acquisition Parameters

Time 14.09
INSTRUM spect
PROBHD 5 mm PABBO BB-
PULPROG zg30
TD 65536
SOLVENT TFA
NS 8
DS 2
SWH 12335.526 Hz
FIDRES 0.188225 Hz
AQ 2.6563926 sec
RG 203
DW 40.533 usec
DE 20.00 usec
TE 298.0 K
D1 1.00000000 sec
TD0 1

======== CHANNEL f1 ========
SFO1 600.1337060 MHz
NUC1 1H
P1 10.60 usec
PLW1 27.82500076 W

F2 - Processing parameters
SI 32768
SF 600.1274789 MHz
WDW EM
SSB 0
LB 0.30 Hz
GB 0
PC 1.00

Figure S51. ^{1}H NMR Spectra (TFA-*d*, 600 MHz) for compound **7m**.

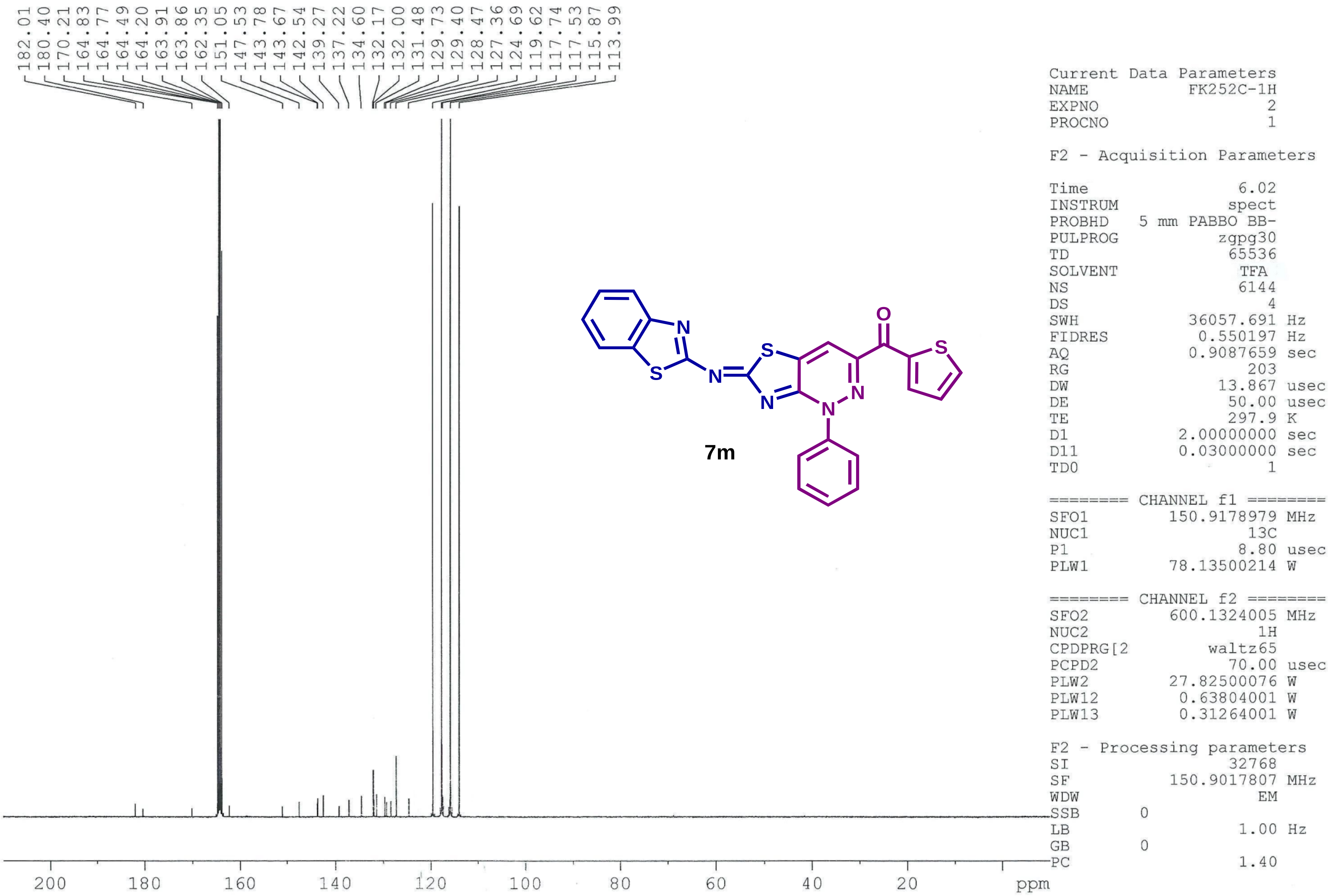

Figure S52. ^{13}C NMR Spectra (TFA-*d*, 150 MHz) for compound **7m**.

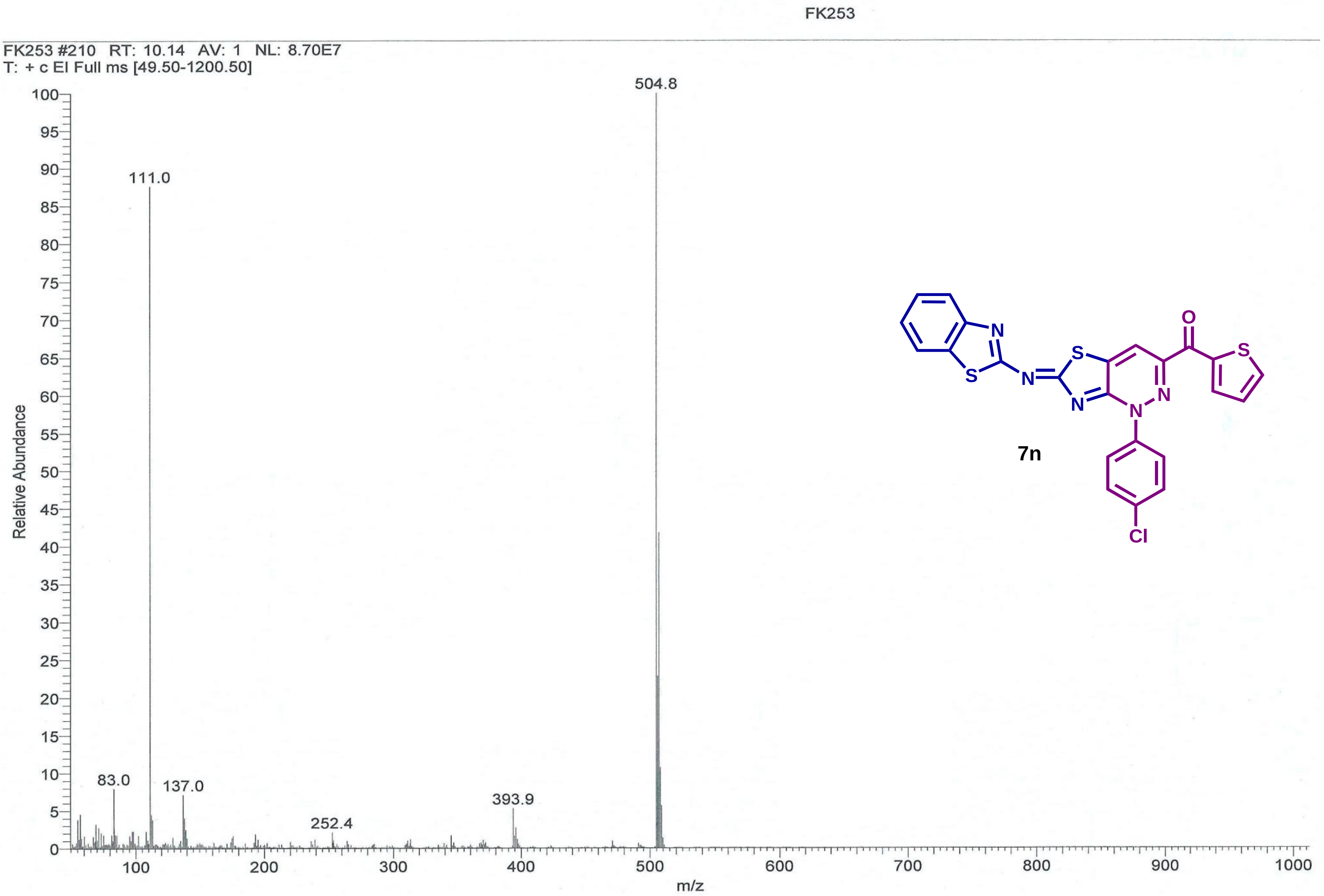

Figure S53. Mass Spectra for compound **7n**

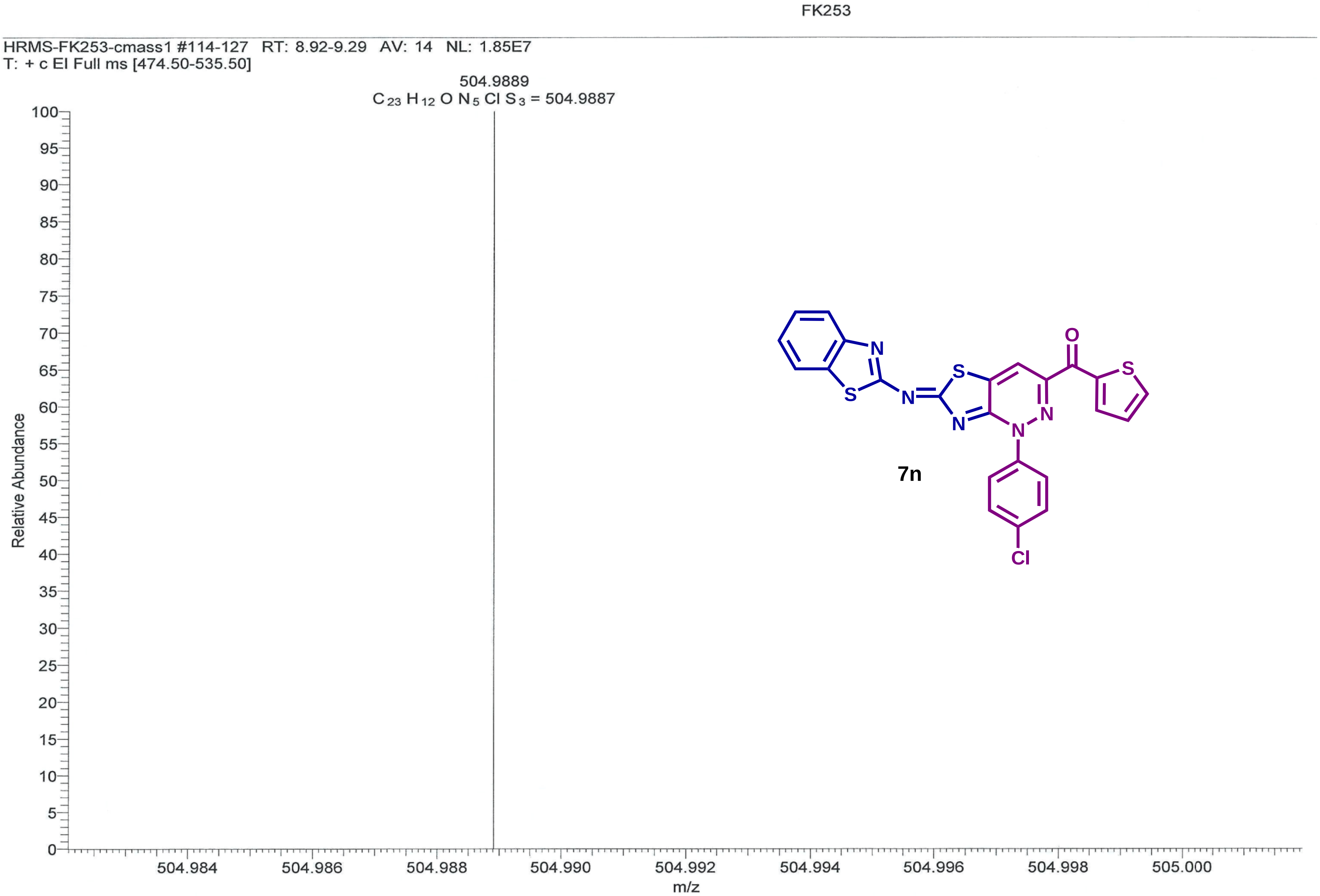

Figure S54. HRMS Spectra for compound **7n**.

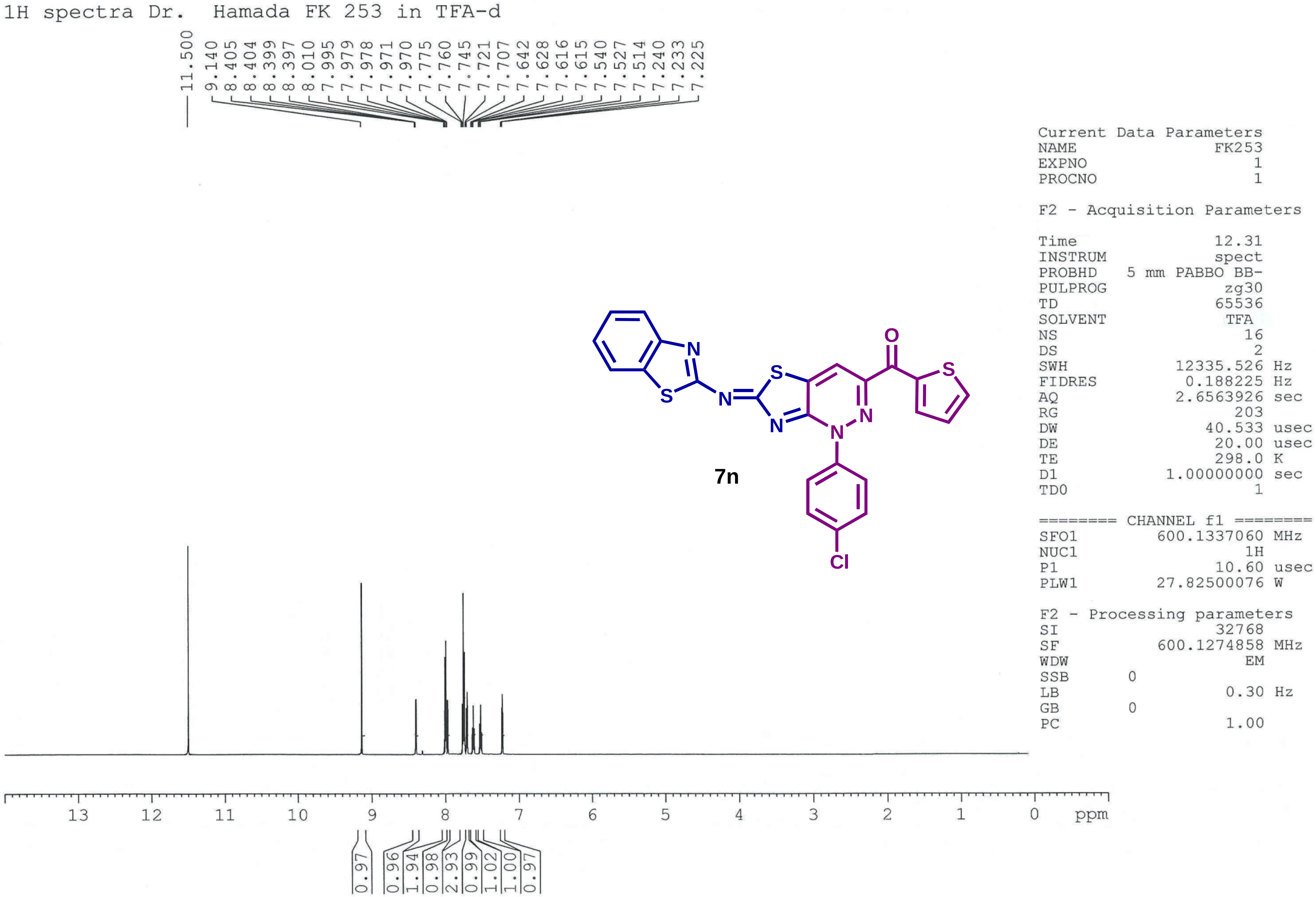

Figure S55. ^{1}H NMR Spectra (TFA-*d*, 600 MHz) for compound **7n**

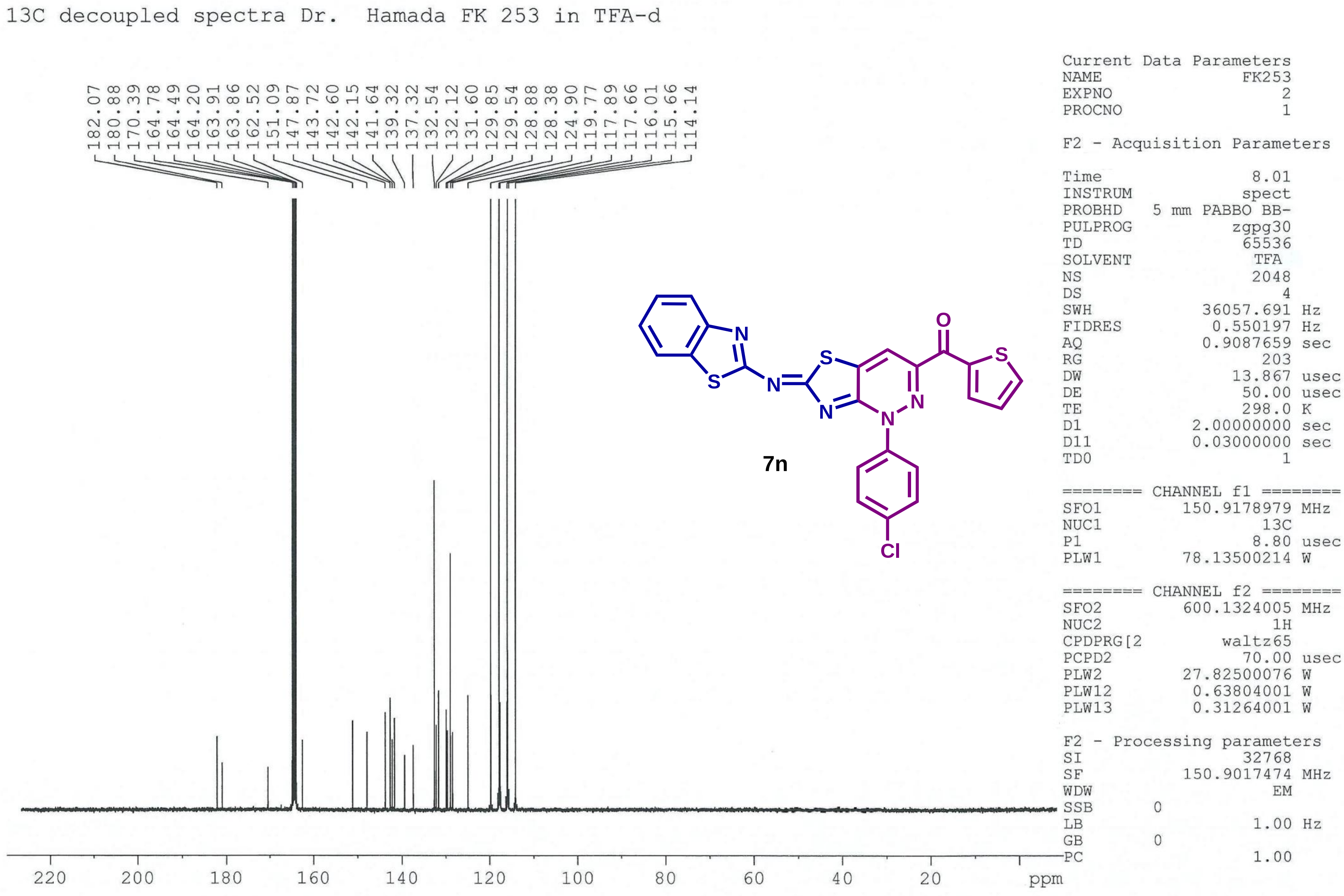

Current Data Parameters
NAME FK253
EXPNO 2
PROCNO 1

F2 - Acquisition Parameters

Time 8.01
INSTRUM spect
PROBHD 5 mm PABBO BB-
PULPROG zgpg30
TD 65536
SOLVENT TFA
NS 2048
DS 4
SWH 36057.691 Hz
FIDRES 0.550197 Hz
AQ 0.9087659 sec
RG 203
DW 13.867 usec
DE 50.00 usec
TE 298.0 K
D1 2.00000000 sec
D11 0.03000000 sec
TD0 1

======== CHANNEL f1 ========
SFO1 150.9178979 MHz
NUC1 13C
P1 8.80 usec
PLW1 78.13500214 W

======== CHANNEL f2 ========
SFO2 600.1324005 MHz
NUC2 1H
CPDPRG[2 waltz65
PCPD2 70.00 usec
PLW2 27.82500076 W
PLW12 0.63804001 W
PLW13 0.31264001 W

F2 - Processing parameters
SI 32768
SF 150.9017474 MHz
WDW EM
SSB 0
LB 1.00 Hz
GB 0
PC 1.00

Figure S56. ^{13}C NMR Spectra (TFA-*d*, 150 MHz) for compound **7n**.

FK259

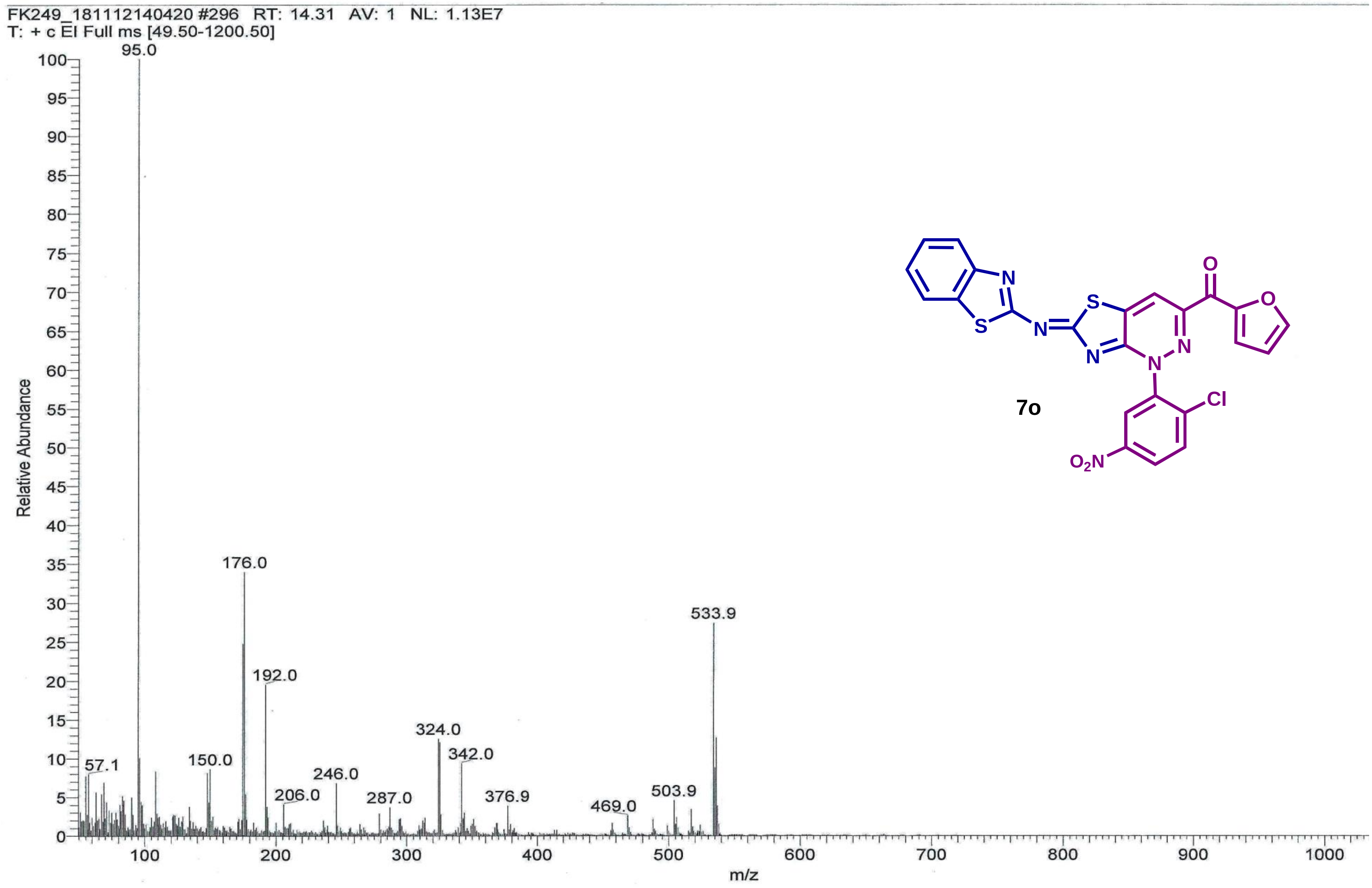

Figure S57. Mass Spectra for compound **7o**

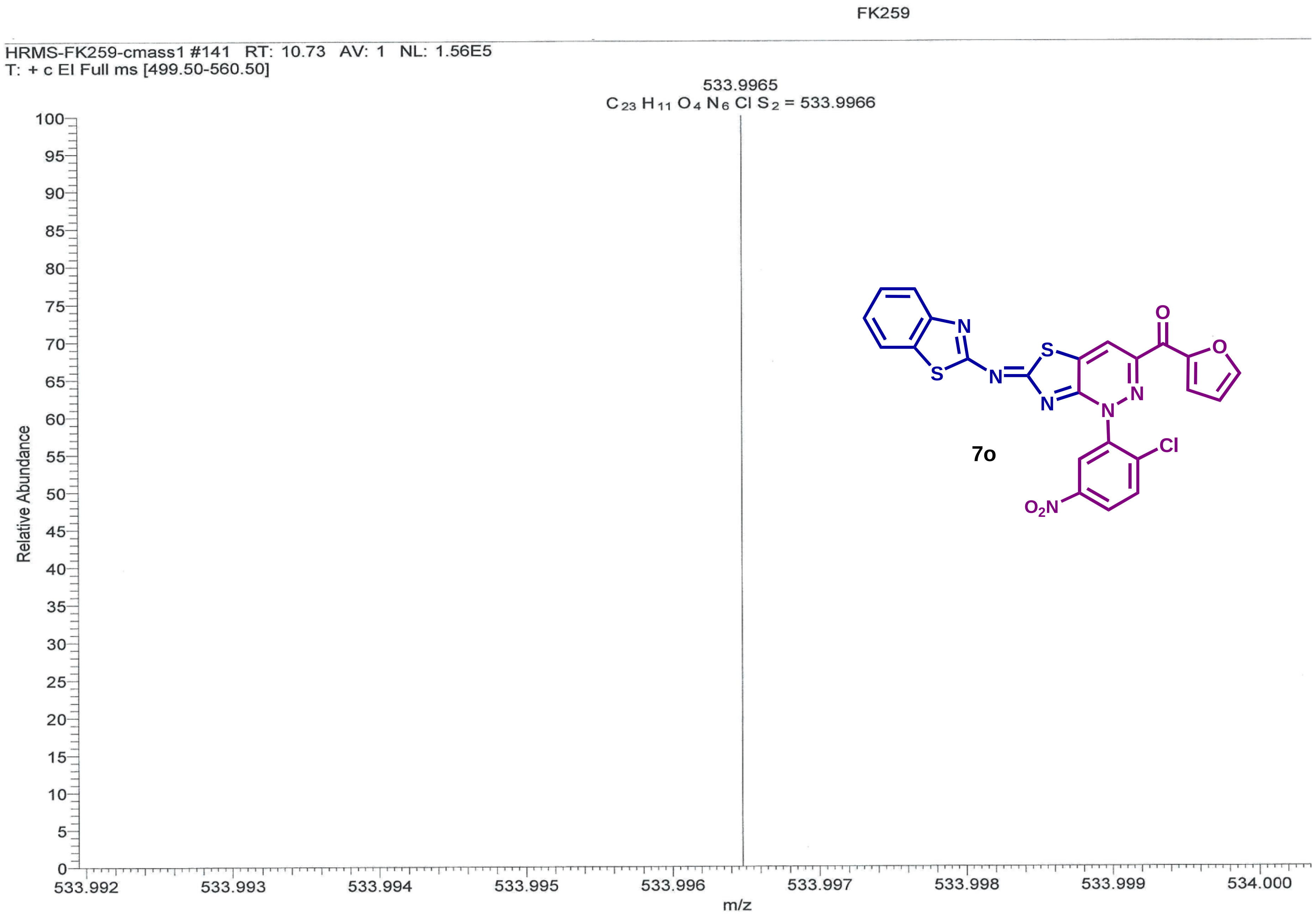

Figure S58. HRMS Spectra for compound **7o**.

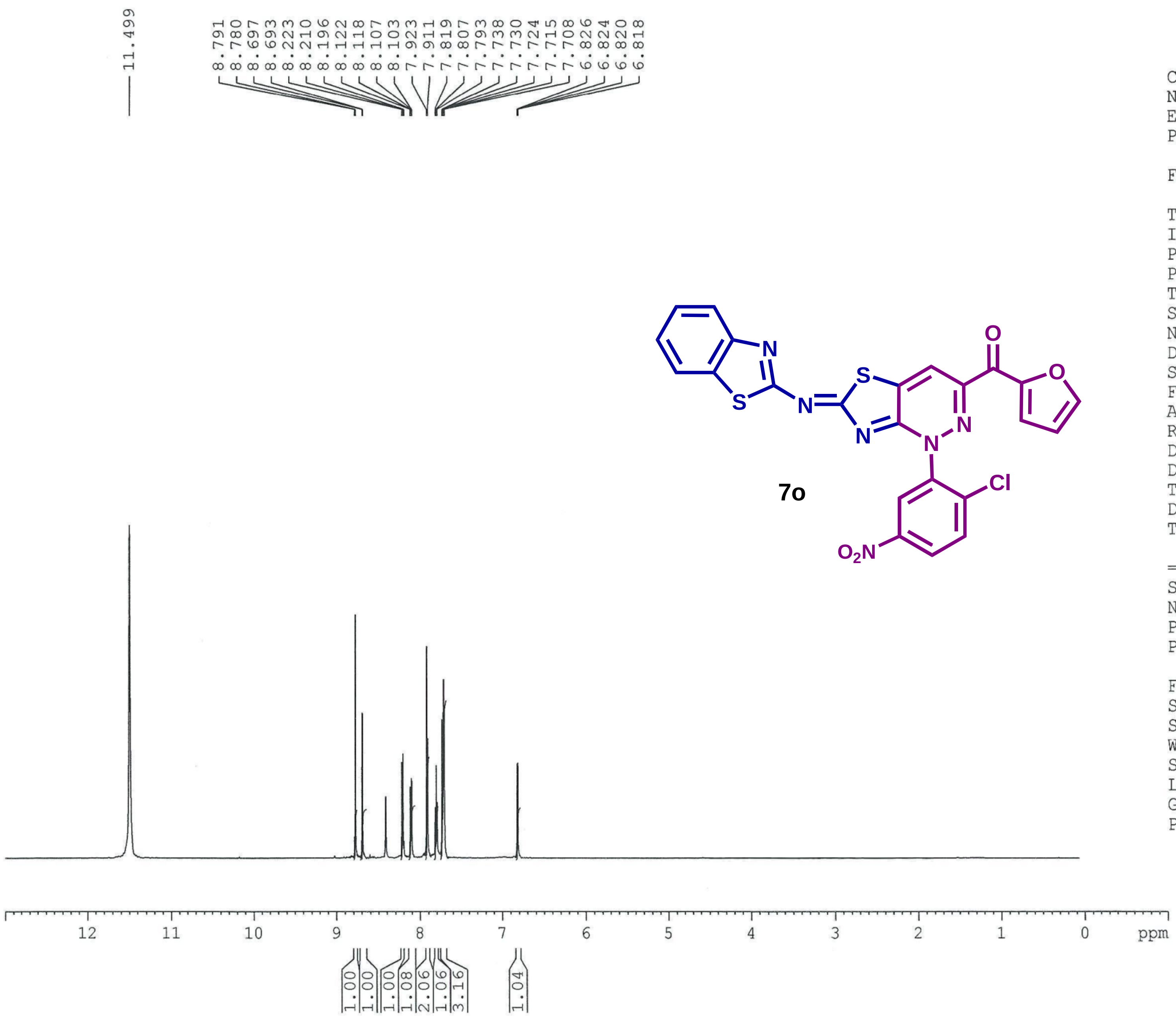

Current Data Parameters
NAME FVP259
EXPNO 1
PROCNO 1

F2 - Acquisition Parameters

Time	10.52
INSTRUM	spect
PROBHD	5 mm PABBO BB-
PULPROG	zg30
TD	65536
SOLVENT	TFA
NS	16
DS	2
SWH	12335.526 Hz
FIDRES	0.188225 Hz
AQ	2.6563926 sec
RG	128
DW	40.533 usec
DE	20.00 usec
TE	298.0 K
D1	1.00000000 sec
TD0	1

======== CHANNEL f1 ========

SFO1	600.1337060 MHz
NUC1	1H
P1	10.60 usec
PLW1	27.82500076 W

F2 - Processing parameters

SI	32768
SF	600.1274951 MHz
WDW	EM
SSB	0
LB	0.30 Hz
GB	0
PC	1.00

Figure S59. ^{1}H NMR Spectra (TFA-*d*, 600 MHz) for compound **7o.**

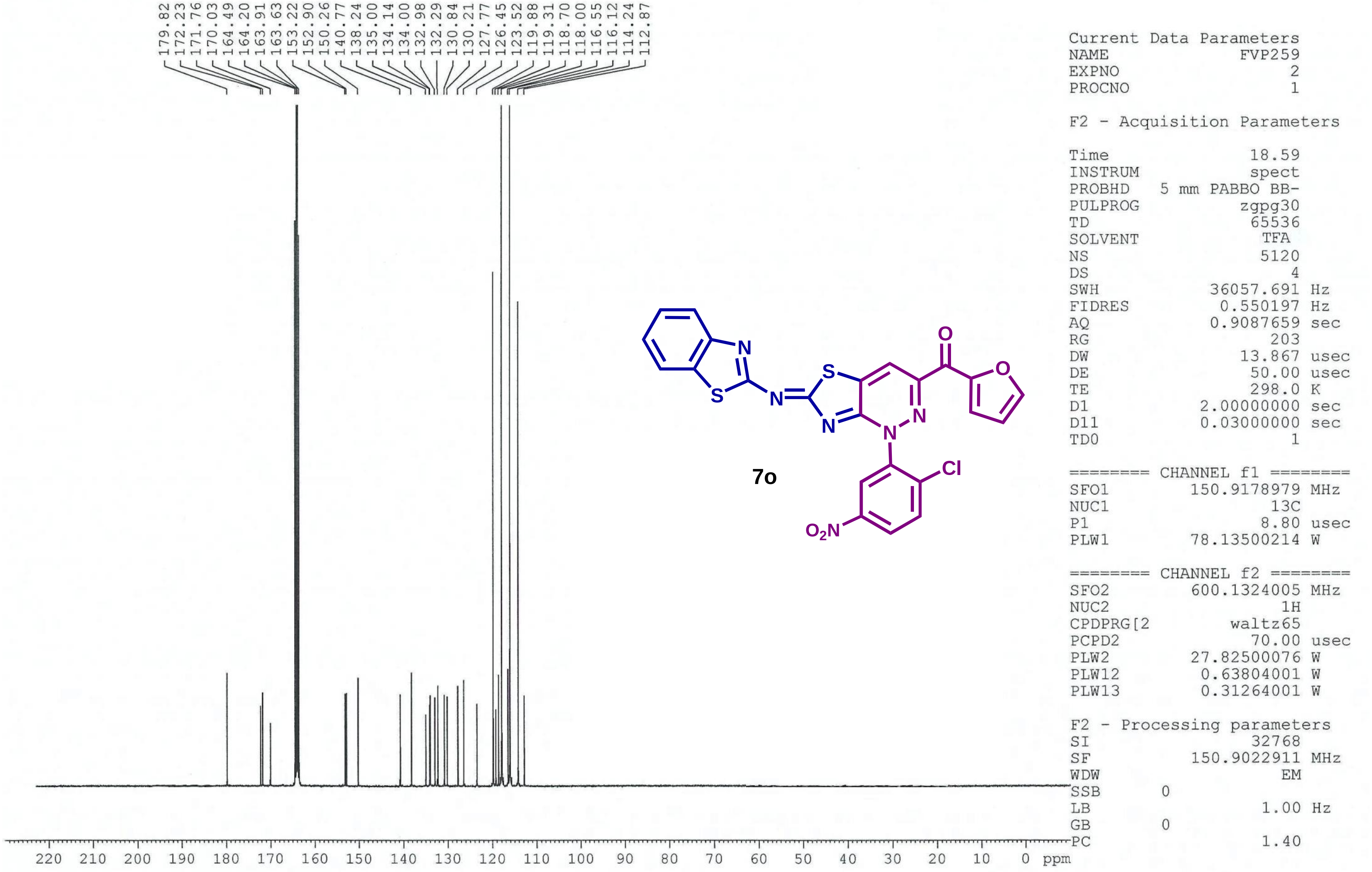

Figure S60. ^{13}C NMR Spectra (TFA-*d*, 150 MHz) for compound **7o.**

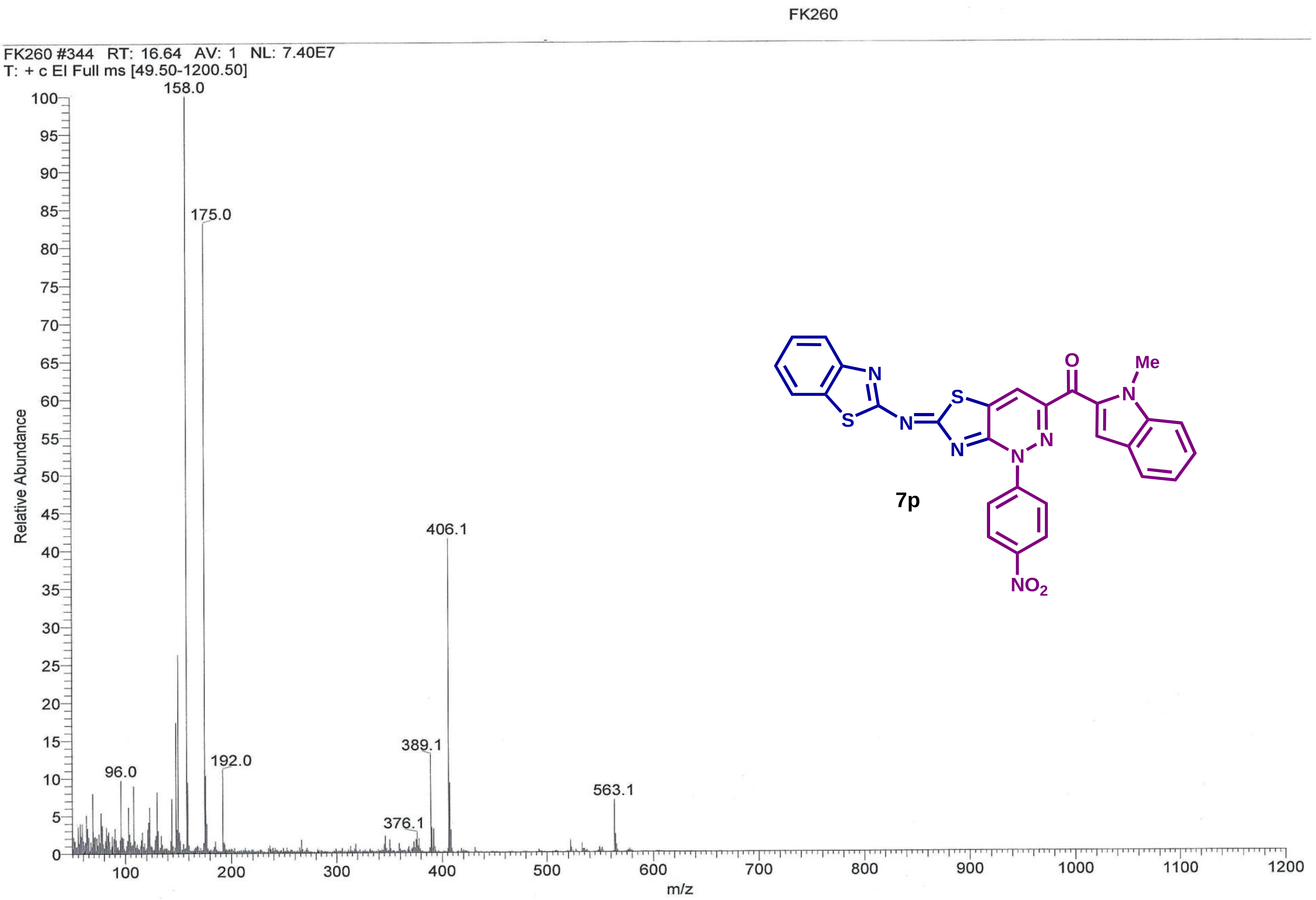

Figure S61. Mass Spectra for compound **7p.**

FK260

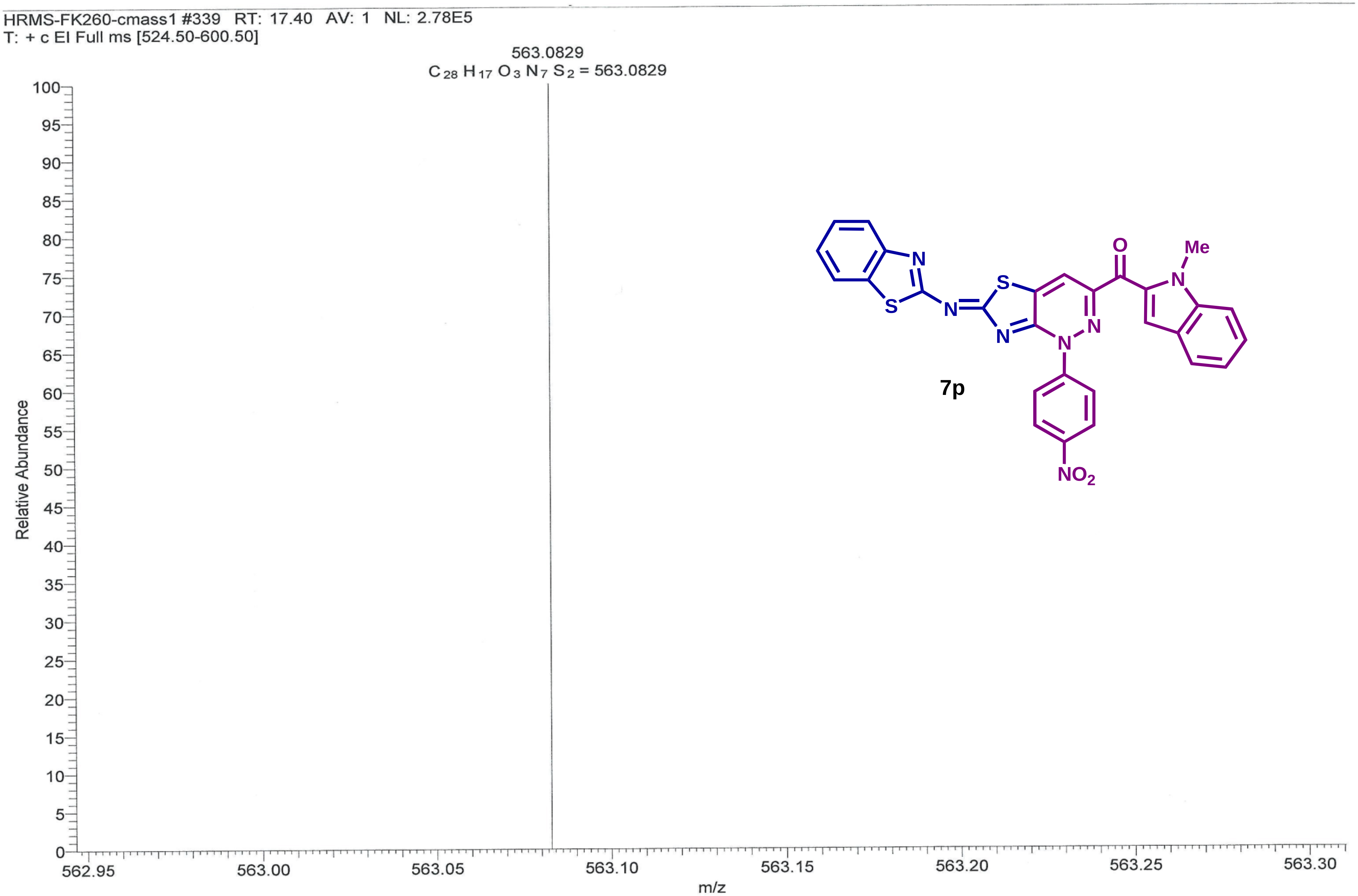

7p

Figure S62. HRMS Spectra for compound **7p**.

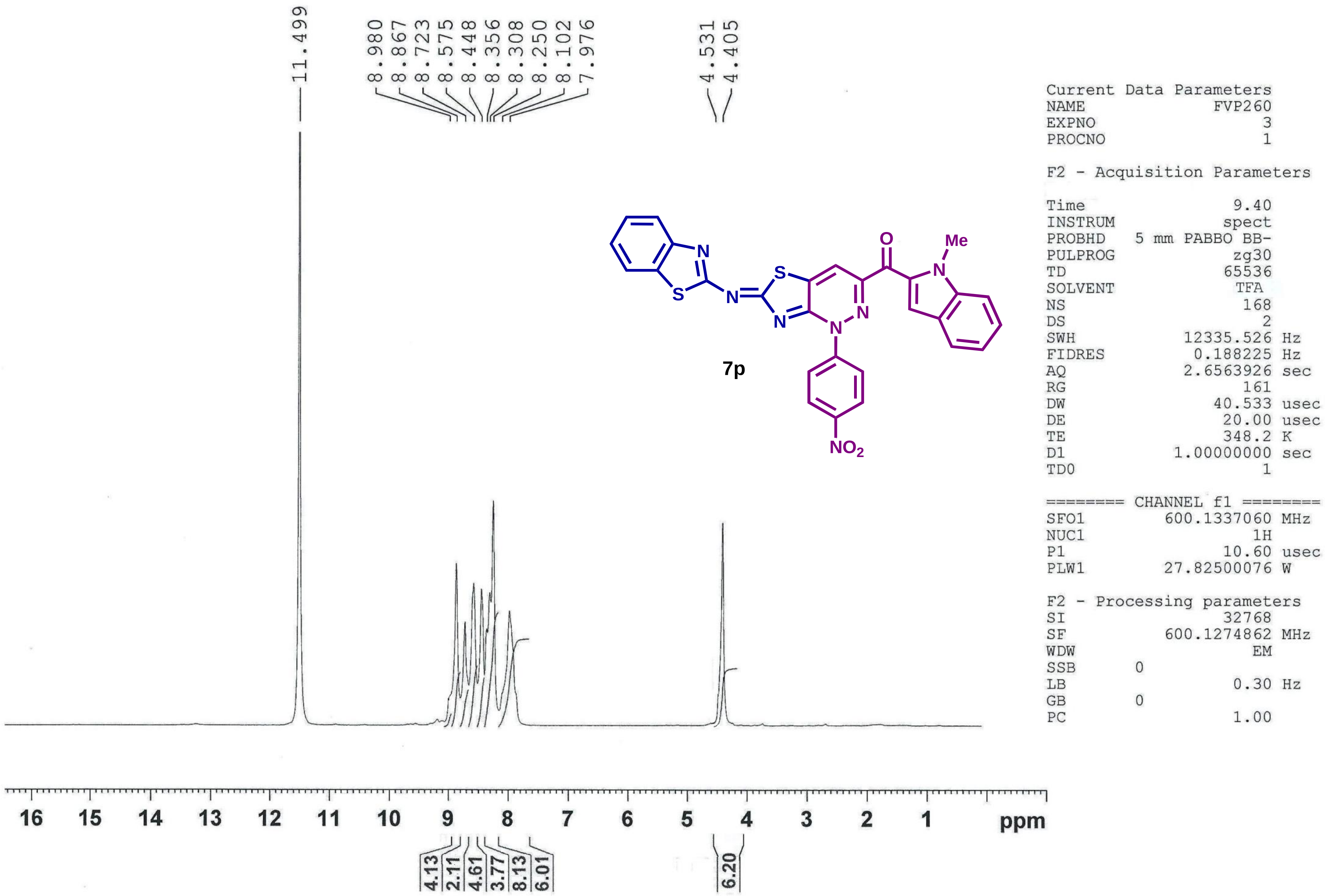

7p

Figure S63. ^{1}H NMR Spectra (TFA-*d*, 600 MHz) for compound **7p.**

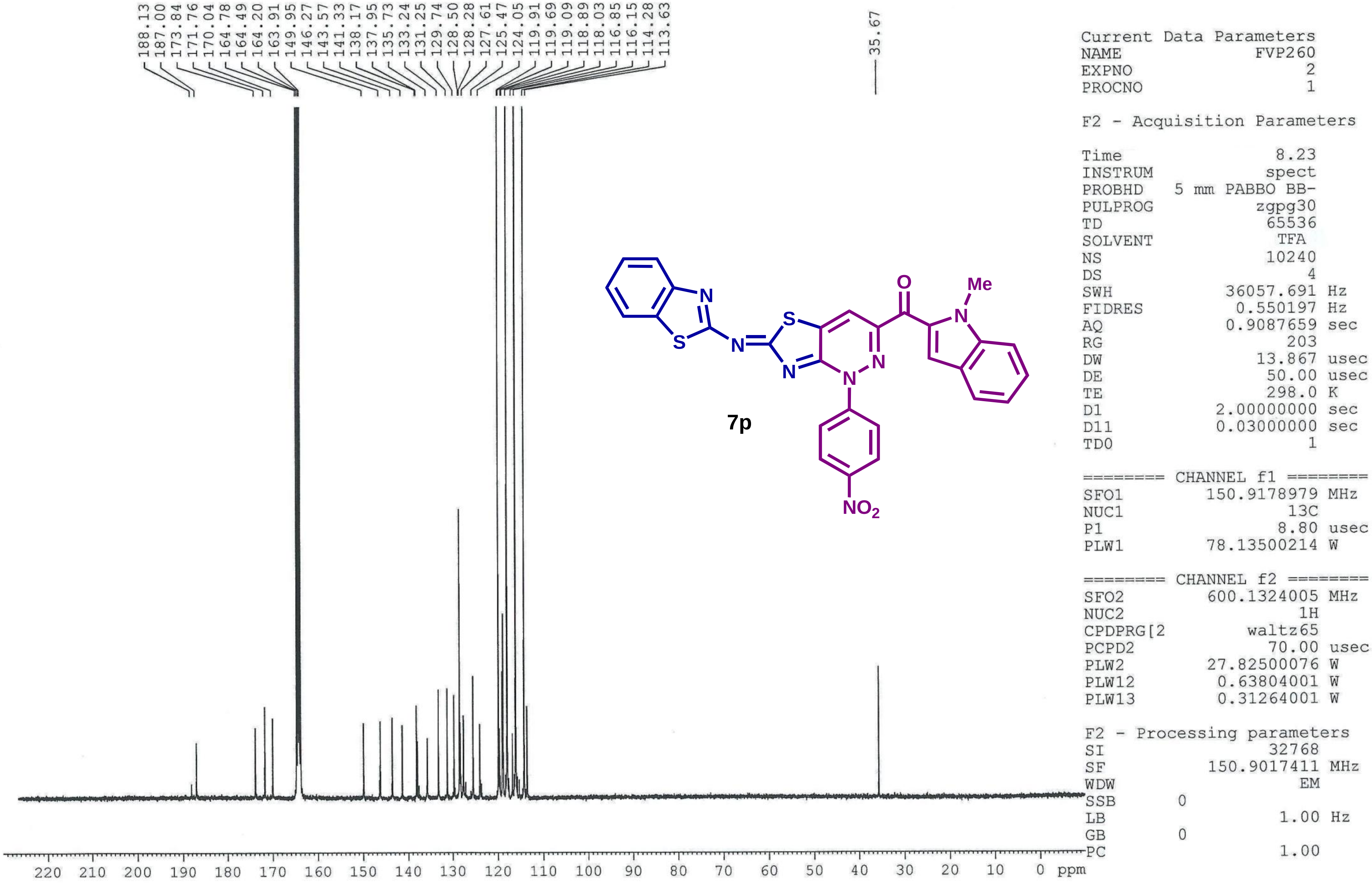

Figure S64. ^{13}C NMR Spectra (TFA-*d*, 150 MHz) for compound **7p.**

FK245

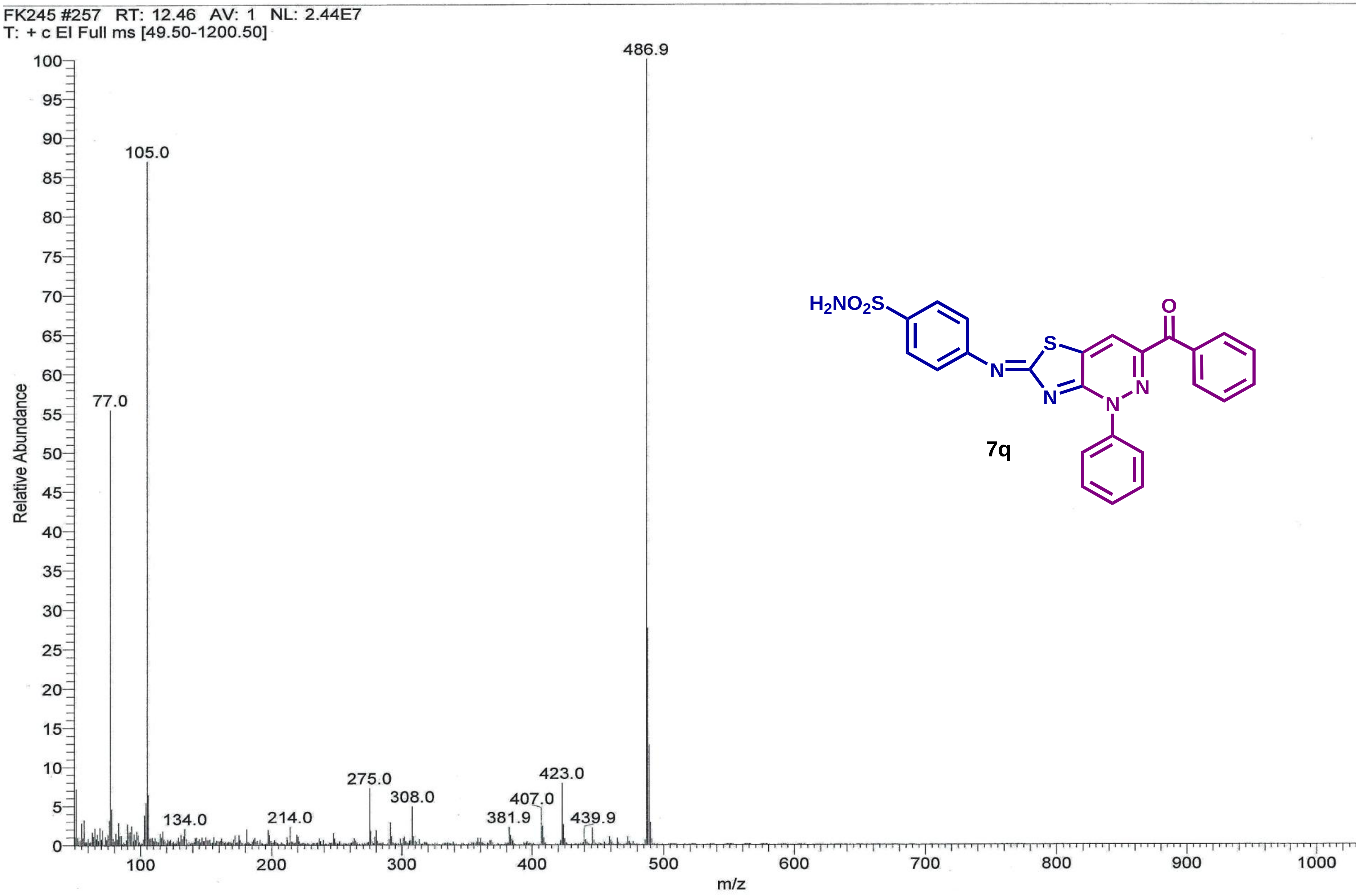

7q

Figure S65. Mass Spectra for compound **7q.**

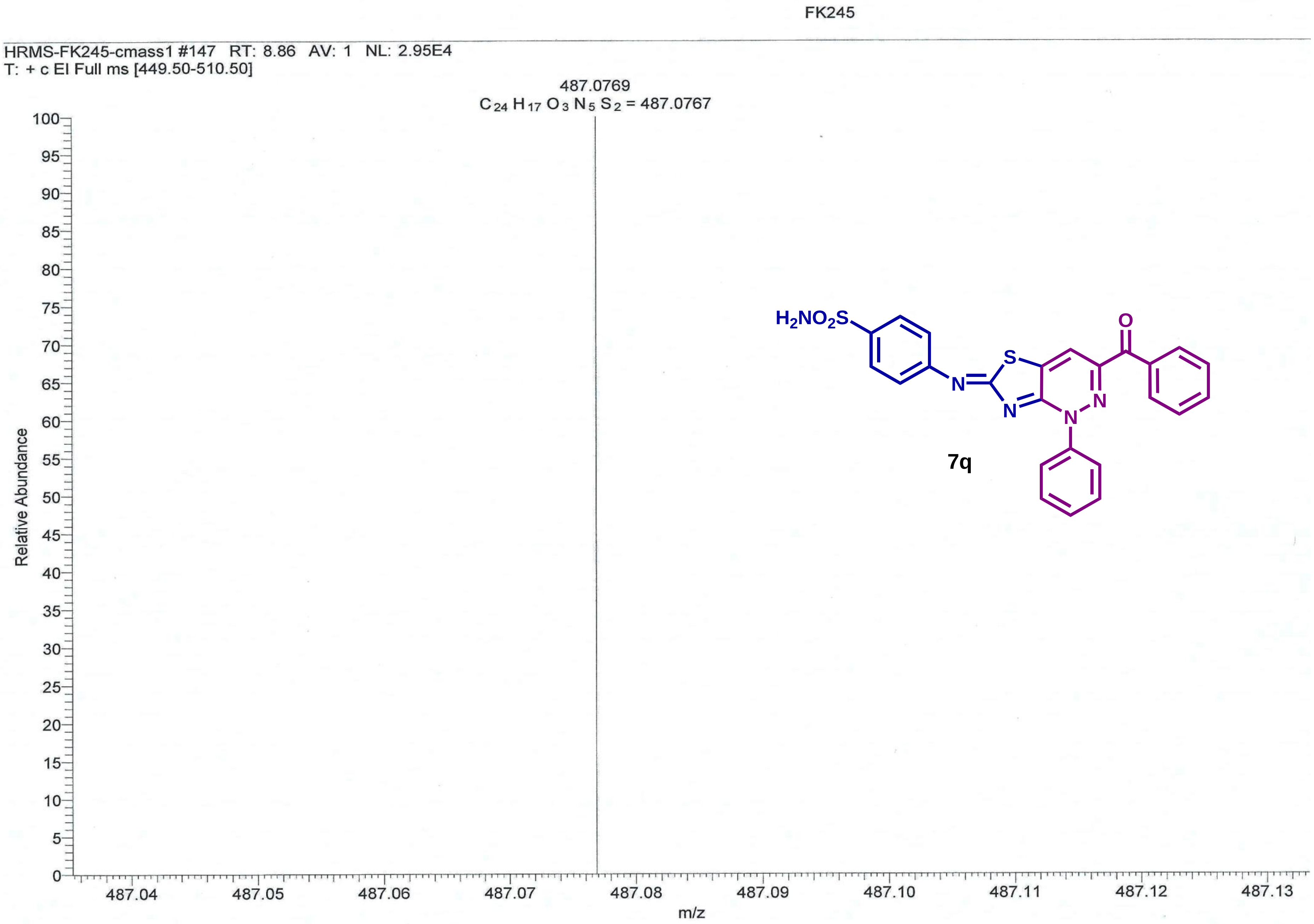

Figure S66. HRMS Spectra for compound **7q**.

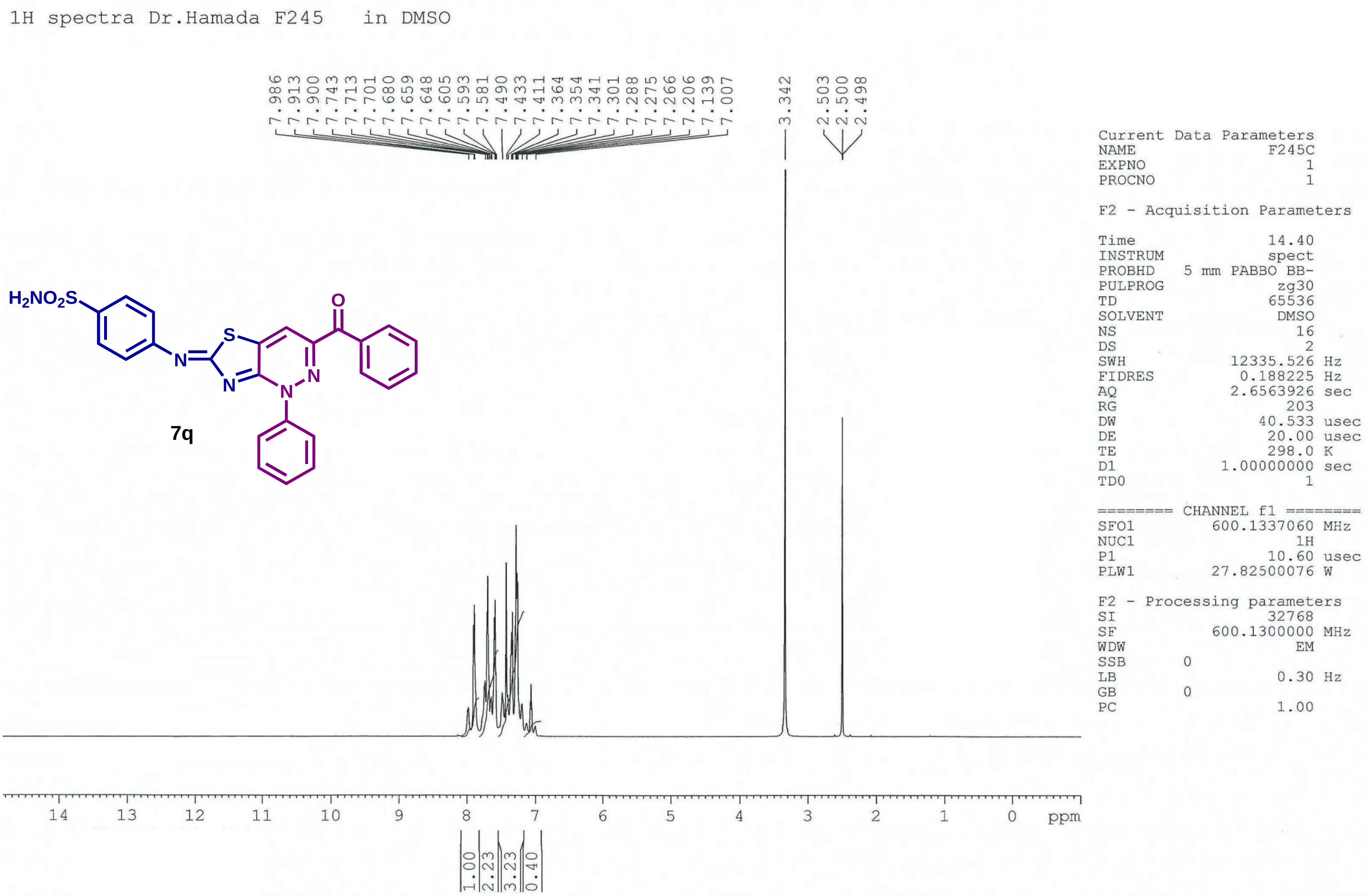

Figure S67. ^{1}H NMR Spectra (DMSO-d_6, 600 MHz) for compound **7q**.

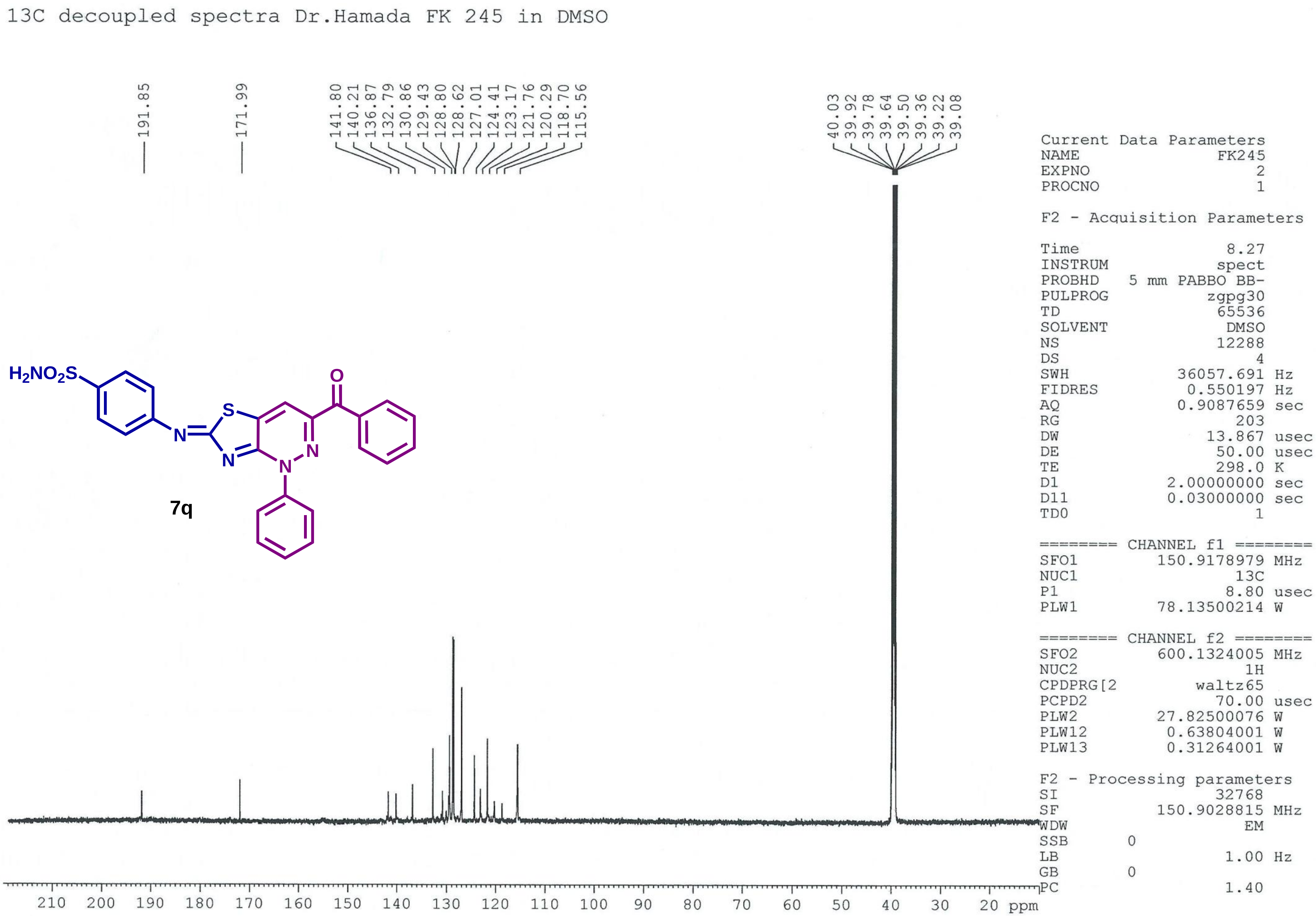

Figure S68. ^{13}C NMR Spectra (DMSO-d_6, 150 MHz) for compound **7q**.

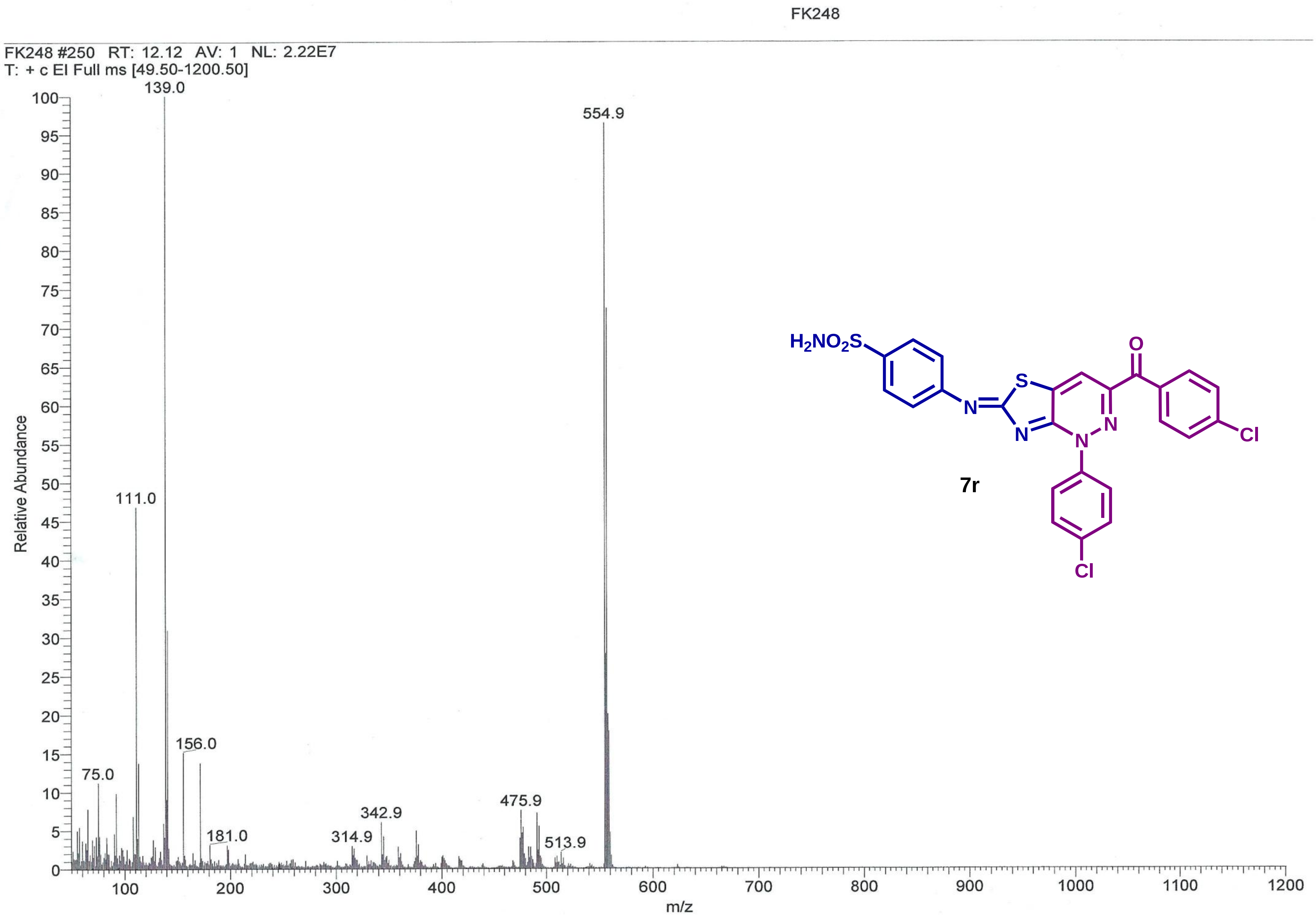

Figure S69. Mass Spectra for compound **7r**.

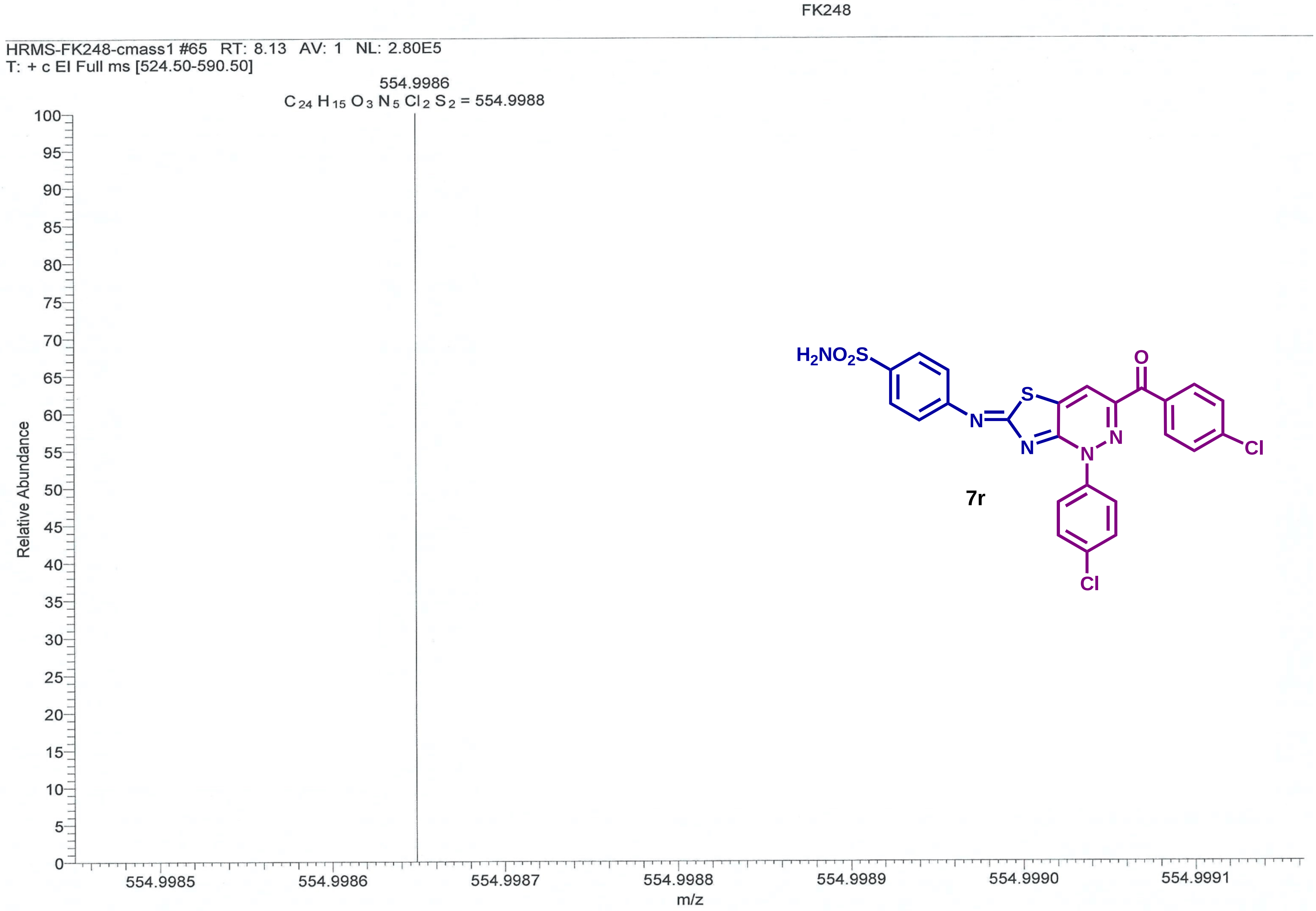

Figure S70. HRMS Spectra for compound **7r**.

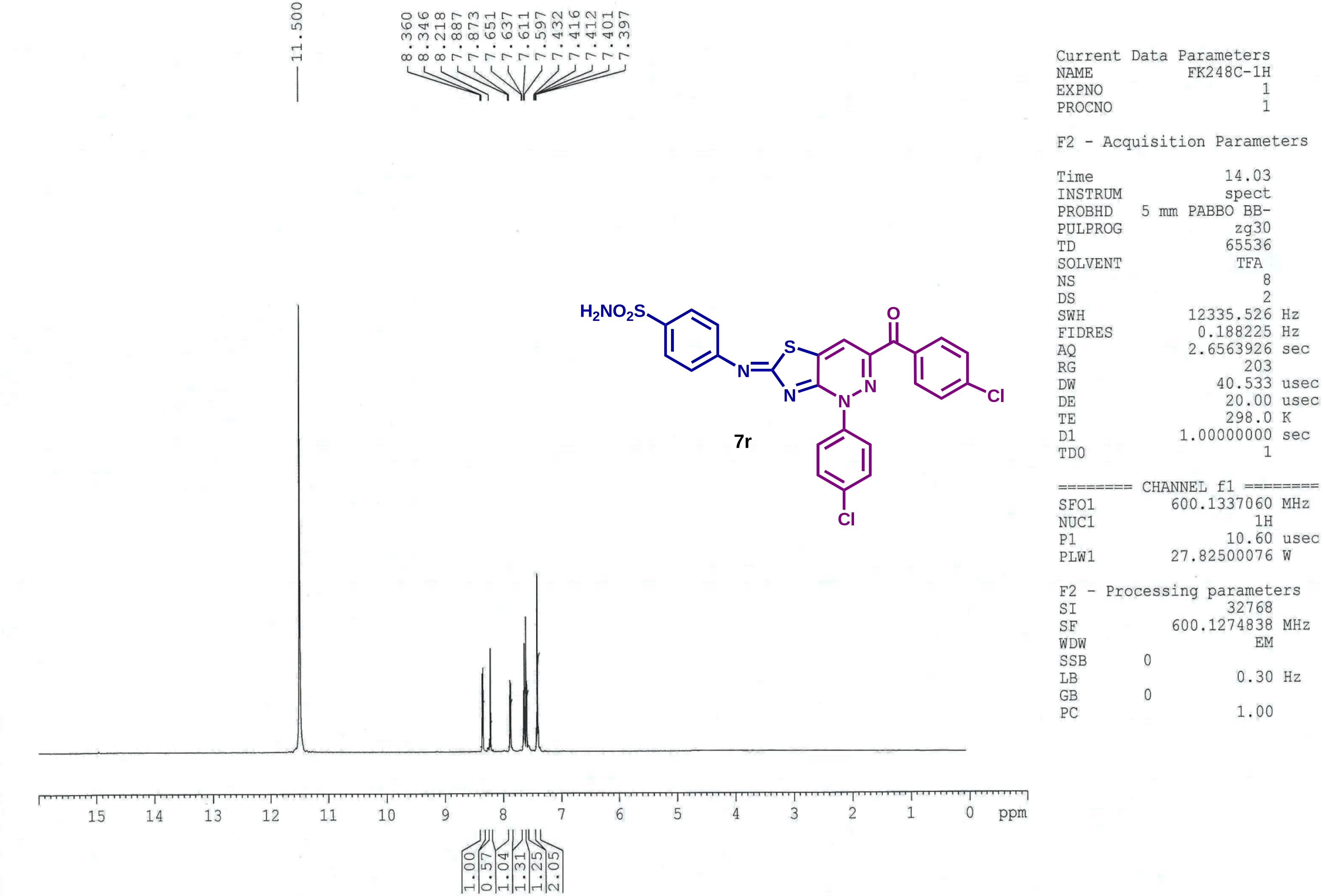

Figure S71. ^{1}H NMR Spectra (TFA-*d*, 600 MHz) for compound **7r**.

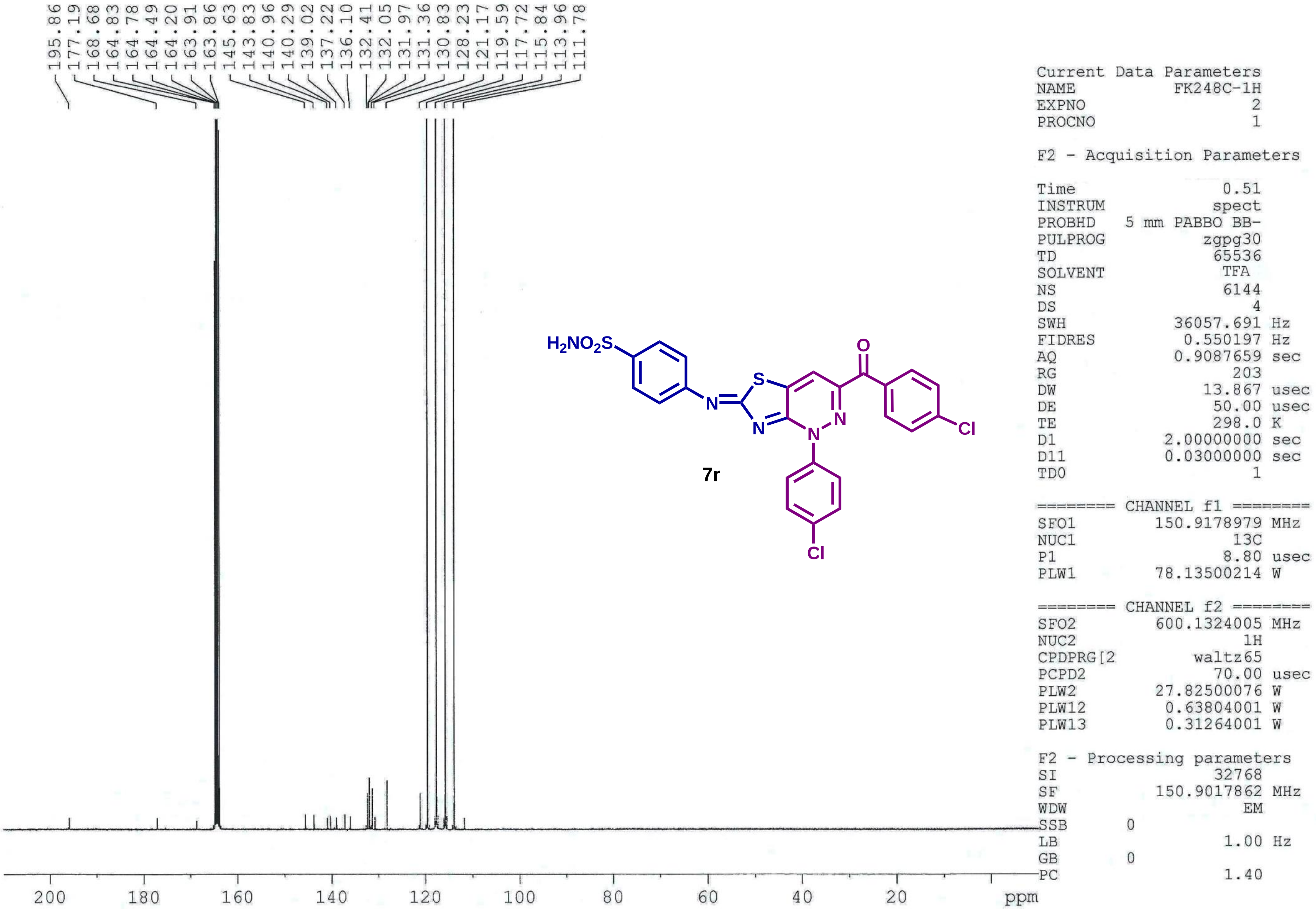

Figure S72. ^{13}C NMR Spectra (TFA-*d*, 150 MHz) for compound **7r**.

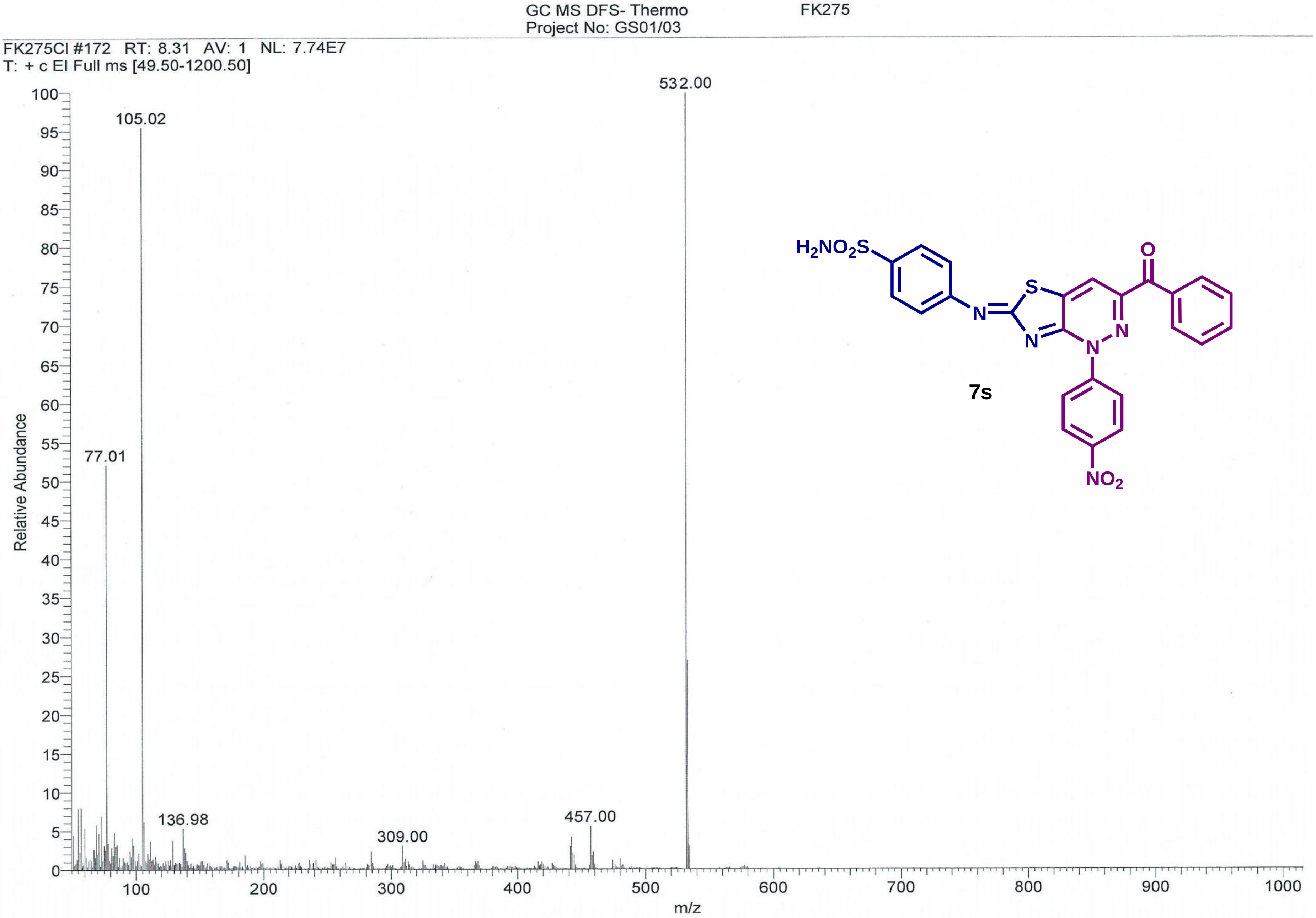

Figure S73. Mass Spectra for compound **7s**.

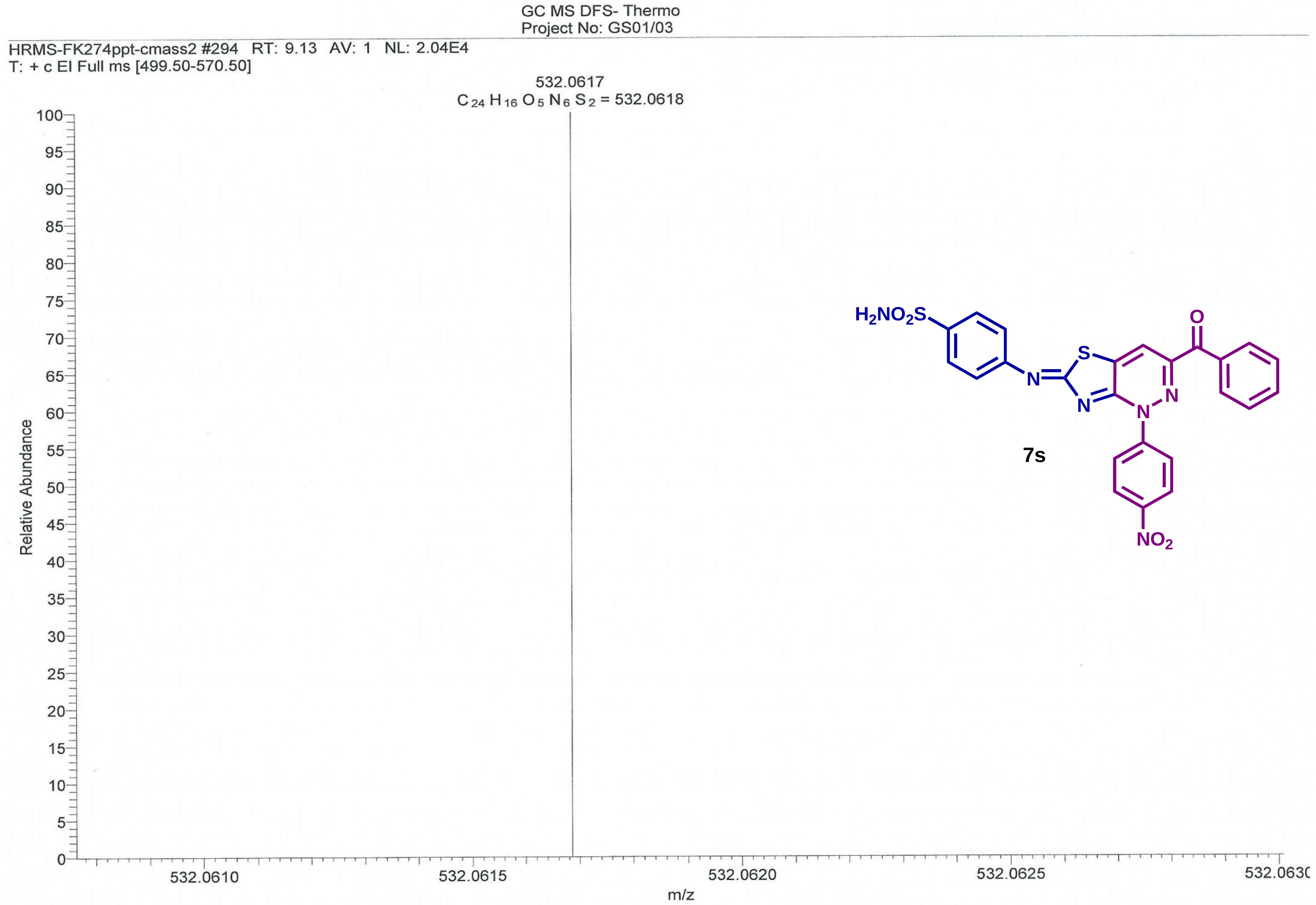

Figure S74. HRMS Spectra for compound **7s**.

1H spectra Dr.Hamada FK 275 in TFA-d

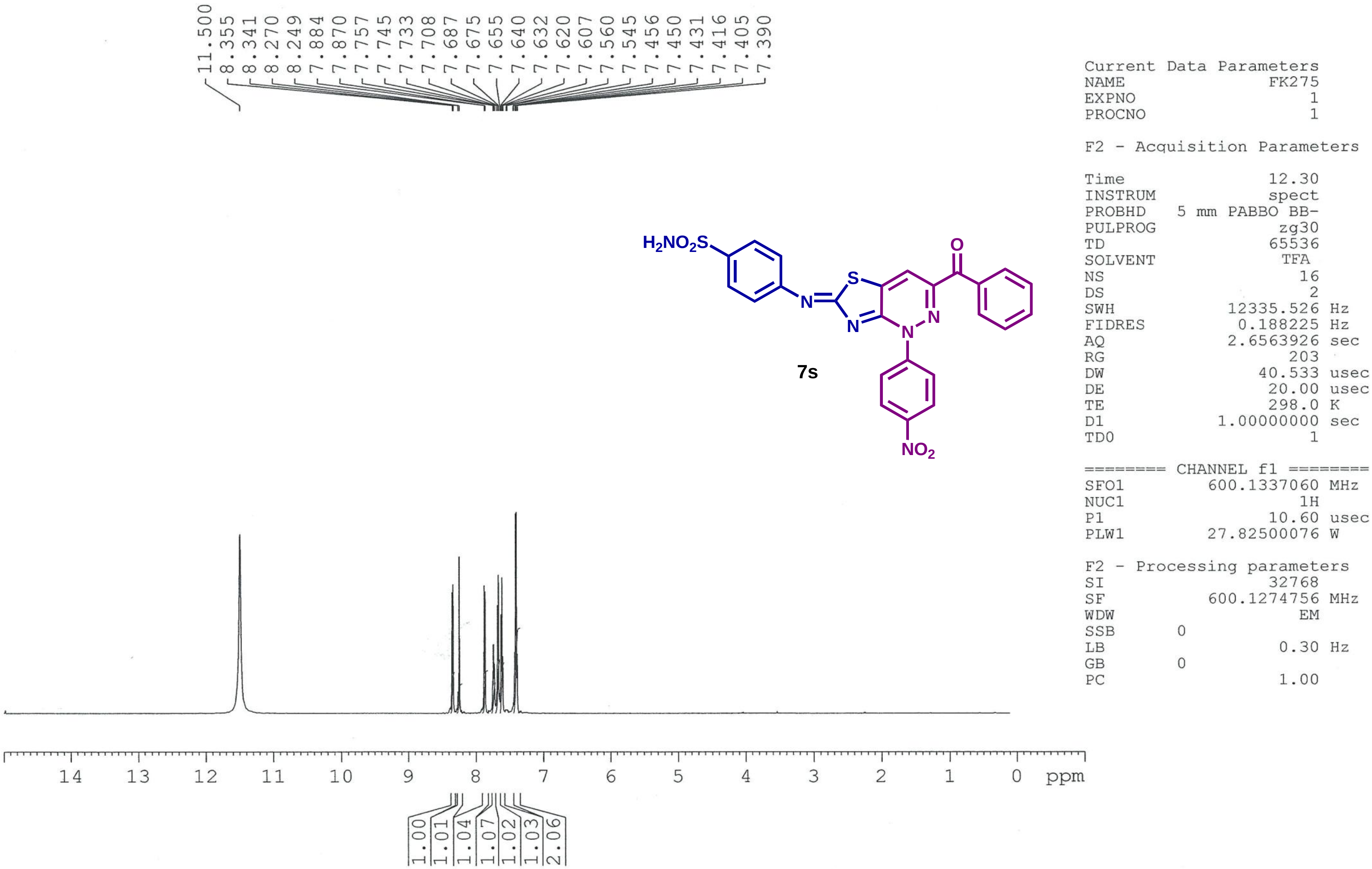

7s

Current Data Parameters
NAME FK275
EXPNO 1
PROCNO 1

F2 - Acquisition Parameters

Time	12.30
INSTRUM	spect
PROBHD	5 mm PABBO BB-
PULPROG	zg30
TD	65536
SOLVENT	TFA
NS	16
DS	2
SWH	12335.526 Hz
FIDRES	0.188225 Hz
AQ	2.6563926 sec
RG	203
DW	40.533 usec
DE	20.00 usec
TE	298.0 K
D1	1.00000000 sec
TD0	1

======== CHANNEL f1 ========

SFO1	600.1337060 MHz
NUC1	1H
P1	10.60 usec
PLW1	27.82500076 W

F2 - Processing parameters

SI	32768
SF	600.1274756 MHz
WDW	EM
SSB	0
LB	0.30 Hz
GB	0
PC	1.00

Figure S75. ^{1}H NMR Spectra (TFA-*d*, 600 MHz) for compound **7s**.

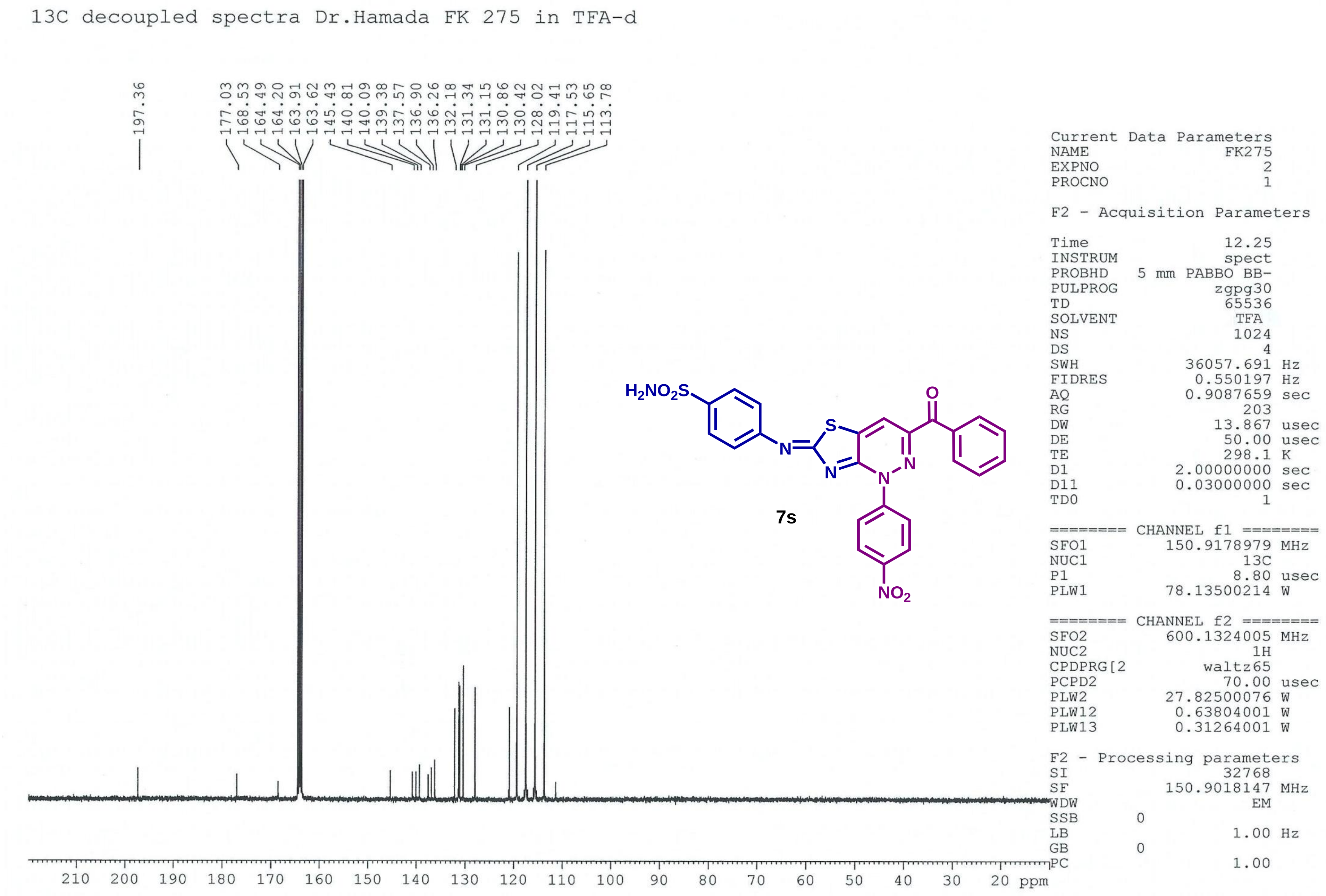

Figure S76. ^{13}C NMR Spectra (TFA-*d*, 150 MHz) for compound **7s**.

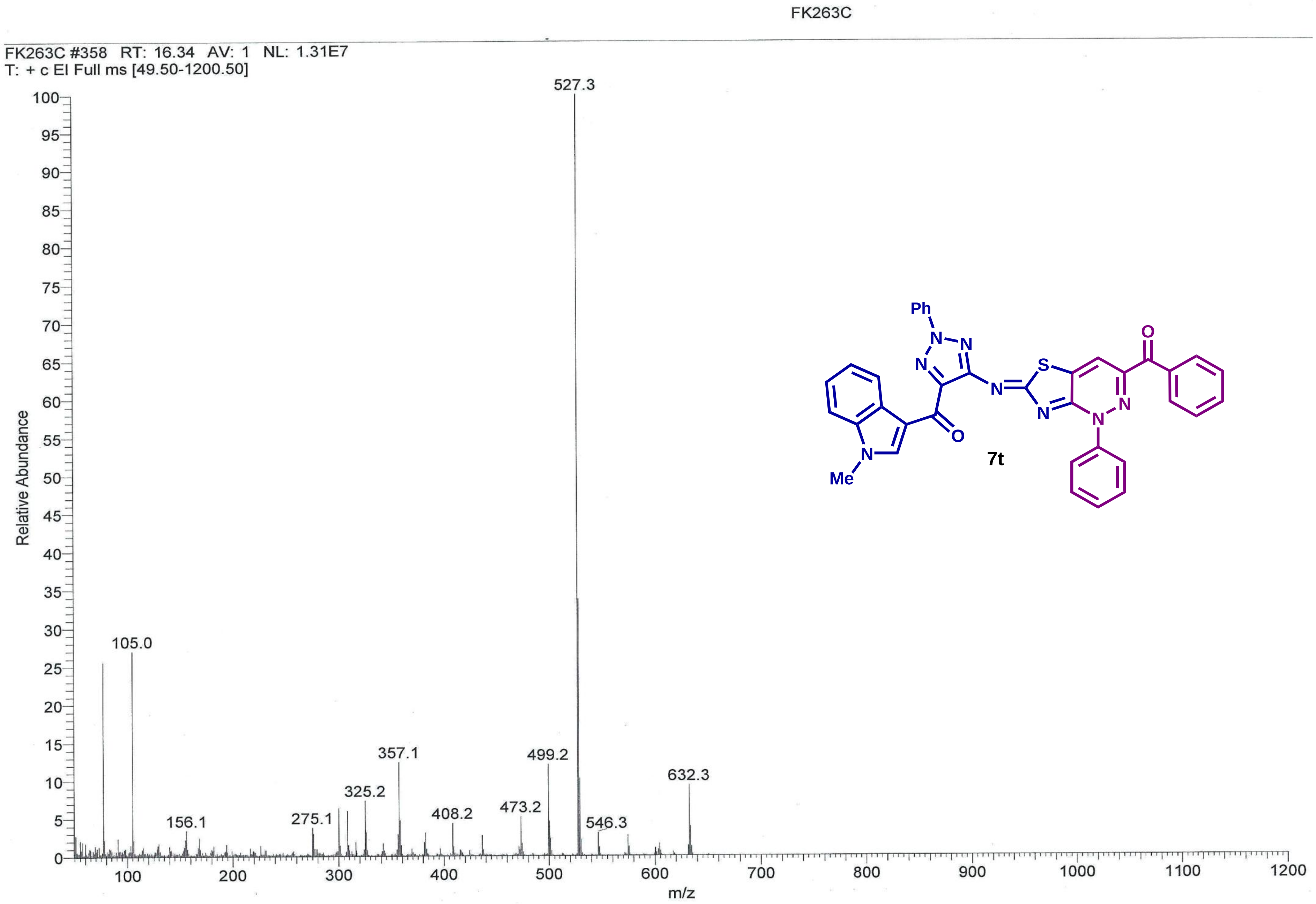

Figure S77. Mass Spectra for compound **7t**.

FK263

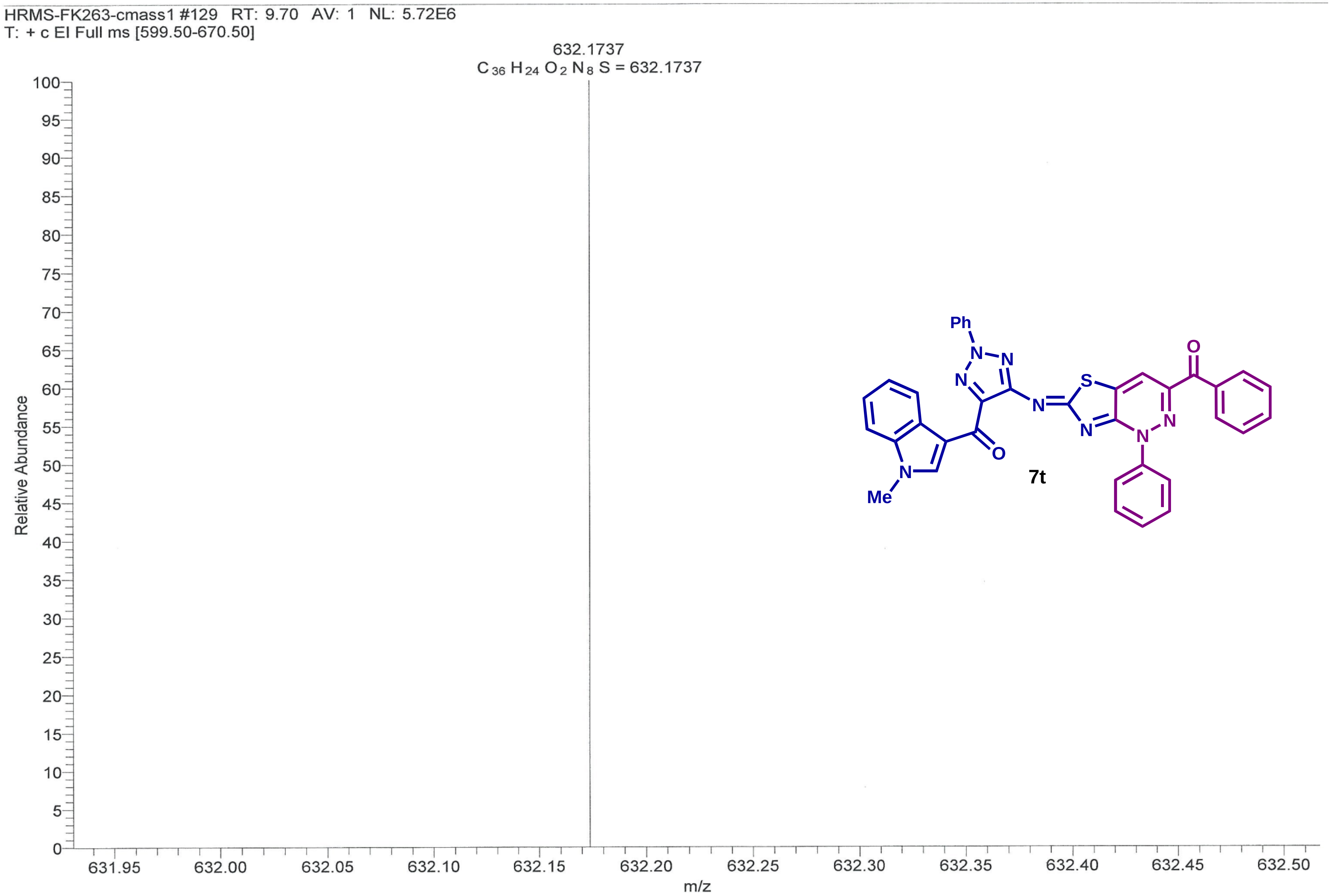

Figure S78. HRMS Spectra for compound **7t**.

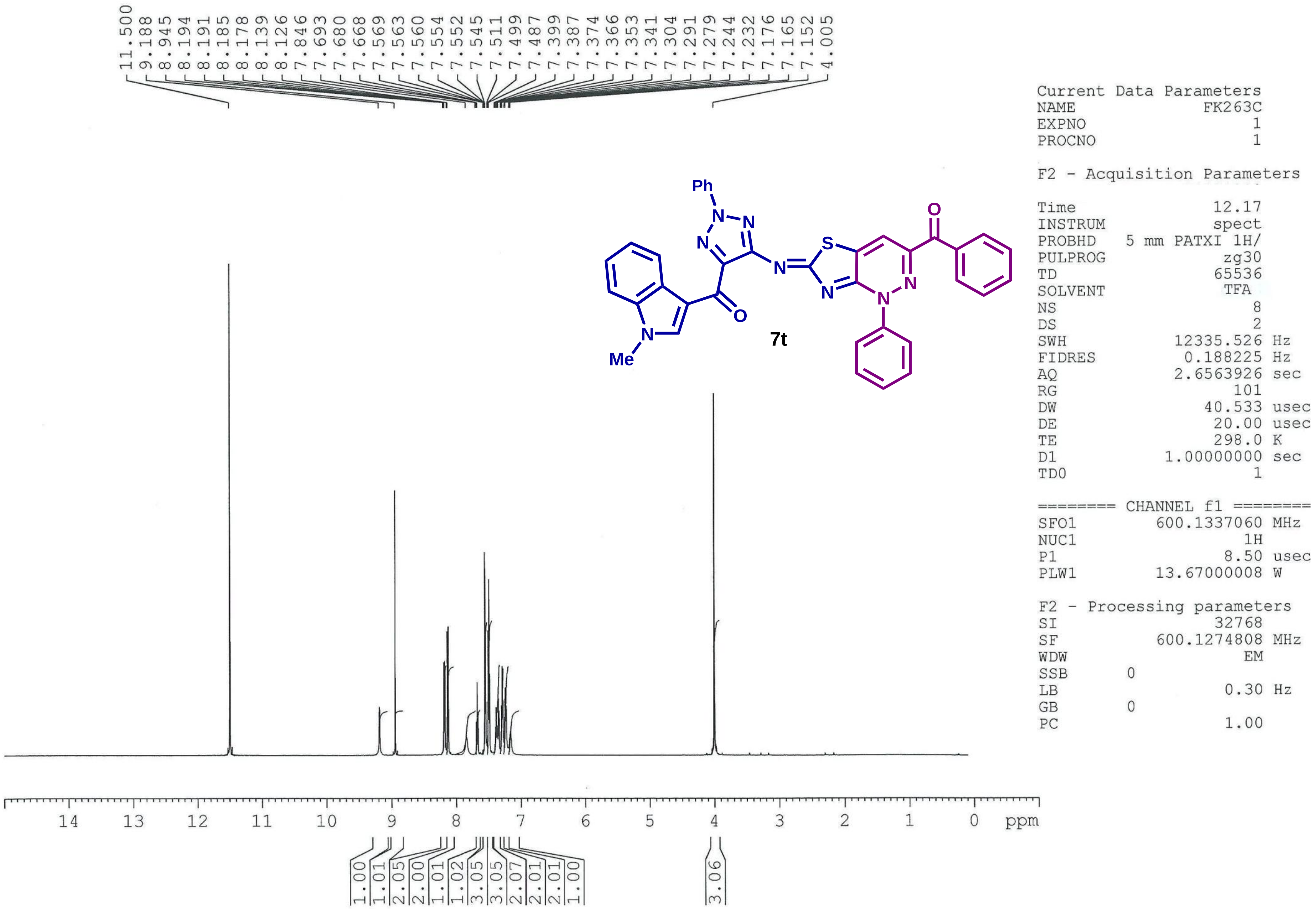

Figure S79. ^{1}H NMR Spectra (TFA-*d*, 600 MHz) for compound **7t**.

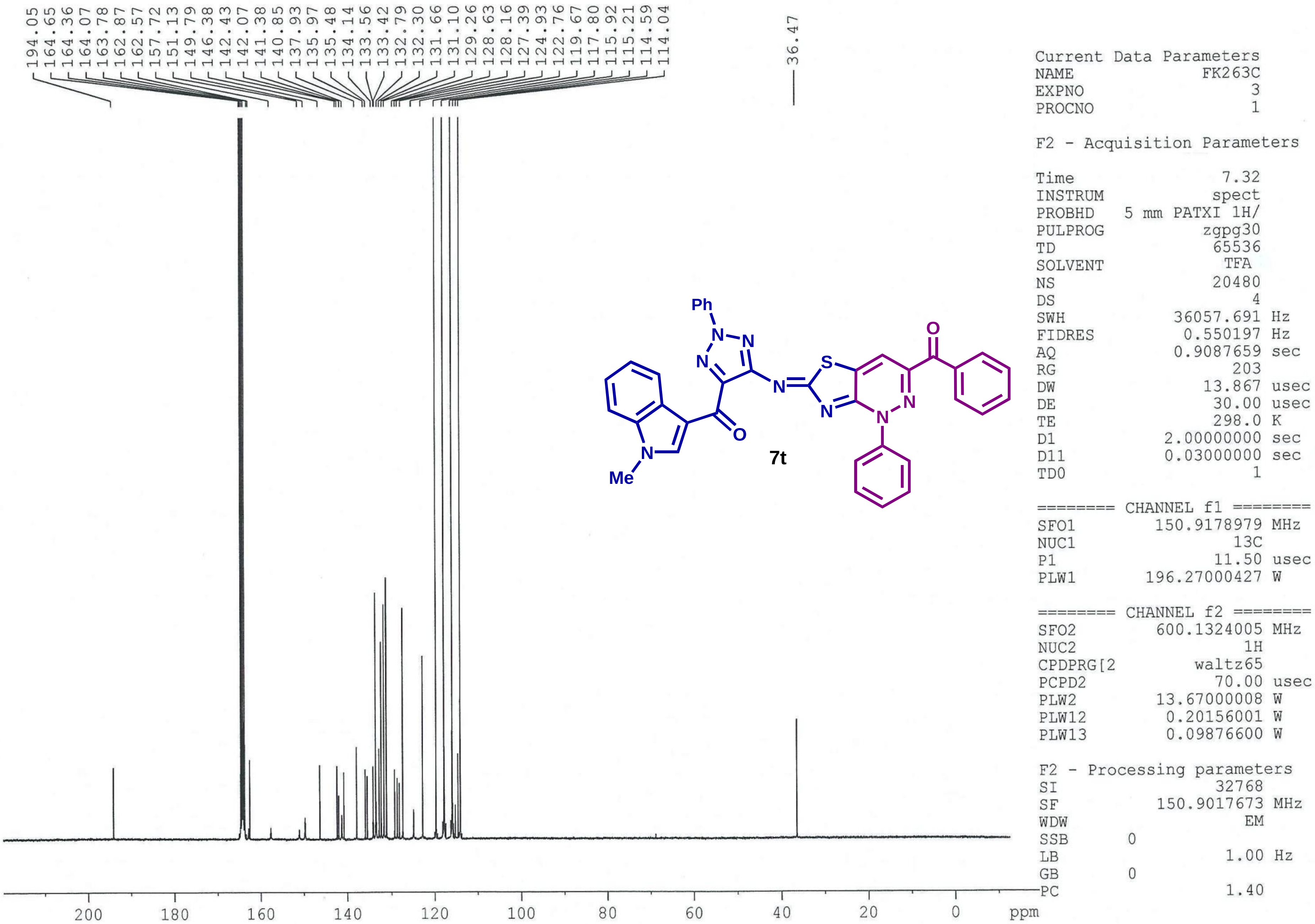

Figure S98. ^{13}C NMR Spectra (TFA-*d*, 150 MHz) for compound **7t**.

FK265C

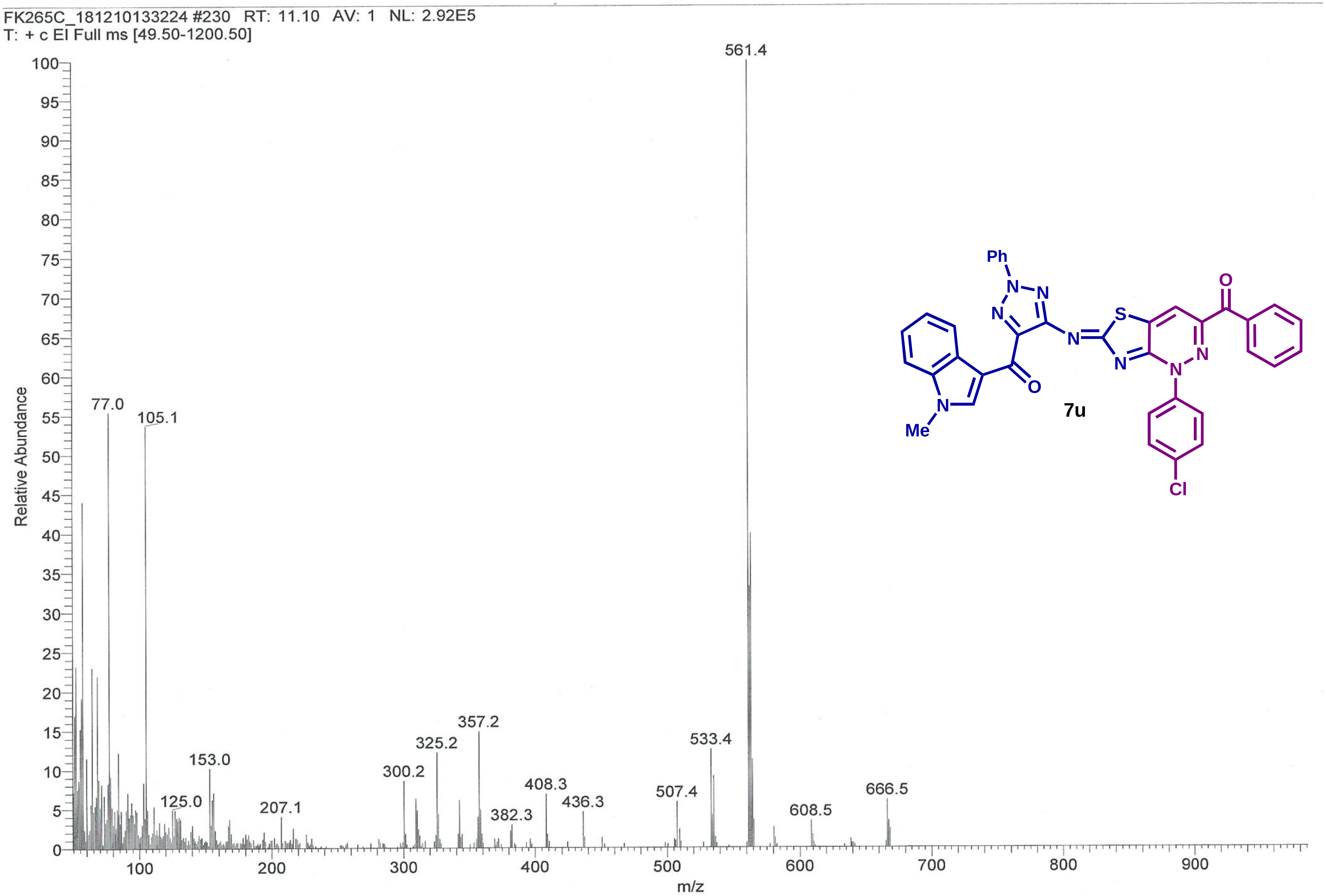

Figure S81. Mass Spectra for compound **7u**.

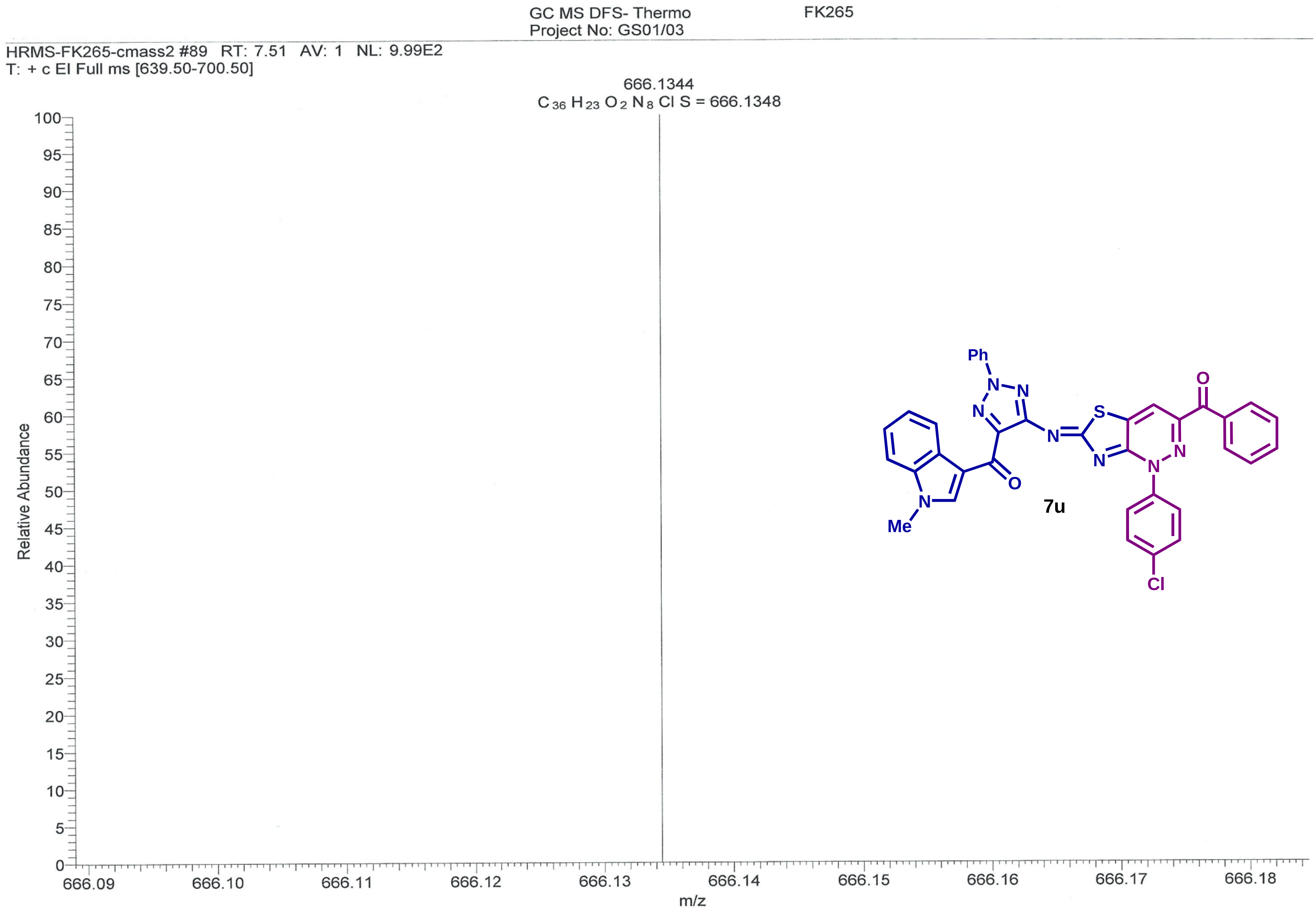

Figure S82. HRMS Spectra for compound **7u**.

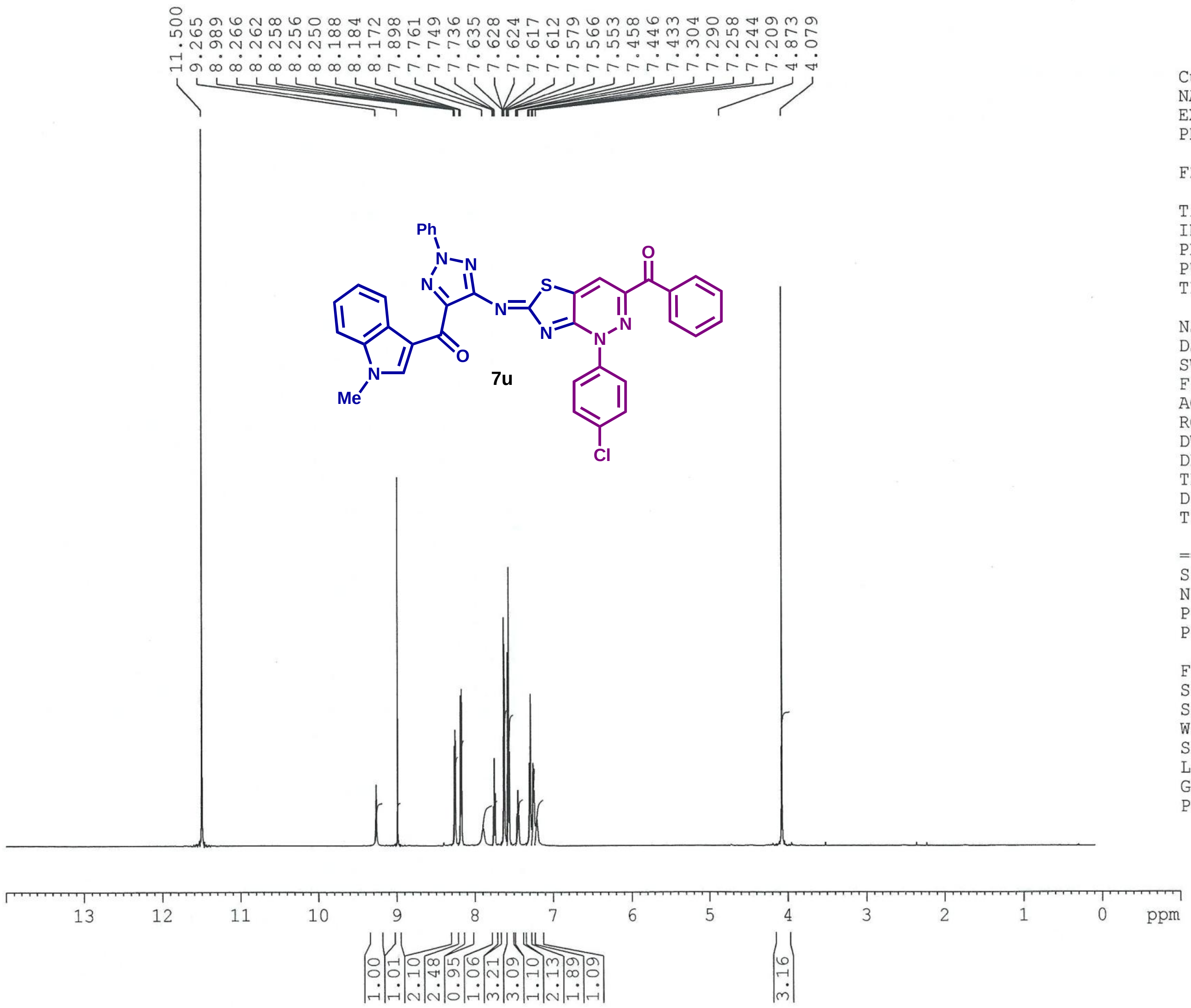

Current Data Parameters
NAME FK265
EXPNO 1
PROCNO 1

F2 - Acquisition Parameters

Time 9.56
INSTRUM spect
PROBHD 5 mm PABBO BB-
PULPROG zg30
TD 65536

NS 8
DS 2
SWH 12335.526 Hz
FIDRES 0.188225 Hz
AQ 2.6563926 sec
RG 203
DW 40.533 usec
DE 20.00 usec
TE 298.0 K
D1 1.00000000 sec
TD0 1

======== CHANNEL f1 ========
SFO1 600.1337060 MHz
NUC1 1H
P1 10.60 usec
PLW1 27.82500076 W

F2 - Processing parameters
SI 32768
SF 600.1274847 MHz
WDW EM
SSB 0
LB 0.30 Hz
GB 0
PC 1.00

Figure S83. ^{1}H NMR Spectra (TFA-*d*, 600 MHz) for compound **7u**.

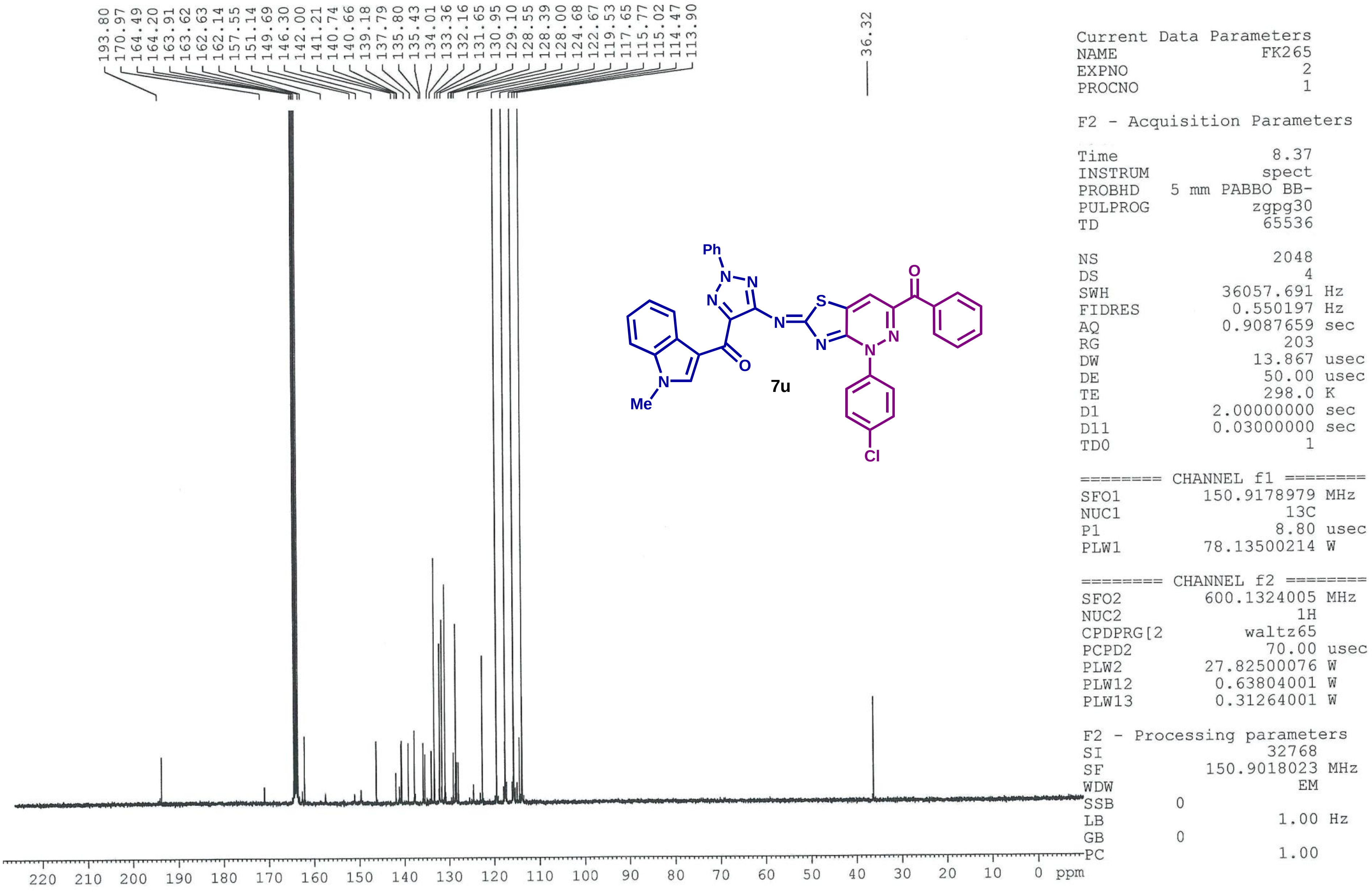

Figure S84. ^{13}C NMR Spectra (TFA-*d*, 150 MHz) for compound **7u**.
